# Machine Learning-Assisted Engineering of Light, Oxygen, Voltage Photoreceptor Adduct Lifetime

**DOI:** 10.1021/jacsau.3c00440

**Published:** 2023-11-21

**Authors:** Stefanie Hemmer, Niklas Erik Siedhoff, Sophia Werner, Gizem Ölçücü, Ulrich Schwaneberg, Karl-Erich Jaeger, Mehdi D. Davari, Ulrich Krauss

**Affiliations:** † Institute of Molecular Enzyme Technology, Heinrich Heine University Düsseldorf, Wilhelm Johnen Strasse, Jülich 52426, Germany; ‡ Institute of Bio-and Geosciences IBG 1: Biotechnology, Forschungszentrum Jülich GmbH, Wilhelm Johnen Strasse, Jülich 52426, Germany; § Institute of Biotechnology, RWTH Aachen University, Worringer Weg 3, 52074 Aachen, Germany; ∥ DWI-Leibniz Institute for Interactive Materials, Forckenbeckstraße 50, 52074 Aachen, Germany; ⊥ Department of Bioorganic Chemistry, Leibniz Institute of Plant Biochemistry, Weinberg 3, 06120 Halle, Germany; # Department of Biochemistry, University of Bayreuth, 95447 Bayreuth, Germany

**Keywords:** machine learning, light, oxygen, voltage domain (LOV), data-driven protein design, protein design, protein engineering, optogenetics

## Abstract

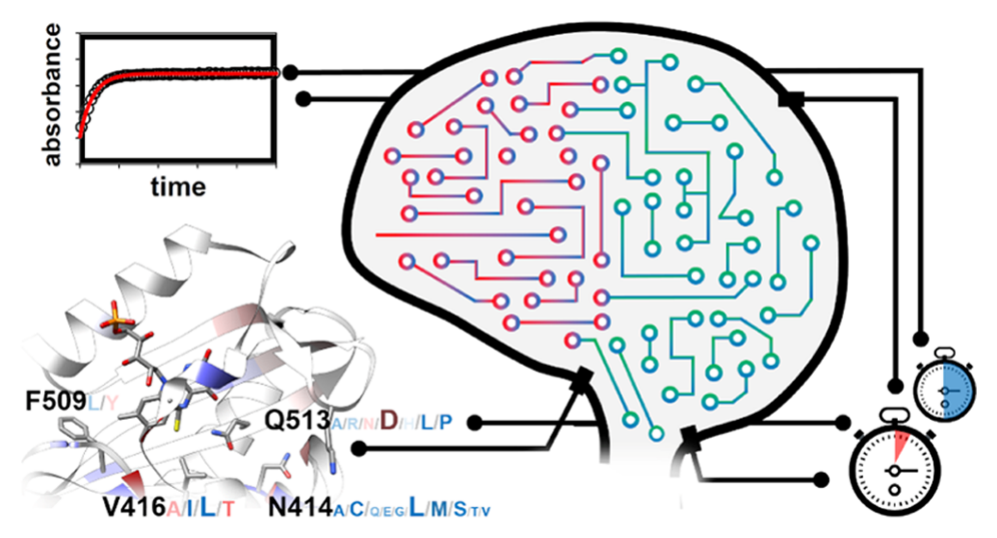

Naturally occurring and engineered flavin-binding, blue-light-sensing, light, oxygen, voltage (LOV) photoreceptor domains have been used widely to design fluorescent reporters, optogenetic tools, and photosensitizers for the visualization and control of biological processes. In addition, natural LOV photoreceptors with engineered properties were recently employed for optimizing plant biomass production in the framework of a plant-based bioeconomy. Here, the understanding and fine-tuning of LOV photoreceptor (kinetic) properties is instrumental for application. In response to blue-light illumination, LOV domains undergo a cascade of photophysical and photochemical events that yield a transient covalent FMN-cysteine adduct, allowing for signaling. The rate-limiting step of the LOV photocycle is the dark-recovery process, which involves adduct scission and can take between seconds and days. Rational engineering of LOV domains with fine-tuned dark recovery has been challenging due to the lack of a mechanistic model, the long time scale of the process, which hampers atomistic simulations, and a gigantic protein sequence space covering known mutations (combinatorial challenge). To address these issues, we used machine learning (ML) trained on scarce literature data and iteratively generated and implemented experimental data to design LOV variants with faster and slower dark recovery. Over the three prediction–validation cycles, LOV domain variants were successfully predicted, whose adduct-state lifetimes spanned 7 orders of magnitude, yielding optimized tools for synthetic (opto)biology. In summary, our results demonstrate ML as a viable method to guide the design of proteins even with limited experimental data and when no mechanistic model of the underlying physical principles is available.

## Introduction

**Figure 1:**
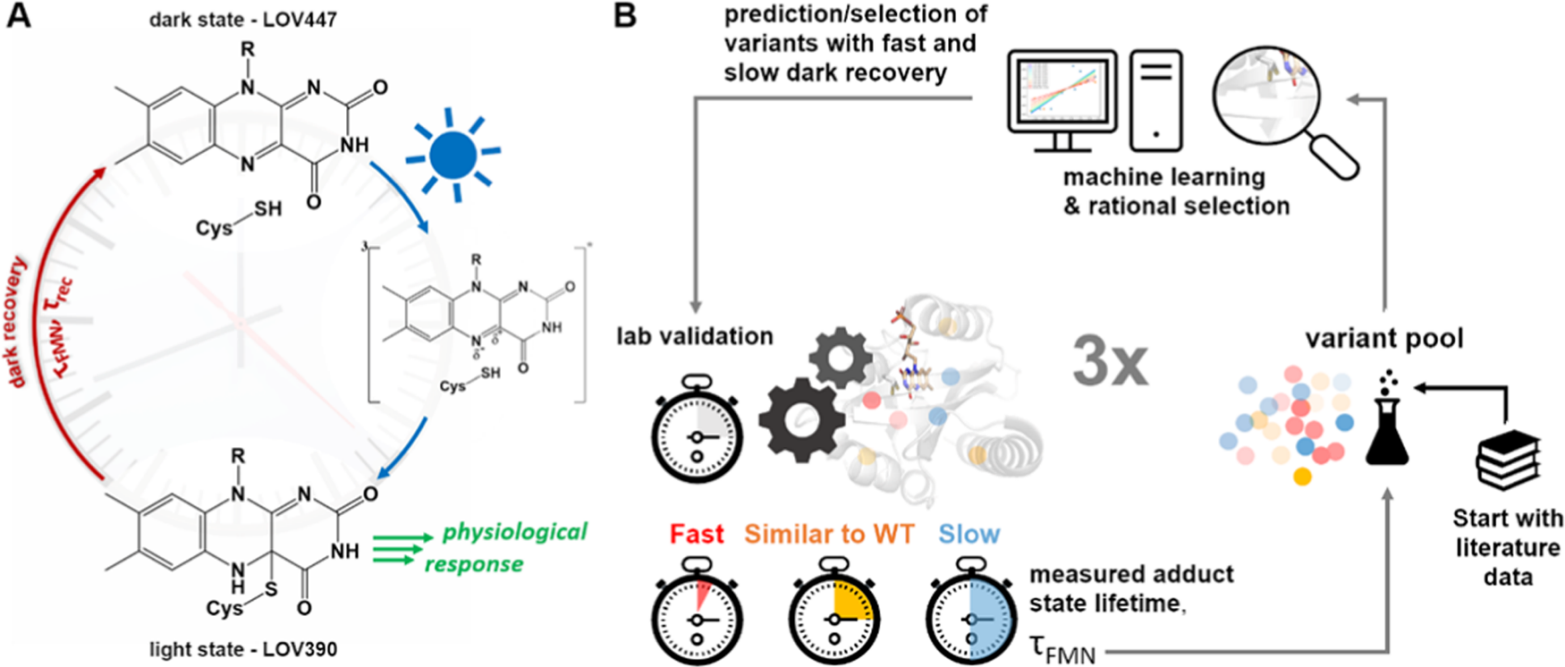
Illustration of the experimental strategy. (A) Photocycle of LOV domains. In the dark state, the oxidized flavin chromophore (FMN: R: C_5_H_12_O_7_P) is noncovalently bound in the LOV sensor domain (protein/FMN complex maximally absorbing at λ_max_ = 447 nm; LOV447). Illumination with blue light forms a transient excited singlet state (S_1_, not shown), from which an excited triplet state ^3^[FMN] is formed by intersystem crossing. Starting from the excited triplet state, a covalent bond is formed between a strictly conserved cysteine residue in the protein and the C4A atom of the flavin-ring system, with the FMN-N5 atom becoming protonated, giving rise to the so-called light or signaling state (protein/FMN complex maximally absorbing at λ_max_ = 390 nm; LOV390). Signaling-state formation is accompanied by structural changes in the sensory domain, eventually triggering the light-dependent physiological response. The photocycle is thermally reversible within a protein-specific time (dark recovery; τ_rec_), which involves breaking of the FMN-cysteinyl thiol adduct, accompanied by recovery of the flavin absorption at 450 nm (τ_FMN_), but also reversal of the structural changes associated with signaling-state formation. (B) Workflow of the three rounds of selection and ML-based prediction by PyPEF. Double and triple-substituted variants were predicted by ML, while single-substituted variants were selected from the set of predicted double and triple variants. Lab-validated variants and corresponding measured fitness values were added to the variant pool in each prediction and validation cycle.

Biological entities harbor different photoreceptor protein systems, which, e.g., can be grouped according to the wavelength of the light they absorb or respond to.^1−3^ Apart from being instrumental in sustaining life and controlling various biological functions, photoreceptors have recently also been used as synthetic molecular switches to artificially control biological functions, i.e., in the framework of optogenetics.^4,5^ In this regard, light, oxygen, voltage (LOV) blue-light photoreceptors, which are ubiquitously distributed throughout the three kingdoms of life,^6,7^ have been widely studied^6,8−10^ and used as optogenetic tools.^5,11−13^ LOV domains, the sensory modules of LOV photoreceptors, noncovalently bind oxidized flavins as chromophore,^6^ with the absorption of UV-A/blue light by the chromophore triggering a cascade of photophysical/photochemical events (Figure 1a), which eventually lead to structural changes in the photoreceptor, such as adduct formation between a conserved cysteine and the flavin chromophore, accompanied by protonation of the FMN-N5 atom. This process, depending on the photoreceptor, results in an unfolding/unwinding of N- and C-terminal helices outside the sensory LOV domain, the dissociation of adjacent domains, or changes in the oligomerization state,^2,6,9,10^ which in turn mediates various physiological responses.^9,14−20^ The photocycle is thermally reversible in the dark, with the recovery process involving the breaking of a covalent FMN-cysteinyl thiol bond, the deprotonation of the flavin N5 atom, as well as the structural reversal of the above-described light-induced structural changes of the photoreceptor. While FMN-Cys adduct formation is completed within microseconds (see e.g.,^21−24^), the dark recovery process can last from seconds to days, depending on the LOV protein.^23,25−30^ Recent studies, e.g., using time-resolved small-angle X-ray scattering techniques, have shown that the dark recovery (described by the signaling-state lifetime τ_rec_), as a whole, is a complex, multistep process that cannot be simply captured by a single technique,^31−33^ and a differentiation between the dark recovery of the FMN absorbance (τ_FMN_) as a proxy of FMN-Cys adduct rupture and the reversal of structural changes is necessary. Due to the huge variability in kinetics found in natural LOV photoreceptor systems, the dark-recovery process is of fundamental interest from a biophysical and biochemical perspective. In addition, rational tuning of the dark recovery also has important functional implications for designing optogenetic tools^34−36^ and natural photoreceptors for a plant-based bioeconomy.^37^ Mechanistically, the dark-recovery process and how it is modified by the protein environment in LOV sensor domains is still not completely understood, and various mechanisms that tune the dark recovery in LOV domains have been presented, highlighting the role of the hydrogen bond or salt-bridge network surrounding the chromophore,^27,38−41^ steric interactions affecting the conformation of the adduct forming cysteine, as well as the conformational freedom of the flavin,^25,42−44^ and last, factors that influence the deprotonation of the flavin N5 atom, e.g., pH, buffer, imidazole, hydration or water/solvent access to the flavin.^45−49^ Despite all those efforts, no conclusive mechanistic model that accounts for all observed mutational effects has yet been presented, and combinatorial effects, with some exceptions,^34^ remain poorly explored.

While mechanistically driven site-directed mutational studies of the dark recovery of LOV photoreceptors are widespread,^25,27,44,46,49,50^ large-scale random mutagenesis or directed evolution campaigns targeting the dark recovery of LOV domains remain scarce and limited to slower-reverting variants,^27^ or rely on specialized equipment.^51^ In addition, due to the long time window of at least seconds in which the recovery occurs, atomistic simulations that would cover the dark-recovery process are not feasible. Thus, based on the limited data available, the rational selection of combinatorial mutations that tune the dark recovery in a predictable fashion is challenging. Besides, the selection of identified variants for rational recombination often relies on the assumption of simple additive effects on protein fitness.^52^ Protein fitness here refers to any protein properties essential for a protein to perform its intended functions effectively. This fitness is a measure of how well a given protein performs a target function and not the typical organismal fitness concept used in evolutionary biology. A protein’s “fitness landscape” is a graphical representation illustrating how a specific protein property varies based on changes in its amino acid sequence.^53^ In this study, the fitness of a variant defines the specific experimentally determined trait of that variant: here, the FMN-Cys adduct lifetime τ_FMN_ of the respective variants.

To fill the aforementioned gap, and restrict the screening effort, machine learning (ML) methods appear ideally suited, as they employ already existing mutational data to predict protein functions without the need for a detailed physical model.^54^ We have here used ML methods to predict combinatorial variants with both faster and slower dark recovery relying on a limited set of mutational literature data for the *Avena sativa* phototropin 1 LOV2 domain (AsLOV2),^44,51^ which is one of the best studied LOV domains in terms of photocycle dark-recovery kinetics.^42−45,49−51^ At the same time, this LOV protein represents perhaps the single most widely used blue-light-dependent sensor domain employed for the construction of optogenetic tools.^5^ Over three prediction–validation cycles, LOV domain variants were successfully predicted and generated, whose FMN-Cys adduct lifetimes spanned 7 orders of magnitude (from 0.4 to approximately 100 × 10^3^ s), yielding, to the best of our knowledge, the so far fastest and slowest AsLOV2 variants described in the literature. Based on this data, potential (cooperative) mechanisms that tune the adduct-state lifetime in LOV domains, contributing to kinetic variability, are discussed.

## Methods

### Model Protein Selection, Data Collection, and Curation of Literature Data

For engineering purposes, we selected the LOV2 domain of *A. sativa* phototropin 1 (PDB ID: 2V1A, resolution: 1.65 Å;^55^) and used an expression construct that covered both the N-terminal A′α helix as well as the C-terminal Jα-helix (residues 404–546 of full-length phototropin 1; construct identical to ref (55)). AsLOV2 has a canonical LOV photocycle displaying a comparatively fast FMN-Cys adduct rupture (τ_FMN_ = 37–81 s) (see Table S1),^43,44,49−51,55−57^ and a multitude of mutational studies have been conducted to tune and analyze its dark-recovery process.^42−45,49−51,57^ At present, to the best of our knowledge, the AsLOV2 variants displaying the slowest and fastest dark recovery in terms of τ_FMN_ are the N414 V variant (τ_FMN_ > 43,200 s)^58^ and the N449S mutant (τ_FMN_ = 0.99 s).^43^ To reduce the impact of different experimental conditions impacting our predictions, we collected sequence-fitness data from several data sets collected under similar conditions.^43,44,49−51,56−58^ The fitness of a variant defines the specific experimentally determined trait of that variant: here, as a fitness parameter, the FMN-Cys adduct lifetime τ_FMN_ of the respective variants was used. The final data set contained 110 variant entries with 80 unique single-site variants covering 66 amino acid positions, 6 double, 1 triple, and 2 quadruple variants, resulting in adduct-state lifetimes that varied over 3 orders of magnitude (Table S1). For regression modeling, different data set splits were applied for training and testing models for the rounds of ML-based prediction and lab-based validation. All data set splits used for model training and testing for each round of prediction and lab-based validation are provided in the Supporting Information, Table S1.

### Machine Learning-Guided Protein Design

ML predictions were performed using the PyPEF^59^ data-driven protein engineering framework. PyPEF converts variant sequences using amino acid index sets taken from the AAindex database^60^ (sequence encoding) and validates model performances by iterating over the 566 available physicochemical descriptor sets to find trustworthy models for generalization to estimate the fitness of new variants. This procedure can be interpreted as an optimization of the amino acid index taken for encoding sequences based on the predictions of the entries of the test set to find an index that is reliable for model generalization. Besides, the MERGE modeling method^61^ was tested, which uses an evolutionary sequence encoding technique for training regression models combined with a statistical predictor. Both modeling methods are available in PyPEF (https://github.com/Protein-Engineering-Framework/PyPEF), and additional information about selected framework parameters are provided in the Supporting Information (Supporting Texts S1 and S2).

### Bacterial Strains and Plasmids

All bacterial strains and plasmids that were used or generated in this study are listed in Table S9. *Escherichia coli* DH5, *E. coli* BL21(DE3), and *E. coli* CmpX131^62^ were used for cloning purposes and heterologous overexpression, respectively.

### Generation of AsLOV2 Variants

All AsLOV2 variants (Table S9) used in this study were generated by site-directed mutagenesis using the Quikchange PCR method. Each PCR reaction (50 μL) contained 0.1 μM oligonucleotides (Table S10), 200 pg/μL template, 200 μM desoxynucleotide triphosphates (dNTPs) (Thermo Fisher Scientific, Waltham, MA), 1 U phusion-DNA-polymerase (Thermo Fisher Scientific, Waltham, MA), 5% (v/v) DMSO (Roth, Karlsruhe, Germany), and 5x GC-phusion buffer (Thermo Fisher Scientific, Waltham, MA). For amplification, the following temperature program was used: initial denaturation for 30 s at 98 °C. Denaturation for 30 s at 98 °C, followed by an annealing step of 1 min at a temperature depending on the individual melting temperatures of the employed oligonucleotides and an elongation step at 72 °C for 4 min. Denaturation, annealing, and elongation steps were repeated 20 times, followed by a final elongation step at 72 °C for 5 min. Methylated parental template DNA was digested with DpnI at 37 °C overnight, followed by heat inactivation at 75 °C for 15 min. The resulting reactions were used for transforming *E. coli* DH5α cells. Plasmid DNA was prepared by using the innuPREP Plasmid Mini Kit (Analytik Jena, GmbH, Jena, Germany) according to the instructions of the manufacturer. All AsLOV2 variants were verified by sequencing (SeqLab, Göttingen, Germany).

### Heterologous Gene Expression

The expression of all AsLOV2 variants was performed using *E. coli* BL21(DE3) (Table S9) with pET28a as an expression vector. The AsLOV2-N414L/V416L variant was additionally expressed in *E. coli* CmpX131 (Table S9), a riboflavin-auxotrophic strain, which can be supplemented with riboflavin during growth to improve flavin mononucleotide (FMN) loading. To enable protein purification by immobilized metal ion affinity chromatography (IMAC), all constructs were fused to an N-terminal hexahistidine tag (sequence: MGSSHHHHHHSSGLVPRGSH), derived from the pET28a expression plasmid. *E. coli* BL21(DE3) cells, transformed with the corresponding expression plasmids (Table S9), were grown on a 500 mL scale using lysogeny broth (LB) medium (10 g/L NaCl, 10 g/L tryptone, and 5 g/L yeast extract) under selection pressure (kanamycin 50 μg/μL). *E. coli* BL21(DE3) expression cultures were inoculated with overnight grown precultures to an OD_600nm_ of 0.05 and cultivated at 37 °C and 120 rpm until they reached an OD_600nm_ of 0.5. Subsequently, gene expression was induced with 0.1 mM IPTG, and cultivation was continued at 15 °C and 120 rpm overnight. *E. coli* CmpX131 cells, transformed with the plasmid carrying the gene coding for AsLOV2-N414L/V416L (Table S9), were grown on a 500 mL scale using autoinduction medium (24 g/L yeast extract, 12 g/L casein hydrolysate, 54 mM K_2_HPO_4_, 16 mM KH_2_PO_4_, 4 mL/L glycerol, 20 g/L lactose, 50 g/L glucose) under selection pressure (kanamycin 50 μg/μL). Cultures were supplemented with 50 μM riboflavin. Expression cultures were inoculated with overnight grown precultures to an OD_600nm_ of 0.05 and cultivated at 37 °C and 120 rpm until they reached an OD_600nm_ of 0.5. Cultivation was then continued at 25 °C and 120 rpm for an additional 70 h. Cells were harvested by centrifugation at 4 °C and 6000*g* for 20 min and stored at −18 °C until further use.

### Protein Purification and Sample Preparation

Frozen cell pellets were thawed and resuspended as a 10% (w/v) cell suspension with Ni-NTA lysis buffer (50 mM Na_2_HPO_4_, 300 mM NaCl; pH 8.0) and disrupted by using a high-pressure EmulsiFlex-C5 homogenizer (Avestin, Ottawa, Ontario, Canada) in four cycles at 1000–1500 bar. Cell debris and unbroken cells were sedimented by centrifugation at 4 °C and 20,000*g* for 20 min, and the supernatant was used for protein purification. Protein purification was performed using a self-packed IMAC gravity flow column (size: 1.5 cm × 2.8 cm, gel bed volume: 5 mL, Sarstedt, Nümbrecht, Germany) using Ni-NTA Superflow resin (QIAGEN, Hilden, Germany) as a column material. The column was equilibrated with two-three column volumes of Ni-NTA lysis buffer. After washing with 50 mL of Ni-NTA wash buffer (50 mM Na_2_HPO_4_, 300 mM NaCl, 20 mM imidazole; pH 8.0), the target protein was eluted with 30 mL of Ni-NTA elution buffer (50 mM Na_2_HPO_4_, 300 mM NaCl, 250 mM imidazole; pH 8.0). The purified protein samples were concentrated at 15 °C and 518*g* using centrifugal concentrator units (10 kDa molecular weight cutoff Macrosep Advance Centrifugal Device; Pall Corporation, Port Washington, New York) in a swing-out rotor of a refrigerated centrifuge, to a total volume of 2.5 mL. The concentrated protein samples were desalted using PD-10 desalting columns (size: 1.4 cm × 5 cm, gel bed volume: 8.3 mL; GE-Healthcare, Chicago, Illinois) and transferred to storage buffer (50 mM Tris, 1 mM EDTA, 5 mM DTT; pH 8.0). PD-10 columns were equilibrated, used, and purified according to the manufacturer’s instructions. Protein quality and purity were estimated from denaturing SDS-PAGE gels^63^ (see Figures S22–S29). All samples were stored in the dark at room temperature for 1 day to allow for dark recovery of the protein. Subsequently, the samples were snap-frozen in liquid nitrogen and stored at −18 °C until further use.

### UV–Vis Spectroscopy to Determine Sample Quality, Photocycling, and Flavin Loading

All sample handling steps were performed under dim-red safety light for maintaining the proteins in their dark state. Measurements were carried out using semi micro quartz cuvettes with a path length of 10 mm using a Cary-60 UV/Vis spectrophotometer (Agilent Technologies, Santa Clara, CA) equipped with a single-cell Peltier-controlled cuvette holder thermostated to 25 °C. Samples were diluted with storage buffer (50 mM Tris, 1 mM EDTA, 5 mM DTT; pH 8.0) to an OD_450nm_ of 0.1. One milliliter of storage buffer served as a solvent blank. Initially, the dark-state spectrum was recorded in the range of 250–600 nm. Subsequently, the samples were illuminated for 1 min with a blue-light LED (λ = 440 nm, 2.6 mW cm^–2^^64^ Luxeon Lumileds, Philips; Aachen, Germany), and the light-state spectrum of the protein was recorded. Protein concentration and chromophore loading was estimated from the dark-state spectrum.^65^ Chromophore loading can hereby be determined using the following equations
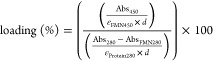
1with Abs_450_ being the measured absorption at 450 nm, ε_FMN450_ the molar extinction coefficient of FMN at 450 nm, *Abs*_280_ the measured absorption at 280 nm, Abs_FMN280_ the contribution of the flavin to the absorption at 280 nm (determined using eq 2), and ε_Protein280_ the molar extinction coefficient of the protein determined based on the amino acid sequence and *d* being the path length.
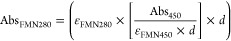
2The extinction coefficients of FMN at 280 and 450 nm were determined experimentally to be ε_FMN280_ = 18,107 M^–1^ cm^–1^ and ε_FMN450_ = 11,765 M^–1^ cm^–1^.

Protein concentrations (*c*_Protein_) were determined from the measured absorbance of the protein samples at 280 nm (Abs_280_), corrected by the absorbance of the bound FMN at 280 nm (Abs_280_, determined according to eq 2)
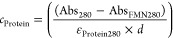
3with ε_Protein280_ being the molar extinction coefficient of the respective proteins at 280 nm (see Supporting Information, Table S4–S8), which was calculated using the Internet tool ProtParam^66^ [http://web.expasy.org/protparam], which calculates ε_Protein280_ based on the amino acid sequence of the respective protein.

### UV/Vis Spectroscopic Dark Recovery Measurements

Dark-recovery measurements were performed using the same Cary-60 spectrophotometer, cuvettes, and buffers for dilutions, as described above (see UV–vis spectroscopy to determine sample quality, photocycling, and flavin loading). Before each dark-recovery measurement, the samples were illuminated in a cuvette, as described above. Measurement strategies, outlined in the following, varied between different sample types, e.g., depending on whether the variants showed a very fast or very slow recovery or tended to aggregate.

#### Fast-Recovering, Stable Variants

The dark recovery of the protein was followed by continuously measuring the absorbance at 485 nm over a protein-specific period of time. To check for protein aggregation during the measurement time, the same measurement (identical measurement time) was carried out by measuring the absorbance at 550 nm. Here, an increase in absorbance at 550 nm served as an indicator of incubation-time-dependent protein aggregation. All measurements were performed in triplicate. For data evaluation, the absorbance values at 485 nm (Abs_485_) were plotted against time. The resulting experimental data were fitted to either a one-phase or two-phase exponential decay function to determine the FMN-absorption recovery (characterized by τ_FMN_) as a proxy for the FMN-Cys adduct lifetime^46^
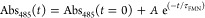
4.1
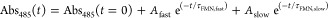
4.2with τ_FMN,slow_, τ_FMN,fast_, *A*_slow_, and *A*_fast_ representing the adduct-state lifetimes and amplitudes of the fast and slow components, respectively. The quality of each fit was evaluated on the basis of the residuals, i.e., the discrepancy between experimental data and fit. To enable the inclusion of τ_FMN_ data for variants showing a two-phase decay in the next ML prediction round, as well as for plotting and mapping purposes, an average τ_FMN,ave_ was calculated (τ_FMN,ave_ = ∑(*A*_*i*_τ_*i*_)/100) (see Tables S4–S8). For those variants, in addition, both time constants are given in Table S13.

#### Slow Recovering, Aggregation-Prone AsLOV2 Variants

Some variants showed very slow dark recovery and concomitant protein aggregation (AsLOV2-N414A/V416A, AsLOV2-N414A/V416L, AsLOV2-N414L/V416L, and AsLOV2-N414L/Q513L; see Table S12). We therefore followed the dark recovery of these variants by recording full UV/Vis spectra during dark recovery. The resulting spectra, in turn, contain contributions from both spectral dark recovery and protein aggregation and can be corrected by fitting a suitable empirical function to the spectra (see below)(see Figure S17). The dark recovery of each protein was measured over a protein-specific period of time in the wavelength range 250–600 nm until the protein returned completely to its dark state. All measurements were performed in triplicate.

To determine the FMN-Cys adduct-state lifetime (τ_FMN_) from the kinetic data, we plotted all recorded spectra. Spectral recovery data was fitted using an empirical function in the form *Y* = *Y*_0_ + *ax*^–*n*^, with *x* representing the wavelength and *n* varied between 1 and 3, to account for increased scattering due to protein aggregation. The best fitting function was determined from reproduction of typical isosbestic points (at around 387 and 409 nm) usually found in LOV domain dark-recovery data.^57^ Measured spectra were corrected by subtracting the scattering contribution from the data. Subsequently, absorbance values at 485 nm were extracted from the corrected spectra and plotted against time. The resulting experimental data were fitted to one or two-phase exponential decay functions to determine τ_FMN_ (eqs 4.1 and 4.2; see above).

### Temperature-Dependent Unfolding Experiments

The temperature-dependent unfolding, as a proxy for thermal stability, was determined by using differential scanning fluorimetry (DSF), by employing a Prometheus NT.Flex (NanoTemper Technologies GmbH, Munich, Germany) instrument. DSF monitors the unfolding of proteins by detecting temperature-dependent changes in aromatic amino acid fluorescence. Purified AsLOV2 samples (wild type and selected variants) (10 μL) with a concentration of 0.4–0.5 mg mL^–1^ were subjected to a linear unfolding ramp (1 °C min^–1^, from 20 to 95 °C). The intrinsic tryptophan fluorescence of the protein was monitored continuously (18 data points per minute) at 350 and 330 nm. Unfolding transition midpoints (expressed as melting temperature, *T*_m_) were determined from the first derivative of the fluorescence ratio (F350/F330) by using RT.ThermControl Software (NanoTemper Technologies GmbH).

### Data Visualization and Structure/Function Analyses

All FMN-Cys adduct-state lifetime (τ_FMN_) data was normalized to the data of the corresponding wild type by dividing τ_FMN_ of the respective variant by the τ_FMN_ of the wild type and expressed as log-values log (norm. τ_FMN_). This results in negative values for variants with a faster τ_FMN_ (color-coded in shades of red) and positive values for variants with a slower dark recovery (color-coded in shades of blue). Log (norm τ_FMN_) values identical to the wild type yield a value of zero (color-coded in white). To assess if a variant showed a significantly different τ_FMN_ compared to the wild type, τ_FMN_ was measured for three independent preparations of AsLOV2 wild type, which yielded a τ_FMN_ = 43 ± 4.5 s. A variant with a τ_FMN_ difference of ±2 standard deviations (σ) was thus considered to show a significantly faster or slower recovery, respectively. Mutational mapping of residue-wise fitness log (norm. τ_FMN_) data was carried out using Microsoft Excel’s conditional formatting function. For structure/function mapping purposes, we overwrote the B-factor field of the AsLOV2 dark-state structure (PDB ID: 2V1A) with the corresponding log(norm. τ_FMN_) values using a custom Perl script (https://www.iet.uni-duesseldorf.de/arbeitsgruppen/molecular-biophotonics/links-and-resources/own-scripts-for-data-analysis). In this fashion, unfortunately, only a single value can be mapped to the B-factor field of each residue. Therefore, for all residue positions where different mutations showed similar trends, e.g., in all cases yielding faster or slower variants, we averaged the respective log(norm. τ_FMN_) values and mapped the mean to the structure. For all residue positions where contrasting mutational effects were observed, e.g., with certain mutations yielded faster and other mutations at the same position slower variants, log (norm. τ_FMN_) was set to zero, and the position was highlighted in the corresponding figures as a stick model with the respective log(norm. τ_FMN_) data shown as color-coded circles with the exchange given as a one letter amino acid code. Intraprotein distances were calculated in PyMOL using the DistancesRH Python script for PyMOL written by Pietro Gatti-Lafranconi (https://www.pymolwiki.org/index.php/DistancesRH). Combinatorial mutations were visualized by generating color-coded dashed lines between the respective residue CA atoms by using a custom Python script for PyMOL (https://github.com/Protein-Engineering-Framework/scripts/tree/main/AsLOV2). For structure visualization, open source PyMOL Version 2.6.0a0 (The PyMOL Molecular Graphic System, Version 2.0 Schrödinger; LLC)^67^ or Chimera 1.16^68^ was used.

## Results and Discussion

### Machine Learning, Library Design, and Variant Characterization of LOV Domains with Fine-Tuned Dark Recovery

In recent years, ML methods have become increasingly popular in protein engineering.^54,69,70^ ML models infer patterns from a data set, which can then be used to make predictions on unobserved data, which may range from recombinants of characterized variants to the entire protein fitness landscape. Regression models are trained on a given data set in a supervised fashion to predict the fitness of a variant from its sequence. In this process, we fine-tune certain settings in the model, which we call “hyperparameters.” A hyperparameter is a parameter that is used to control the training algorithm and whose value, unlike other parameters, must be set before the model is trained. This could be, for example, the number of features to be considered for training or a regularization constant to be optimized during training. This iterative optimization ensures that our model is not only tailored to the specific characteristics of our training data but also capable of making accurate predictions on new, unseen data. The essential prerequisite for supervised model training is the availability of a suitable training data set, which can be obtained from the literature, databases, or experimentally.^71^ However, using data from the scientific literature for training ML models has a potential limitation. The data in research papers may not always be ideal for training because the studies often have specific goals or hypotheses in mind. For instance, researchers might focus on mutating only specific positions in a protein, introducing certain types of amino acids. Additionally, the experiments may have been conducted under different conditions for assessing the target trait.^61,71^ This variation in experimental conditions and selective mutations can introduce bias into the data set. Thus, ideally, unbiased mutant libraries generated and characterized by identical or similar methods under identical conditions should be used. One of the best-characterized LOV domains, for which dark recovery kinetic data is available for a large set of mutants characterized by similar methods^43,44,49−51,57,58^ (see Table S1), is the LOV2 domain of *A. sativa* phototropin 1 (AsLOV2), which shows a canonical LOV photocycle (Figure 1A) with a fast dark recovery of τ_rec_ = 37–81 s (see Table S1).^43,44,49−51,55−57^ We used the compiled sequence-fitness data from the corresponding literature data sets, where “fitness” corresponds to the FMN-Cys adduct-state lifetimes τ_FMN_ (Table S1) to construct and test ML models predicting double- (in the first two prediction and validation rounds, Table S2) and triple-substituted variants (in the last round, Table S3). In the first experimental validation round as well as in each round when novel combinatorial variants were predicted, the corresponding single-substituted variants were also experimentally generated, tested, and included in the next-round data set. In the article, we refer to those latter variants as single-site selected variants. The overall prediction–validation procedure was repeated until a total of three rounds of engineering were achieved, with the third round including prediction and validation of triple-substituted variants (see Figure 1). For further details about the ML-guided protein design and evaluation of the modeling process, see Supporting Information Texts S1 and S2, respectively.

Please note that in the present study, we solely measured the dark recovery of the FMN absorbance as a proxy of FMN-Cys adduct rupture and refer to the process interchangeably as dark recovery or FMN-Cys adduct rupture. As outlined in the introduction, dark recovery is a complex multistep process involving not only thermal rupture of the FMN-Cys adduct but also large-scale structural changes in the protein. Therefore, all kinetic data presented in the manuscript solely refers to the dark recovery of the FMN absorbance (τ_FMN_), which must not necessarily match the structural recovery kinetics of the protein.

### Global Analysis of Mutational Data Recapitulates Known Dark-Recovery Tuning Mechanisms

All predicted variants were generated by Quikchange PCR, the proteins produced in *E. coli*, purified and characterized with respect to flavin binding and dark recovery kinetics yielding the FMN-Cys adduct lifetime τ_FMN_ as fitness parameter (see Tables S4–S8 for the full data sets). All τ_FMN_ values, including compiled literature data, were mapped and analyzed at residue and structure levels to infer structure–function relationships. Please note that for certain data sets, a two-phase exponential decay appeared better suited to fit the experimental data, which might reflect the recent suggestion that the dark recovery is a complex multistep process (see above). Interestingly, it appeared that for variants that contained a mutation in N414 (to E, M, C, G, and D) or G528 (E or A) (as single, double, or in triple variants), a two-phase exponential decay function seemed to fit the experimental data better. In such cases, we calculated an average τ_FMN,ave_ as described in the Methods section and provide both time constants in the Supporting Information (Table S13).

**Figure 2:**
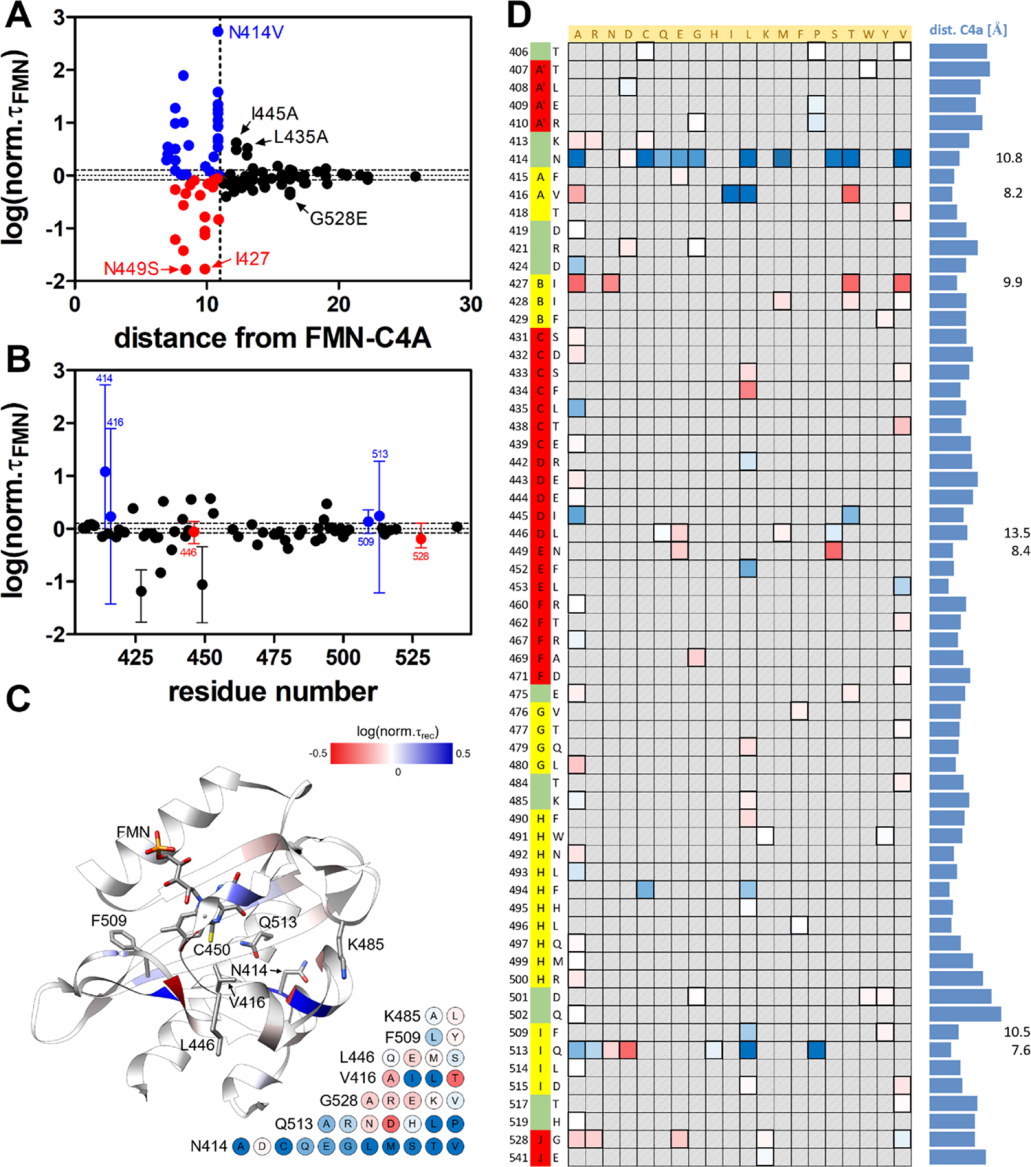
Structure–function analysis of the final AsLOV2 mutational data set. All data were transformed as described in the Methods section and are expressed as log (norm. τ_FMN_), which yields negative values for variants with a faster τ_FMN_ (color-coded in shades of red) and positive values for variants with a slower τ_FMN_ (color-coded in shades of blue). (A) Residue-wise mutational data (log (norm. τ_FMN_)) plotted against the distance between CA atom of respective residue and FMN-C4A. Filled blue- and red circles correspond to slower (blue) or faster (red) variants with a residue-FMN distance <11 Å, and filled black circles are used to denote variants with a distance of >11 Å from the chromophore. (B) Mutational data (log (norm. τ_FMN_)) plotted against the residue number. Error bars representing the data range shown for specific residues result from variations in τ_FMN_ for different mutations at the specific positions. Red and blue-filled circles mark positions for which both faster and slower variants were identified depending on the introduced mutation (highlighted also in panel C), with red and blue-filled circles indicating if the mean of the respective values yields positive or negative log (norm. τ_FMN_) values, respectively. Data sets showing either only faster or only slower variants (depicted as mean with error bars representing the standard deviation) are shown with black-filled circles. Dashed lines in A and B denote the ±2σ threshold for considering a variant as showing a faster or slower dark recovery. (C) Mutational data (log (norm. τ_FMN_)) mapped onto the structure of AsLOV2 (PDB ID: 2V1A) with positions showing, on average, faster or slower dark recovery colored in red and blue, respectively. The FMN chromophore and selected residue positions, for which both faster and slower mutants were obtained, are shown as a stick model. Amino acid residues and the FMN chromophore shown in the stick representation are colored with oxygen atoms in red, nitrogen in blue, phosphorus in orange, sulfur in yellow, and carbons in gray. In the lower right corner, mutational data are illustrated for these specific residue positions. The data is shown as color-coded circles with the exchange given as a one letter amino acid code (see text for discussion); color-coded according to log (norm. τ_rec_). (D) Heatmap illustrating the complete data set, with log (norm. τ_FMN_) values (scale: −1 < log (norm. τ_FMN_) < 1) color-coded as before. For better visibility, all positions for which data is available are highlighted with thick bordered boxes. Positions where a mutation resulted in a τ_FMN_ similar to the wild type are highlighted in white, while positions not targeted by mutagenesis are shown as gray hatched boxes. The second column illustrates the position where the respective residue is found in N- to C-terminal topological order. The last column shows the distance of the respective residue CA atom from the FMN-C4A atom.

**Figure 3:**
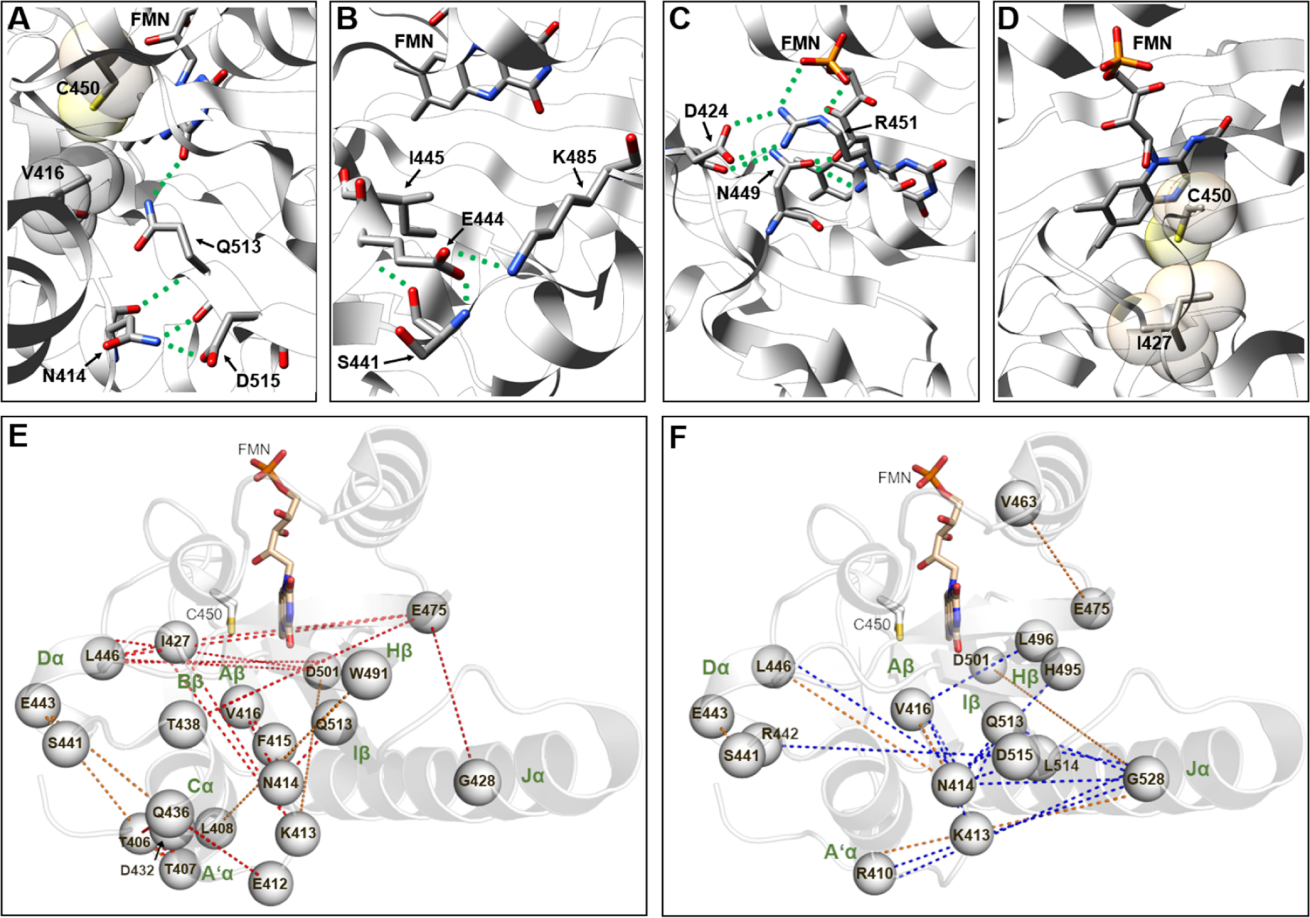
Structure–function relationships identified from single-site mutational data (A–D) and multisite mutational networks (E,F). Cartoon representation of the AsLOV2 structure (PDB ID: 2V1A) with selected residues shown in the stick representation or as a van der Waals sphere. Amino acid residues and the FMN chromophore shown in stick representation are colored with oxygen atoms in red, nitrogen in blue, phosphorus in orange, sulfur in yellow, and carbons in gray. (A) V416–C450-Q513-N414-D515-flavin site (for details see text), (B) K485-E444-S441 site (for details see text), (C) N449-FMN-ribityl interaction and Eα H-bonding network (for details see text), and (D) I427 site sterically supporting C450 in the dark state (for details see text). Green dashed lines in parts A–D denote hydrogen-bonding interactions. Mutational network illustrating multisite mutations that yield variants with a faster (E) and slower (F) dark recovery. The AsLOV2 structure is shown as a transparent cartoon, and residue positions are highlighted by a van der Waals sphere positioned at the respective CA atom. Color-coded dashed lines mark the mutational network spanning two or at most three residue positions, with red, blue, and orange dashed lines representing faster and slower-reverting variants or variants that show a recovery similar to the wild type, respectively.

In the following, we first discuss the global characteristics of published, and, newly obtained single-site mutation data (Figure 2). Interestingly, much larger effects in terms of acceleration or deceleration (magnitude of log (norm. τ_FMN_)) were observed for residues within 11 Å of the FMN chromophore (Figure 2A; Table S1). The largest single-site effects were observed for N414V, resulting in an about 530-fold slower recovery,^58^ and N449S with an about 60-fold faster recovery.^43^ For residues at the distance >11 Å of the FMN chromophore, the largest effects were observed for I445A at a distance of 12.2 Å (4-fold slower recovery; Table S1) and L435A at a distance of 13 Å (≈3-fold slower recovery;^50^) as well as for G528E at a distance of 16.3 Å (2-fold faster recovery, Table S1). Overall, significant alterations in τ_FMN_ could be observed for residues as far as 22.2 Å away (D501-CA···FMN-C4A; D501Y, 1.2-fold acceleration, Table S1). In terms of the magnitude of the observed single-site effects (Figure 2B,C) also, position N414 stands out, with the residue showing the largest mutation-dependent variability in τ_FMN_ (Figure 2B,C), which, however, might also be related to the fact that this residue has been quite often targeted by mutagenesis (Table S1).^49,50,55^ The same holds for residues Q513 and V416, and to a more limited degree, also for F509, K485, L446, and G528, which have been targeted more extensively, yielding both faster and slower variants (Table S1) (Figure 2B,C). Of these positions, N414 is found rather far away from the FMN chromophore (N414-CA···FMN-C4A, 10.8 Å) within the A′α–Aβ loop, while V416 (V416-CA···FMN-C4A, 8.2 Å) and Q513 (Q513-CA···FMN-C4A, 7.6 Å) are located within the FMN binding pocket interacting with the chromophore. Here, the side chain of V416 is found at the hydrophobic side of the flavin isoalloxazine ring, making van der Waals contacts with the flavin dimethyl-benzene moiety, likely also sterically impacting the conformation of the photoactive cysteine residues in the LOV domain.^44^ Q513, which is involved in the structural activation and signal-relay mechanism of LOV domains,^55,57^ but was very recently found not to be absolutely essential for signaling,^72^ is located at the polar side of the isoalloxazine ring forming an H-bond with the FMN-O4 atom in the dark state (Figure 3A). In contrast, the more proximal N414 side chain is deeply embedded in an H-bonding network with neighboring residues that include Q513 and D515, all of which also yield variants with altered dark-recovery kinetics when mutated (Figure 3A).

Interestingly, N414, which appeared as a mutational hot spot in our and previous studies, seems to be involved in the light-dependent signal-relay process in AsLOV2, as shown early on by X-ray crystallography, with the N414-D515 hydrogen bond being broken upon light-dependent adduct formation, mediating the displacement of N- and perhaps also C-terminal flanking elements (A′α- and Jα-helix) involved in photoreceptor activation.^55^ Recent molecular dynamics (MD) simulations have shed additional light on the role of N414 in the light-dependent signal relay in AsLOV2.^73^ Based on MD simulations, infrared spectroscopic data, NMR spectroscopy, and optogenetic experiments, the authors proposed that the hydrogen bond between the NH side chain of N414 and backbone C=O of Q513, which is present in the dark state, is broken upon illumination, and a new transient H-bond is formed between the NH and C=O side-chain groups of N414 and Q513, respectively.

This new interaction is facilitated by a lever-like motion of Q513 after Cys adduct formation, which in turn forces the Jα-helix away from the LOV domain, initiating helix unfolding and photoreceptor activation.^73^ In contrast, very recent high-resolution structural data for AsLOV2 suggested that the Q513 side chain undergoes a 180° flip (as suggested also in earlier reports), while N414 flips toward Q513, enabling a new hydrogen bond between the N414-OD1 atom and NE2 of Q513.^72^ Irrespective of the structural change N414 experiences upon illumination, its importance for the intramolecular signal relay is clear from these studies. Lastly, of the residues that show contrasting mutational effects, K485 located within the Gβ-Hβ loop, which forms a salt bridge with E444 on Dα (Figure 3B), appears to stabilize the conformation of the Dα-helix. Stabilization seems to occur via a network of H-bonds, where E444 forms a H-bond to the backbone nitrogen of S441 (E444-OE1···S441–N) (Figure 3B).

Interestingly, Dα also harbors I445 backbone H-bonding to S441 (S441–O···I445–N), which was recently shown to be involved in tuning the adduct-state lifetime of AsLOV2 and other LOV domains.^56^ Thus, the conformation of the Dα-helix and the hydrophobic I445 pocket seems instrumental for dark-recovery tuning.

The fastest literature-known variant was identified to be N449S (τ_FMN_ = 0.99 s,^43^), which interacts with the ribityl chain of FMN and is deeply imbedded in an H-bonding network that stabilizes the C450 containing Eα-helix (Figure 3C). In our experimental data set, we were only able to obtain the N449E variant in chromophore-loaded form, showing a slightly faster dark recovery than the wild type, while the corresponding A, I, K, and T variants did not bind flavin, indicating that this residue is important for FMN binding in AsLOV2. Other faster variants include variants at position I427, with the I427T mutation yielding the largest acceleration (Figure 2D). For AsLOV2, I427 was proposed to be in van der Waals contact with the sulfhydryl group of the photoactive cysteine (C450) (Figure 3D), likely providing steric support, and that mutations at this position destabilize the adduct state and hence accelerate the dark recovery.^43^

While the newly tested single-site variants did not reveal any new dark-recovery tuning mechanisms, since our ML prediction focused on known positions, the presented analysis nevertheless corroborates and strengthens known mechanistic proposals, comprehensively illustrating how single-site substitutions influence the dark recovery of LOV domains. At the same time, our comprehensive analyses also illustrate the biased nature of the current data set, where the most studied positions also show the largest variability in terms of τ_FMN_. Much more studies on site-directed variants would be needed, i.e., covering the complete sequence space of the protein by performing scanning saturation mutagenesis, to allow unbiased ML predictions and yield a clear picture of single-site mutational effects in LOV domains.

### ML-Guided Design Correctly Predicts Combinatorial Effects

Next, we will discuss mechanistic aspects and potential combinatorial effects that contribute to the observed acceleration or, respectively, slow-down. Here, we considered the effects of two or more variants to be combinatorial or additive when the combination of the independent mutations yielded a larger effect as observed for either of the single variants. Similarly, combinatorial effects were assumed to be present when multiple slow- and fast-reverting single mutants were combined, and the corresponding multiple mutants showed a recovery in between the extreme values of the corresponding single mutants. Table 1 compiles the “best” hits in terms of fast and slow cycling AsLOV2 variants, which were obtained within our three prediction–validation–prediction cycles.

**Table 1:** Selected Fast and Slow Recovering Variants^a^

round	variant	selection/prediction	τ_FMN_ (s)	*T*_m_ [°C]
n.a.	wild type	n.a.	43 ± 4.5^b^	52.5
1	K413C/N414C	predicted, slow	162 ± 1^e^	n.d.
1	N414C	selected, slow	961 ± 98	n.d.
1	K413C	selected slow	35 ± 4	n.d.
1	I427T/E475T	predicted, fast	0.5 ± 0.1	46.6
1	I427T	selected, fast	0.7 ± 0.1	48.1
1	E475T	selected fast	33 ± 1	52.2
2	N414L/V416L^c^	predicted slow	>103,020	60
2	N414L	selected slow	1641 ± 56	n.d.
n.a.	V416L^d^			n.a.
2	N414A/V416L	predicted slow	59,744 ± 8771	48.1
n.a.	N414A^c^			n.a.
n.a.	V416L^d^			n.a.
2	E475T/G528E	predicted, fast	22 ± 2	n.d.
1	E475T	selected fast	33 ± 1	n.d.
1	G528E	selected fast	18.8 ± 0.1	n.d.
3	I427T/L446M/E475T	predicted, fast	0.4 ± 0.01	44
1	I427T/E475T	predicted, fast	0.5 ± 0.1	46.6
1	I427T	selected, fast	0.7 ± 0.1	48.1
1	L446M	selected, slow	36 ± 2	54
1	E475T	selected, fast	33 ± 1	52.2

a For each prediction round, the fastest and slowest predicted combinatorial variants were selected (shown in bold). To rationalize the observed effect, the recovery data for the corresponding single-site variants that constitute the double or triple variant are shown, as well. The complete data set can be found in the Supporting Information (Tables S4–S8). *T_m_* corresponds to the melting temperature, determined by differential scanning fluorimetry (see Figure S20/S21).

b Data derived from three independent measurements of three independently purified samples. We considered a variant to show a recovery similar to the wild type when τ_FMN_ was between 34 and 52 s.

c Tends to aggregate.

d The N414A and V416L variants were not generated and tested as single variants in our study, but previous studies revealed a slow dark recovery of these variants with τ_FMN_ = 1427 s^49^ and τ_FMN_ = 4300 s,^51^ respectively; n.a. not applicable.

e τ_FMN,ave_ with τ_FMN,slow_ = 193.8 ± 5.8 s (*A*_slow_ = 79.3 ± 1.5%) and τ_FMN,fast_ = 28.0 ± 12.2 s (*A*_fast_ = 20.7 ± 1.5%) (see Table S13).

The slowest combinatorial variant obtained in the first prediction cycle was the K413C/N414C double variant, whose τ_FMN_ appears to result from a combination of acceleration by the K413C variant, and the N414C mutation, yielding a much larger deceleration when mutated independently (Table 1). Even though K413C shows within the error a τ_FMN_ similar to the wild type when studied as a single variant, the faster recovery of the K413C/N414C variant compared to the much slower N424C single variant, nevertheless points toward combinatorial effects. In contrast, the fastest first-round variant results from double mutation I427T/E475T (τ_FMN_ = 0.5 s). Here, the two independent mutations yield variants that are faster (I427T, τ_FMN_ = 0.7 s) or slower (E475T, τ_FMN_ = 33 s) compared to the double variant, and hence the observed fast recovery of I427T/E475T seems to be largely due to the I427T mutation. Second and third-round predictions yielded slower (N414L/V416L, τ_FMN_ > 103,020; N414A/V416L, τ_FMN_ = 59,744 s) and faster (I427T/L446M/E475T, τ_FMN_ = 0.4 s) variants compared to first-round predictions, respectively. Here again, the N414L/V416L and the N414A/V416L variants seem to result from combinatorial effects, since the respective single-site variants showed a slower recovery compared to the wild type (Table 1). Please note that both of the slow cycling variants identified here are prone to aggregation, which impacts data quality. Similar issues were observed for the previously reported slowest cycling AsLOV2 variant (N414V, τ_FMN_ > 43,200 s^58^), which also tended to aggregate.^58^ This, on the one hand, suggests that the observed aggregation likely results from the N414V mutation, while it unfortunately renders a direct comparison to literature data impossible.

The very fast recovery of the I427T/L446M/E475T variant, within the error of the measurement, seems to largely stem from the I427T mutation (τ_FMN_ = 0.7 s), and only minor contributions from E475T, which when mutated independently yield a slightly faster recovery (τ_FMN_ = 33 s, Table 1). The latter combinatorial effect is corroborated by the observation that the double mutant I427T/E475T shows a faster recovery (τ_FMN_ = 0.5 s, Table 1) compared to both single variants and is within the error of the measurement equally fast as the I427T/L446M/E475T variant. Thus, the additional L446M mutation does not seem to play a major role. Compared to literature data, the here identified fast cycling variants I427T/L446M/E475T show faster FMN-Cys adduct rupture as compared to the previously reported fastest variant (N449S, τ_FMN_ = 0.99 s^43^).

In addition, in order to assess the suitability of the fast and slow cycling variants identified here for *in vivo* (optogenetic) studies, we used differential scanning fluorimetry-based unfolding experiments to study the thermal stability of selected variants. Melting temperatures (*T*_m_) are shown in Table 1, and unfolding data is provided in Figure S20/S21. AsLOV2 wild type shows a melting temperature of *T*_m_ = 52.5 °C. Each mutation, with the exception of L446M, introduced into the fast cycling triple mutant I427T/L446M/E475T seems to slightly reduce the thermal stability. While the AsLOV2-E475T mutant is as thermostable as the wild type (*T*_m_ = 52.2 °C), the I427T variant shows a reduced melting temperature of *T*_m_ = 48.1 °C. A combination of I427T and E475T reduces the thermal stability of the corresponding double mutant (*T*_m_ = 46.6), while the triple mutant, in turn, is even less thermostable, showing a *T*_m_ = 44 °C. Similarly, the slow cycling N414A/V416L mutant shows a somewhat reduced thermal stability with a *T*_m_ = 48.1 °C, while the slowest N414L/V416L variant seems to be slightly more thermostable (*T*_m_ = 60 °C). Thus, while all the tested mutations that tune the recovery of AsLOV2 apparently had some impact on the thermal stability of the protein, the fastest and slowest identified variants should still be stable under moderate cultivation temperatures, e.g., when used in optogenetic experiments (see, e.g.,^74−76^) or when employed to generate plants with altered phototropin kinetics.^37^

To infer global effects and to visualize potential combinatorial networks, we mapped the results of multisite mutations as color-coded dashed lines connecting the constituting variant positions on the structure of AsLOV2 (Figure 3E,F). Blue, red, and orange dashed lines hereby represent slower and faster reverting variants or variants that show a recovery similar to the wild type, respectively. Multiple amino acid substitutions, yielding faster (Figure 3E) or slower (Figure 3F) dark recovery, when mutated together, were found located throughout the LOV domain. It is to be expected that some of the substitutions have a potential effect on each other when they occur simultaneously in close proximity. However, it is most likely that the substitutions individually alter the protein’s dark recovery. Interestingly, the majority of the connected mutations are located in distant regions outside the FMN binding pocket of AsLOV2. There is some overlap between structural regions containing faster and slower-reverting variants, with the corresponding mutations being mainly found on the A′α-Aβ loop, the Aβ strand, the Dα-helix, Iβ, and Hβ. Faster reverting multisite variants are more prevalent at the A′α-Aβ loop (T406), A′α-helix (T407 and L408), and the Cα-helix (D432, Q436, T438), while slower-reverting variants are more frequently located at the Iβ and Hβ strands (Q513, L515, D515, L496, and H495). Interestingly, key amino acid positions for tuning the dark recovery in both directions involve a combination of V416, N414, and Q513, which are part of an extended H-bonding network of residues connecting the FMN chromophore to N and C-terminal downstream signaling elements (A′α and Jα-helix) (see above), which themselves harbor residues that contribute to the tuning of the dark recovery. While it thus appears at first glance that signaling relevant positions and dark recovery tuning are somehow linked, we cannot rule out that this observation is also linked to the biased nature of the underlying data set.

For the best hit multisite variants (Table 1) obtained in our study, the following structure–function relationships seem to be at play. Substitution of N414 with leucine or cysteine disrupts or alters the N414-ND2···OD1-D515 side-chain-induced hydrogen-bonding network (Figure 3A) slowing down the recovery of the double variant, while the substitution of K413 can disrupt the K413···T535 hydrogen bond, which is exclusively present in the dark state as seen in recent high-resolution crystal structures of AsLOV2,^72^ although the K413 side chain showed multiple conformation with only one being in hydrogen-bonding distance (K413-NZ_A_···T535-OG1, 2.8 Å, PDB ID: 7PGX). This might result in a faster recovery, yielding intermediate τ_FMN_ for the corresponding double variant. The most pronounced deceleration is caused by the N414L/V416L double mutant, which apparently acts independently on the dark recovery of AsLOV2 by disrupting the N414-ND2···OD1-D515 hydrogen-bonding network. The stability of the FMN:protein adduct is hereby likely affected via changing the conformation of C450, with the V416L substitution hindering reversion of the C450 dark-state conformation (Figure 3A). In contrast, the substitution of I427 with threonine may possibly cause hydrogen bonding with residue R448, which is in close proximity to C450 and is likely to alter the binding of C450 to FMN and could thus affect the adduct-state lifetimes by altering the stability of the structural FMN binding mode (Figure 3D) (significantly faster FMN absorbance recovery of variant I427T: τ_FMN_ = 1 ± 0). Further, substituting residue E475 could cause a change in the interaction of the hydrogen bond network (E475···K533···Q497···H495···T477) that spans between the core LOV2 domain (residue positions 404–546) and the C-terminal helix (residue positions 521 to 544) and could affect the protein core stability.

### Limitations of the Study and Implications for the Design and Tuning of Optogenetic Tools

As outlined in the introduction, dark recovery, as a whole, is a complex, multistep process that cannot be captured by a single technique.^31−33^ As is the case for many protein engineering campaigns that rely on screening a larger number of variants, our study shows important limitations that have implications for the design of optogenetic tools based on the identified mutants. First, in this work, we only monitored the recovery of the dark-state flavin absorption by UV/Vis spectrophotometry as a proxy for the recovery of the photoreceptor, being well aware that photoreceptor dark recovery also involves recovery of the dark-state structure of the protein, which cannot be monitored by simple UV/Vis spectrophotometry. Second, our experimental approach does not capture if the obtained novel variants are indeed signaling competent, since FMN-Cys adduct formation is no direct proof of photoreceptor activation in a functional sense.^77^ Lastly, since here we only study the isolated AsLOV2 domain (including its signaling relevant A′α and Jα helices), we cannot fully rule out that the presence of other domains, e.g., as in full-length phototropin or designed optogenetic tools, has an impact on photoreceptor kinetics. In fact, several studies have shown that photoreceptor kinetics are indeed influenced by the nature of the associated effector domains or even short N- and C-terminal extensions.^27,28,78^ However, many studies have shown that mutations identified to impact the kinetics of isolated LOV domains, when introduced in the natural or designed full-length multidomain photoreceptors, at least produce similar effects, e.g., yielding faster or slower variants, respectively.^27,37,44,77^ The magnitude of the observed change might however be different. To address these aspects, either structural methods would be needed to monitor light-dependent structural changes for these variants or an AsLOV2-based optogenetic tool could be employed to verify functionality. Additionally, we wish to stress that gaining deeper insights into the effect of mutations on the structural dynamics of LOV domain variants and the dark recovery process would benefit from elaborate molecular dynamics simulations on both the dark- and light-adapted state of the variants.^79^ Such studies, however, are beyond the scope of this study, in which we explored the feasibility of ML-based methods to guide the rapid engineering of (combinatorial) LOV photoreceptor variants.

## Conclusions

Protein engineering based on a rational design approach is widely used to fine-tune the properties of photoactive proteins. However, LOV proteins with tailored dark recoveries are often challenging to obtain due to the lack of a comprehensive mechanistic model and a large protein sequence space. We conducted three rounds of mutagenesis, aiming at variants with slower and faster dark recovery, where an initial low-*N* pool of protein variants from literature was used to train ML models to guide mutagenesis for the combination of substitutions at reported literature positions next to the rational selection of single substitutions at these positions. After three rounds of ML-guided engineering, LOV domain variants were successfully obtained, whose adduct-state lifetimes spanned 7 orders of magnitude. Our ML study focuses on known positions and corroborates and strengthens known mechanistic proposals, comprehensively illustrating how single-site substitutions influence the dark recovery of LOV domains.

In terms of hit rate, for this low-*N* engineering task based on a limited body of literature data, machine learning achieved only mediocre hit rates in correctly predicting AsLOV2 variants with slower and faster dark recovery times. However, it nevertheless proved to be a viable strategy for uncovering variants with highly altered fitness, i.e., dark recovery times. These results demonstrate the potential of ML-assisted protein engineering as a powerful method for combining amino acid substitutions to evolve protein properties that are difficult to predict rationally.

## Supporting Information Available

Supporting Tables: Literature data for variants of AsLOV2 with altered dark-recovery kinetics; models for ML training and model validation; data for three rounds of ML prediction and experimental validation; bacterial strains, plasmids, and oligonucleotides used in this study; experimental parameters for measuring the dark recovery. Supporting Figures: Absorption spectra of AsLOV2 variants obtained over three prediction–validation cycles; dark-recovery times traces of AsLOV2 variants obtained over three prediction–validation cycles; dark-recovery data for aggregation-prone, slow reverting AsLOV2 variants; dark-recovery time traces for fast and slow reverting double and triple variants; thermal stability of selected slow and fast cycling AsLOV2 variants; SDS-PAGE analysis of AsLOV2 variants generated, purified, and characterized in this study. Supporting Text: Detailed information on the PyPEF framework; evaluation of ML-guided prediction of variants in each round (PDF)
