# Supplementary material for: Machine Learning-Assisted Engineering of Light, Oxygen, Voltage Photoreceptor Adduct Lifetime
Source: JACS Au. 2023 Nov 21;3(12):3311–23. doi: 10.1021/jacsau.3c00440 (PMC10751770; doi:10.1021/jacsau.3c00440)
Supplement: Supplementary file 1 — au3c00440_si_001.pdf [file au3c00440_si_001.pdf]

Supporting Information

Machine-learning-assisted engineering of light, oxygen, voltage photoreceptor adduct lifetime

Stefanie Hemmer[1,§], Niklas Erik Siedhoff[3,4,§], Sophia Werner[1], Gizem Ölçücü[1], Ulrich Schwaneberg[3,4,], Karl-Erich Jaeger[1,2], Mehdi D. Davari[5,*], Ulrich Krauss[1,2,6*]

[1]Institute of Molecular Enzyme Technology, Heinrich Heine University Düsseldorf, Wilhelm Johnen Strasse, Jülich 52426, Germany

[2]Institute of Bio-and Geosciences IBG 1: Biotechnology, Forschungszentrum Jülich GmbH, Wilhelm Johnen Strasse, Jülich 52426, Germany

[3]Institute of Biotechnology, RWTH Aachen University, Worringer Weg 3, 52074 Aachen, Germany

[4]DWI-Leibniz Institute for Interactive Materials, Forckenbeckstraße 50, 52074 Aachen, Germany

[5]Department of Bioorganic Chemistry, Leibniz Institute of Plant Biochemistry, Weinberg 3, 06120 Halle, Germany

[6]Department of Biochemistry, University of Bayreuth, 95447 Bayreuth, Germany.

§: contributed equally

* corresponding authors: MDD: Mehdi.Davari@ipb-halle.de

UK: u.krauss@fz-juelich.de

ulrich.krauss@uni-bayreuth.de

Table of contents

Figures/Table	Page
**Supporting Text S1:** Machine Learning-Guided Design using the PyPEF framework	**S4**
**Supporting Text S2:** Evaluation of ML guided prediction of variants in each round	**S5**
**Table S0:** Measuring conditions and buffers used in the different studies listed in Table S1.	**S6**
**Table S1:** Variant AsLOV2 data collected from literature and generated and screened AsLOV2 variants.	**S6**
**Table S2:** Selected models (amino acid descriptors used for encoding) for diverse data splits for training and model validation.	**S8**
**Table S3:** Third round ML predictions using the hybrid modeling approach.	**S10**
**Table S4:** Experimental validation of all first-round selections.	**S11**
**Table S5:** Experimental validation of all first-round ML predictions.	**S13**
**Table S6:** Experimental validation of all second-round selections.	**S15**
**Table S7:** Experimental validation of all second-round ML predictions.	**S16**
**Table S8:** Experimental validation of all third-round ML predictions.	**S17**
**Table S9:** Bacterial strains and plasmids used in this study.	**S18**
**Table S10:** Oligonucleotide primers used in this study. The exchanged codon is shown in bold red.	**S22**
**Table S11:** Summary of measurement duration (min), averaging time (sec) and cycle time (min) of dark recovery measurements of all measurable AsLOV2 variants.	**S26**
**Table S12:** Summary of measurement duration (min), time intervals (min) and number of measured dark recovery spectra of AsLOV2-N414A/V416A, -N414A/V416L, -N414L/V416L and -N414L/Q513A.	**S29**
**Table S13:** Recovery kinetic time constants for variants showing a two-phase decay.	**S29**
**Figure S1:** Absorption spectra of the dark and light states of first round selected fast single AsLOV2 variants.	**S30**
**Figure S2:** Absorption spectra of the dark and light states of first round selected slow single AsLOV2 variants.	**S31**
**Figure S3:** Absorption spectra of the dark and light states of first round ML-based predicted fast double AsLOV2 variants.	**S32**
**Figure S4:** Absorption spectra of the dark and light states of first round ML-based predicted slow double AsLOV2 variants.	**S33**
**Figure S5:** Absorption spectra of the dark and light states of second round selected single AsLOV2 variants.	**S34**
**Figure S6:** Absorption spectra of the dark and light states of second round ML-based predicted fast double AsLOV2 variants.	**S35**
**Figure S7:** Absorption spectra of the dark and light states of second round ML-based predicted slow double AsLOV2 variants.	**S36**

**Figure S8:** Absorption spectra of the dark and light states of third round ML-based predicted fast double and triple AsLOV2 variants.	**S37**
**Figure S9:** Absorption spectra of the dark and light states of third round ML-based predicted slow double and triple AsLOV2 variants.	**S38**
**Figure S10:** Dark recovery time traces of first round selected fast single AsLOV2 variants.	**S39**
**Figure S11:** Dark recovery time traces of first round selected slow single AsLOV2 variants.	**S40**
**Figure S12:** Dark recovery time traces of first round ML-based predicted fast double AsLOV2 variants.	**S41**
**Figure S13:** Dark recovery time traces of first round ML-based predicted slow double AsLOV2 variants.	**S42**
**Figure S14:** Dark recovery time traces of second round selected single AsLOV2 variants.	**S43**
**Figure S15:** Dark recovery time traces of second round ML-based predicted fast double AsLOV2 variants.	**S44**
**Figure S16:** Dark recovery time traces of second round ML-based predicted slow double AsLOV2 variants.	**S45**
**Figure S17:** UV/Vis spectra recorded during the dark recovery of a subset of aggregation-prone AsLOV2 variants or of variants showing a very slow recovery.	**S46**
**Figure S18:** Dark recovery time traces of third round ML-based predicted fast double and triple AsLOV2 variants.	**S47**
**Figure S19:** Dark recovery time traces of third round ML-based predicted slow double and triple AsLOV2 variants.	**S48**
**Figure S20:** Thermal stability of AsLOV2 wild type and selected slow cycling AsLOV2 variants.	**S49**
**Figure S21:** Thermal stability of AsLOV2 wild type and selected fast cycling AsLOV2 variants.	**S50**
**Figure S22-S29:** SDS-PAGE analysis of AsLOV2 variants generated, purified and characterized in this study	**S51**
**Supporting references**	**S59**

Supporting Text S1: Machine Learning-Guided Protein Design using the PyPEF Framework

ML predictions were made with PyPEF [15], a data-driven protein engineering framework. PyPEF utilizes amino acid index sets from the AAindex database [1] for sequence encoding. In addition, a fast Fourier transform is applied to the AAindex-encoded variant sequences, resulting in holistically modified variant vectors. PyPEF validates model performance by iterating over the 566 available AAindex physicochemical descriptor sets to find a suitable set to be used for prediction [2]. For each case, model training was performed in a supervised manner, computing the mean-squared error on training sets used for model fitting and iterative optimization by validation on the remaining training set entries to adjust model hyperparameters using using partial least-square (PLS) regression in combination with leave-one-out-based and k-fold grid-search methods available from the scikit-learn package [3]. Total data subsets (data splits) used for model training and model testing are described in Tables S2 and S3. In machine learning, dataset splits refer to how we divide our dataset into different subsets for the purposes of training, validation, and testing. By splitting our dataset into these subsets, we can assess and improve our machine learning model's performance while ensuring it generalizes well to new, unseen data. Using the training datasets, the number of components used in PLS regression modeling were optimized. As performance metrics, the coefficient of determination (R^2), the root-mean-squared error (RMSE), and the Spearman's rank correlation coefficient (ρ) on test datasets between observed and predicted fitness was considered. For the third round of predictions, a hybrid modeling approach termed MERGE [4], implemented in the PyPEF framework, based on encoded sequences from a direct-coupling analysis (DCA)-based approach on aligned homologous sequences was used [5, 6]. MERGE method combines evolutionary probability and machine learning to generate a hybrid model for data-driven protein engineering. The MERGE model is built based on a multiple sequence alignment to derive DCA-based features, train a (ridge) regression model, and combine and optimize the predictions of the linear model with the output of a statistical coevolution model [4, 5, 7]. Based on such evolutionary encoding and hybrid prediction, model performance is in general, as observed for many public engineering datasets, increased compared to amino acid-specific encodings and pure ML model test set predictions – especially when only low numbers ("low N") of variants-fitness pairs are available for training [4].

In the first round, the naturally possible diversity at each position was predicted, rather than limiting model predictions and lab-validation to the recombinations of the identified

substitutions, to potentially provide a larger set of individual substituted variants for further recombination. Further, identified positions from the literature allowed an efficient rational selection of amino acid exchanges by site-directed mutagenesis (SDM) at specific positions to enlarge the sequence-fitness library for model construction and for lowering the bias of data derived from different screening assays used for model training and prediction. Incorporating the SDM data into the literature data, as well as the predicted and tested double-substituted variants from the first round, we performed a second and similarly a third round of predictions of recombinants and lab validation, applying different models that learned on specific content or all content of the collected data (see Tables S2 and S3).

Supporting Text S2: Evaluation of ML-guided prediction of variants in each round

In the first round, modeling the effects of the potential diversity of amino acid substitutions at the reported positions proved to be less successful, i.e., the hit rate was low, especially as many predicted variants did not express or did not bind flavin. This is to be expected to some extent, as models were not provided with information about substitutions reducing expression or flavin binding. Here, evolution-based encoding techniques may provide a suitable solution for predicting the effects of amino acid substitutions in the wider variety of chemical and positional manifestations at lower risk of failed expression and flavin binding [8]. Noteworthy, possibly due to the added site-directed mutagenesis (SDM) data and the restriction of predictions to recombinants, the second-round predictions ensured higher success in expression and flavin binding (all variants could be expressed and measured) and showed significantly improved hit rates in predicting variants with slower dark recovery times. Besides, for the prediction of variants with fast recovery times, four variants showed recovery times that were similar to the wild type and three variants that were found to be incorrectly predicted resulted in recovery times of ~ 60 s. Considering the low performance and the small amount of variant fitness data available for model validation, the results of the second round can be interpreted as positive. In addition, predictions of variants in the third round using an evolution-based encoding and hybrid modeling technique resulted in a triple-substituted variant with a short dark recovery time of 0.4 s.

Literature dataset and summary of experimental data used for ML-model training

Please note that in the different studies somewhat different experimental conditions were used. Most importantly, buffer composition, pH and measuring temperatures varied (Table S0).

Table S0: Measuring conditions and buffers used in the different studies listed in Table S1. The No. given in the table corresponds to the study listed in Table S1.

No.	Buffer	Temperature [°C]
1 (this study)	50 mM Tris, 1 mM EDTA and 5 mM DTT, pH 8.0	25
1.1	50 mM Tris, 1 mM EDTA and 5 mM DTT, pH 8.0	22
1.2	50 mM Tris, 1 mM EDTA and 5 mM DTT, pH 8.0	22
1.3	50 mM Tris, 1 mM EDTA and 5 mM DTT, pH 8.0	22
1.4	50 mM Hepes, 10 % Glycerol, 150 mM NaCl, pH 8.0	not given
1.5	50 mM Tris HCl and 150 mM NaCl, pH 7.5	25
1.6	20 mM Tris and 150 mNaCl, pH 8.0	20
1.7	50 mM sodium phosphate buffer,100 mM NaCl, pH 6.0	22
1.8	20 mM sodium phosphate buffer, 10 mM NaCl, 1 mM DTT, 10.53 % (v/v) D_2O, pH 6.4	25

Table S1: Variant AsLOV2 data collected from the literature and generated and screened AsLOV2 variants. White row color indicates literature variants, grey row color indicates variants screened/validated after first round of selection and prediction and dark grey color and darkest gray color after second and third round of screening/validation, respectively. Asterisk (*) indicates variants that were screened/validated after model training for next round of evolution and thus could not be used for model training and prediction. Measuring conditions given in the literature are listed in Table S0 and can be identified by No. and Reference.

No.	Variant	τ_{FMN} (s)	Reference
**1**	WT	43 ± 4,5	this work
**1.1**	WT	81	[9]
**1.2**	WT	80	[10]
**1.3**	WT	81	[11]
**1.4**	WT	81	[12]
**1.5**	WT	55	[13]
**1.6**	WT	37	[14]
**1.7**	WT	60	[15]
**1.8**	WT	49	[16]
**2**	I445T	152	[16]
**3**	T407P	81	[9]
**4**	T407W	82	
**5**	L408D	96	
**6**	R410P	108	
**7**	R410G	78	
**8**	K413A	59	
**9**	N414V	43200	
**10**	F434L	12	
**11**	E475A	67	
**12**	Q479L	49	
**13**	N492A	54	
**14**	L493A	121	
**15**	F494L	206	
**16**	H495L	85	
**17**	Q497A	74	
**18**	R500A	57	
**19**	Q502A	78	
**20**	Q513A	261	
**21**	L514A	83	
**22**	D515V	54	
**23**	H519A	80	
**24**	E409P	98	[10]
**25**	N414T	892	
**26**	F415E	65	
**27**	V416A	22	
**28**	T418V	56	
**29**	D419A	77	
**30**	R421G	82	
**31**	R421D	58	
**32**	D424A	194	
**33**	I427A	6	
**34**	I427V	6.5	
**35**	I428M	52	
**36**	I428T	53	
**37**	I428V	72	
**38**	F429Y	66	
**39**	S431A	65	
**40**	D432A	54	
**41**	S433L	48	
**42**	S433V	64	
**43**	L435A	263	
**44**	T438V	32	
**45**	E439A	71	
**46**	R442L	121	
**47**	E443A	58	
**48**	E444A	75	
**49**	I445A	336	
**50**	L446E	42	
**51**	F452L	300	
**52**	R460A	78	
**53**	T462V	57	
**54**	R467A	96	
**55**	A469G	40	
**56**	D471V	68	

**57**	V476F	63	
**58**	T477V	71	
**59**	L480A	34	
**60**	T484V	62	
**61**	K485A	93	
**62**	K485L	62	
**63**	F490L	47	
**64**	W491Y	85	
**65**	W491K	79	
**66**	L496F	84	
**67**	M499A	81	
**68**	D501G	80	
**69**	F509Y	66	
**70**	F509L	181	
**71**	D515L	56	
**72**	T517V	76	
**73**	G528V	103	
**74**	G528A	38	
**75**	E541K	87	
**76**	N414S	685	
**77**	N414A	1427	[11]
**78**	N414D	69	
**79**	N414G	615	
**80**	N414L	1847	
**81**	N414Q	280	
**82**	L453V	160	
**83**	Q513D	5	
**84**	Q513H	30	
**85**	Q513L	1436.5	
**86**	F494C	282	
**87**	V416I	821	[12]
**88**	V416T	2.4	[13]
**89**	V416L	4300	
**90**	I427T	1.99	[14]
**91**	K413R	40	
**92**	N449S	0.99	
**93**	Q513N	37.3	[15]
**94**	W491Y/L408W	78	[17]
**95**	T406A/T407A	56	
**96**	E412H/Q436H	39	
**97**	D432A/Q436A	73	[10]
**98**	S441A/E443A	85	
**99**	N414A/Q513H	2	[11]
**100**	N414L/Q513A	1900	
**101**	N414A/Q513A	2081	
**102**	C450V/F494C	69	
**103**	C450V/Q513C	44	
**104**	V416I/L496I	1009	[12]
**105**	K413R/I427V	5	[14]
**106**	K413R/I427L	19	
**107**	N414G/C450V/Q513C	13	[10]
**108**	D432A/Q436A/E443A	76	
**109**	D432A/Q436A/T406A/T407A	62	
**110**	S441A/E443A/T406A/T407A	70	
**111**	E475T	32.8	this work
**112**	G528E	18.8	this work
**113**	G528K	36.6	this work
**114**	G528R	21.2	this work
**115**	I427N	7.1	this work
**116**	I427T	0.7	this work
**117**	N449E	19.7	this work
**118**	Q513H	54	this work
**119**	Q513P	420	this work
**120**	Q513R*	84	this work
**121**	V416T	1.64	this work
**122**	D501W	37.2	this work
**123**	D501Y	36.5	this work
**124**	K413C	35	this work
**125**	L446M	36	this work
**126**	L446Q	45.6	this work
**127**	L446S	59	this work
**128**	N414C	961	this work
**129**	N414E	196	this work
**130**	N414M	650	this work
**131**	T406C	45	this work
**132**	T406V	43.2	this work
**133**	I427T/E475T	0.5	this work
**134**	Q513R/G528K*	61	this work
**135**	Q513R/G528R*	64	this work
**136**	K413C/N414C	162	this work
**137**	L446M/D501W	29.3	this work
**138**	L446Q/D501Y	15.4	this work
**139**	L446S/D501Y	6.5	this work
**140**	N414G	217.4	this work
**141**	D515L	40.2	this work
**142**	D501G	41.3	this work
**143**	R410P	58.4	this work
**144**	K413A	27.7	this work
**145**	N414L	1641	this work
**146**	E475T/G528E	22	this work
**147**	N414G/G528E	242	this work
**148**	R410P/G528A	61	this work
**149**	R410P/G528E	59	this work
**150**	R410P/G528R	43.8	this work
**151**	K413A/N414G	456	this work
**152**	N414G/L446E	45	this work
**153**	N414G/L446S	527	this work
**154**	R442L/D515L	61	this work
**155**	K413A/D501G	40	this work
**156**	D501G/G528E	46	this work
**157**	N414A/V416A	48	this work
**158**	N414A/V416L	59744	this work
**159**	N414L/V416L*	> 103020	this work
**160**	N414L/L514A	968	this work
**161**	N414L/H495L	446	this work
**162**	N414L/Q513L	4507	this work
**163**	T438V/E475T	17.7	this work
**164**	V463W/E475T	43.6	this work
**165**	N414D/V416T	9.6	this work
**166**	I427T/L446M/E475T	0.40	this work
**167**	N414D/F415E/V416T	17.2	this work

Table S2: Selected models (amino acid descriptors used for encoding) for diverse data splits for training and model validation. Variant indices refer to variant numbering in Table S1. Blue colored variants (round 1) did not express or did not show dark recovery. Grey colored variants (round 1 and round 2) were not generated (not selected from predictions; for round 1 as they were repetitive). In round 1, models were finally trained on all literature variants and diverse amino acid combinations were predicted. In round 2, models were not finally trained on all (literature and round 1) variants and only recombinant amino acid substitutions were predicted. Green-, red-, and orange-colored variants were expressed successfully and could be screened: green colored variants were predicted correctly (estimated slow and fast dark recovery times were validated having dark recovery times $\tau_{\mathrm{FMN}}^{slow} > \tau_{\mathrm{FMN}}^{WT} + 3\,\sigma$ and $\tau_{\mathrm{FMN}}^{fast} < \tau_{\mathrm{FMN}}^{WT} - 3\,\sigma$, respectively) while red colored variants indicate wrong predictions, orange-colored variants were within $\tau_{\mathrm{FMN}}^{WT} \pm 3\,\sigma$.

Round	Learning set indices	Test set indices	Target label	Selected model (AAindex encoding)	Model performances						Aim	Predictions (variant ranking)			
					R^2	RMSE	ρ	R^2 CV$_5$	RMSE CV$_5$	ρ CV$_5$		1	2	3	4
1	1 – 93	94 – 106	τ_{FMN}	VELV850101	0.193	644.675	0.445	- 0.038	4269.999	0.036	Slow (diverse)	L446Q/D501Y	L446S/D501Y	L446C/D501Y	L446M/D501W
	1 – 93	94 – 106	τ_{FMN}^{-1}	ZIMJ680103	0.218	0.105	0.407	- 2.25e+21	5.9e+9	- 0.060	Fast (diverse)	Q513R/G528R	Q513R/G528E	Q513H/G528H	Q513R/G528K
	1 – 93	94 – 110	τ_{FMN}	GEIM800101	0.131	602.769	0.267	- 0.029	4174.227	0.189	Slow (diverse)	N414M/V416M	N414E/V416M	N414M/V416E	N414E/V416E
	1 – 93	94 – 110	τ_{FMN}^{-1}	ZIMJ680103	0.219	0.101	0.279	- 1.2e+21	4.3 e+9	- 0.045	Fast (diverse)	Q513R/G528R	Q513R/G528E	Q513H/G528H	Q513R/G528K
	1 – 110	1 – 110	τ_{FMN}	PONP800102	-	-	-	- 0.234	70.311	0.125	Slow (diverse)	T406C/N414C	T406V/N414C	K413C/N414C	N414C/L496K
	1 – 110	1 – 110	τ_{FMN}^{-1}	CHOP780212	-	-	-	- 0.171	0.125	0.277	Fast (diverse)	I427N/N449I	I427N/N449E	I427N/N449K	I427N/N449A
	1 – 110	1 – 110	τ_{FMN}^{-1}	AURR980120	-	-	-	0.115	0.116	0.266	Fast (diverse)	I427T/N449T	V416T/N449T	N449T/Q513P	I427T/E475T
2	1 – 93, 111 – 132	94 – 110, 133 – 139 (not *)	τ_{FMN}	PALJ810101	0.156	13.040	0.102	- 0.017	91.200	0.110	Slow (recombinat.)	N414E/D424A	N414E/V416L	N414E/V416A	T406V/N414E
											Fast (recombinat.)	E475T/G528E	N414G/G528E	N449E /G528E	S431A/G528E
	1 – 93, 111 – 132	94 – 106, 133 – 139 (not *)	τ_{FMN}	AURR980110	0.292	13.000	0.160	- 0.016	92.484	0.192	Slow (recombinat.)	N414A/V416L	N414M/V416A	N414A/V416A	N414E/V416L
											Fast (recombinat.)	R410P/G528A	R410P/G528E	R410P/G528R	R410P/N414A

	111 – 132	133 – 139 (not *)	τ_{FMN}	BULH740102	0.893	0.516	0.1	- 0.106	6.005	0.406	Slow (recombinat.)	N414L/L514A	N414L/Q513L	N414L/ I445T	N414L/ D424A
											Fast (recombinat.)	V416T /I427N	T406V /I427N	T406V /G528V	T406V /G528K
	1 – 139 (not *)	1 – 139 (not *)	τ_{FMN}	PTIO830102	(0.595)	(57.518)	(0.143)	0.462	66.334	0.120	Slow (recombinat.)	N414V/I427N	N414V/D515V	N414V/I427A	T406V /N414V
											Fast (recombinat.)	K413A /N414G	N414G /L446E	N414G/L446S	R410P/N414G
	1 – 8, 10 – 139 (not *)	1 – 8, 10 – 139 (not*)	τ_{FMN}	ARGP820102	(0.85)	(4.641)	(0.493)	0.728	6.406	0.404	Slow (recombinat.)	N414L/V416L*	V416L/N414M	V416L /L480A	N414C /V416L
											Fast (recombinat.)	V416T/ Q513D	R442L/D515L	K413C /V416T	V416T /Q513N
	111 – 139 (not *)	1 – 110	τ_{FMN}	NAKH900111	0.077	96.414	0.311	- 0.358	105.381	0.165	Slow (recombinat.)	N414L /H495L	N414L/Q513L	N414L /Q513A	N414L /Q479L
											Fast (recombinat.)	K413A/D501G	D501G/G528E	E444A/D501G	K413A/E444A

Table S3: Third round ML predictions using the hybrid modeling approach. The hybrid model parameters were adjusted on all the single substitution data and wild type (from literature and rounds one and two, indices 1 - 93) and performance was tested on all higher substituted variants (Spearman's $\rho = 0.314$). The trained and adjusted hybrid model was inferred for predicting (I) recombinants, (II) positional recombinants with natural amino acid diversity, and (III) double substitutions with one substitution being located at a new position or at a position that is not included in the single substituted variant data. The MSA was constructed on an alignment of 1976 homologous sequences. Blue colored variants did not express or did not show dark recovery. Grey colored variants were not generated (not selected from predictions). Green-, red-, and orange-colored variants were expressed successfully and could be screened: green colored variants were predicted correctly (estimated slow and fast dark recovery times were validated having dark recovery times $\tau_{\mathrm{FMN}}^{slow} > \tau_{\mathrm{FMN}}^{WT} + 3\,\sigma$ and $\tau_{\mathrm{FMN}}^{fast} < \tau_{\mathrm{FMN}}^{WT} - 3\,\sigma$, respectively) while red colored variants indicate wrong predictions, orange-colored variants were within $\tau_{\mathrm{FMN}}^{WT} \pm 3\,\sigma$.

		Predicted variant rank									
**Combination**	**Aim**	**1**	**2**	**3**	**4**	**5**	**6**	**7**	**8**	**9**	**10**
I: Recom-binant Doubles	Fast	L446M/ E475T	T438V/ E475T	L446M/ E475A	I427A/ E475T	I427T/ E475T	L446E/ E475T	T438V/ E475A	L446Q/ E475T	E475T/ L480A	I427T/ E475A
	Slow	N414D/ V416A	N414M/ V416A	N414C/ V416A	N414D/ V416L	N414M/ V416L	N414C/ V416L	N414D/ V416T	N414M /V416T	N414C/ V416T	N414M/ F415E
I: Recom-binant Triples	Fast	T438V/ L446M/ E475T	I427A/ L446M/ E475T	I427T/ L446M/ E475T	T438V/ L446M/ E475A	L446M/ E475T/ L480A	I427A/ T438V/ E475T	I427A/ L446M/ E475A	I427T/ L446M/ E475A	T438V/ L446E/ E475T	L446M/ T406V/ E475T
	Slow	N414D/ F415E/ V416A	N414C/ F415E/ V416A	N414M/ F415E/ V416A	N414D/ F415E/ V416L	N414C/ F415E/ V416L	N414M/ F415E/ V416L	N414D/ F415E/ V416T	N414C/ F415E/ V416T	N414M/ F415E/ V416T	N414D/ V416A/ R421G
II: Doubles with diverse amino acids	Fast	L446F/ E475C	L446F/ E475F	L446F/ E475I	L446F/ E475L	L446F/ E475M	L446F/ E475P	L446F/ E475T	L446F/ E475W	L446F/ E475Y	L446W/ E475C
	Slow	I427G/ L435Y	I427G/ T462R	I427G/ Q436D	I427G/ Q436P	I427G/ Q436T	I427G/ L453E	I427G/ F494T	I427G/ T477W	I427G/ T477Q	I427G/ I445H
III: Doubles with single new position	Fast	V463W/ E475C	V463W/ E475F	V463W/ E475I	V463W/ E475L	V463W/ E475M	V463W/ E475P	V463W/ E475T	V463W/ E475W	V463W/ E475Y	V463M/ E475C
	Slow	I427G/ L437D	I427G/ L437A	I427G/ L437A	I427G/ L437C	I427G/ L437E	I427G/ L437F	I427G/ L437G	I427G/ L437I	I427G/ L437L	I427G/ L437M

Table S4: Experimental validation of all first-round selections. Shown are the selection, flavin loading (%), recovery kinetics ($\tau_{FMN}(s)$) and if the experimental result matches its selection (color coded in column τ_{FMN}: green: matching experimental outcome and prediction, red: experiment and prediction did not match). Please note that the "selection" presented in the column "recovery selection" refers to the ML prediction whether a variant was predicted to be fast or slow. Only combinatorial variants were predicted by the ML model, single variant "predictions" were inferred from the corresponding prediction for the double variant assuming cooperativity. Adduct state lifetimes τ_{FMN} are reported as means of three technical replicates with the error representing the standard deviation of the mean. Faster and slower variants were selected based on a benchmarking dataset for three independently prepared and measured AsLOV2 wild type samples. A selected variant was considered faster or slower than AsLOV2 wildtype, if its adduct state lifetime is at least two standard deviations faster (<34 sec) or slower (>52 sec) than the wildtype (WT). Like WT: Selected variant is like WT (34-52 sec). —: No chromophore loaded. *: Tends to aggregate. n.a. Not applicable. $^{\%}$: in case a two phase exponential appeared better suited to fit the data (judged from residuals; see Materials and Methods section) τ_{FMN} is presented as average value according to $\tau_{FMN} = \sum(A_i\tau_i) / 100$.

**Fast**							
**AsLOV2 variant**	**Recovery selection**	**Concentration [mg/ml]**	**FMN loading [%]**	**One phase or two phase exponential fit**	**τ_{FMN} [s]$^{\%}$**	**Molecular weight [Da]**	**Extinction coefficient [1/M*cm]**
WT	n.a.	3.00	39.6	one	43 ± 4.5	18792.20	9970
E475T	fast	0.84	69.0	one	32.8 ± 0.4	18764.19	9970
G528E	fast	2.14	43.5	one	18.8 ± 0.1	18864.27	9970
G528H	fast	n.a.	n.a.	n.a.	could not be generated	n.a.	n.a.
G528K	fast	2.78	36.1	one	36.6 ± 0.4	18863.33	9970
G528R	fast	3.51	47.4	one	21.2 ± 0.6	18891.34	9970
I427N	fast	1.80	5.4	one	7.1 ± 1.3	18793.15	9970
I427T	fast	0.60	26.1	one	0.7 ± 0.1	18780.15	9970
N449A	fast	1.82	—	n.a.	—	18749.18	9970
N449E	fast	1.47	4.7	one	19.7 ± 5.2	18807.22	9970
N449I	fast	2.09	—	n.a.	—	18791.26	9970
N449K	fast	1.83	—	n.a.	—	18806.27	9970
N449T	fast	0.49	—	n.a.	—	18779.20	9970
Q513H	fast	7.54	12.3	one	54 ± 2	18801.21	9970
Q513P	fast	0.4	23.9	one	420 ± 8	18761.19	9970
Q513R*	fast	4.85	38.9	one	84 ± 24	18820.26	9970
V416T	fast	0.81	70.5	one	1.64 ± 0.01	18794.18	9970
**Slow**							
**AsLOV2 variant**	**Recovery selection**	**Concentration [mg/ml]**	**FMN loading [%]**	**One phase or two phase exponential fit**	**τ_{FMN} [sec]$^{\%}$**	**Molecular weight [Da]**	**Extinction coefficient [1/M*cm]**
WT	—	3.00	39.6	one	43 ± 4.5	18792.20	9970
D501W	slow	1.59	29.7	one	37.2 ± 0.2	18863.33	15470

Table S4 - continued

AsLOV2 variant	Recovery selection	Concentration [mg/ml]	FMN loading [%]	One phase or two phase exponential fit	τ_{FMN} [s] [%]	Molecular weight [Da]	Extinction coefficient [1/M*cm]
D501Y	slow	0.74	71.2	one	36.5 ± 2.4	18840.29	11460
K413C	slow	3.59	45.4	one	35 ± 4	18767.17	9970
L446C	slow	n.a.	n.a.	n.a.	could not be generated	n.a.	n.a.
L446M	slow	3.23	38.0	one	36 ± 2	18810.24	9970
L446Q	slow	1.68	22.4	one	45.6 ± 0.4	18807.17	9970
L446S	slow	1.45	16.8	one	59.0 ± 0.4	18766.12	9970
L496K	slow	1.50	—	n.a.	—	18807.22	9970
N414C*	slow	1.4	50.2	one	961 ± 98	18781.24	9970
N414E	slow	1.28	43.8	**two**	196 ± 5	18807.22	9970
N414M	slow	2.19	5.6	**two**	650 ± 24	18809.29	9970
T406C	slow	2.57	36.1	one	45 ± 1	18794.24	9970
T406V	slow	4.95	46.9	one	43.2 ± 0.2	18790.23	9970
V416E	slow	2.21	—	n.a.	—	18822.19	9970
V416M	slow	2.15	—	n.a.	—	18824.26	9970

Table S5: Experimental validation of all first-round ML predictions. Shown are the prediction, flavin loading (%), recovery kinetics ($\tau_{FMN}(s)$) and if the experimental result matches its prediction (color coded in column τ_{FMN}: green: matching experimental outcome and prediction, red: experiment and prediction did not match). Adduct state lifetimes τ_{FMN} are reported as means of three technical replicates with the error representing the standard deviation of the mean. Faster and slower variants were selected based on a benchmarking dataset for three independently prepared and measured AsLOV2 wild type samples. A predicted variant was considered faster or slower than AsLOV2 wildtype, if its adduct state lifetime is at least two standard deviations faster (<34 sec) or slower (>52 sec) than the wildtype (WT). Like WT: Predicted variant is like WT (34-52 sec). —: No chromophore loaded. *: Tends to aggregate. n.a. Not applicable. %: in case a two phase exponential appeared better suited to fit the data (judged from residuals; see Materials and Methods section) τ_{FMN} is preseted as average value according to $\tau_{FMN} = \sum(A_i\tau_i) / 100$.

Fast							
**AsLOV2 variant**	**Recovery prediction**	**Concentration [mg/ml]**	**FMN loading [%]**	**One phase or two phase exponential fit**	**τ_{FMN} [s] %**	**Molecular weight [Da]**	**Extinction coefficient [1/M*cm]**
WT	n.a.	3.00	39.6	one	43 ± 4.5	18792.20	9970
I427N/N449A	fast	1.15	—	n.a.	—	18750.12	9970
I427N/N449E	fast	1.75	—	n.a.	—	18808.16	9970
I427N/N449I	fast	1.18	—	n.a.	—	18792.20	9970
I427N/N449K	fast	0.94	—	n.a.	—	18807.22	9970
I427T/E475T	fast	1.38	13.5	one	0.5 ± 0.1	18752.14	9970
I427T/N449T	fast	0.88	—	n.a.	—	18767.15	9970
N449T/Q513P	fast	0.98	—	n.a.	—	18748.19	9970
Q513H/G528H	fast	n.a.	n.a.	n.a.	could not be generated	n.a.	n.a.
Q513R/G528E	fast	n.a.	n.a.	n.a.	could not be generated	n.a.	n.a.
Q513R/G528K*	fast	6.1	31.1	one	61 ± 7	18891.38	9970
Q513R/G528R*	fast	2.47	22	one	64 ± 1	18919.40	9970
V416T/N449T	fast	1.87	—		—	18781.18	9970
**Slow**							
**AsLOV2 variant**	**Recovery prediction**	**Concentration [mg/ml]**	**FMN loading [%]**	**One phase or two phase exponential fit**	**τ_{FMN} [s] %**	**Molecular weight [Da]**	**Extinction coefficient [1/M*cm]**
WT	—	3.00	39.6	one	43 ± 4.5	18792.20	9970
K413C/N414C	slow	5.25	40.0	**two**	162 ± 1	18756.20	9970
L446C/D501Y	slow	n.a.	n.a.	n.a.	could not be generated	n.a.	n.a.
L446M/D501W	slow	0.72	52.9	one	29.3 ± 0.7	18881.36	15470
L446Q/D501Y	slow	1.17	10.3	one	15.4 ± 1.3	18855.26	11460
L446S/D501Y	slow	2.36	10.7	one	6.5 ± 0.4	18814.21	11460
N414C/L496K	slow	1.42	—	n.a.	—	18796.25	9970
N414E/V416E	slow	2.31	—	n.a.	—	18837.20	9970
N414E/V416M	slow	1.92	—	n.a.	—	18839.28	9970

Table S5 - continued

AsLOV2 variant	Recovery prediction	Concentration [mg/ml]	FMN loading [%]	One phase or two phase exponential fit	τ_{FMN} [s]$^{\%}$	Molecular weight [Da]	Extinction coefficient [1/M*cm]
N414M/V416E	slow	1.71	—	n.a.	—	18839.28	9970
N414M/V416M	slow	1.71	—	n.a.	—	18841.35	9970
T406C/N414C	slow	n.a.	n.a.	n.a.	could not be generated	n.a.	n.a.
T406V/N414C	slow	n.a.	n.a.	n.a.	could not be generated	n.a.	n.a.

Table S6: Experimental validation of all second-round selections. Shown are the selection, flavin loading (%), recovery kinetics ($\tau_{FMN}(s)$) and if the experimental result matches its selection (color coded in column τ_{FMN}: green: matching experimental outcome and prediction, red: experiment and prediction did not match). Please note that the "selection" presented in the column "recovery selection" refers to the ML prediction whether a variant was predicted to be fast or slow. Only combinatorial variants were predicted by the ML model, single variant "predictions" were inferred from the corresponding prediction for the double variant assuming cooperativity. Adduct state lifetimes τ_{FMN} are reported as means of three technical replicates with the error representing the standard deviation of the mean. Faster and slower variants were selected based on a benchmarking dataset for three independently prepared and measured AsLOV2 wild type samples. A selected variant was considered faster or slower than AsLOV2 wildtype, if its adduct state lifetime is at least two standard deviations faster (<34 sec) or slower (>52 sec) than the wildtype (WT). Like WT: Selected variant is like WT (34-52 sec). No: Selection/ prediction is wrong. *: Tends to aggregate. n.a. Not applicable. [%]: in case a two phase exponential appeared better suited to fit the data (judged from residuals; see Materials and Methods section) τ_{FMN} is preseted as average value according to $\tau_{FMN} = \sum(A_i\tau_i) / 100$.

**Fast**							
**AsLOV2 variant**	**Recovery selection**	**Concentration [mg/ml]**	**FMN loading [%]**	**One phase or two phase exponential fit**	**τ_{FMN} [s]%**	**Molecular weight [Da]**	**Extinction coefficient [1/M*cm]**
WT	—	3.00	39.6	one	43 ± 4.5	18792.20	9970
N414G	fast	4.16	43.5	**two**	217.4 ± 4.8	18735.15	9970
D515L	fast	3.59	36.4	one	40.2 ± 2.4	18790.27	9970
D501G	fast	1.37	61.8	one	41.3 ± 0.5	18734.17	9970
R410P	fast	7.23	25.3	one	58.4 ± 2.2	18733.13	9970
K413A	fast	4.78	29.3	one	27.7 ± 1.7	18735.11	9970
**Slow**							
**AsLOV2 variant**	**Recovery selection**	**Concentration [mg/ml]**	**FMN loading [%]**	**One phase or two phase exponential fit**	**τ_{FMN} [s] %**	**Molecular weight [Da]**	**Extinction coefficient [1/M*cm]**
WT	—	3.00	39.6	one	43 ± 4.5	18792.20	9970
N414L	slow	2.57	34.6	one	1641 ± 56	18791.26	9970

Table S7: Experimental validation of all second-round ML predictions. Shown are the prediction, flavin loading (%), recovery kinetics ($\tau_{FMN}(sec)$) and if the experimental result matches its prediction (color coded in column τ_{FMN}: green: matching experimental outcome and prediction, red: experiment and prediction did not match). Adduct state lifetimes τ_{FMN} are reported as means of three technical replicates with the error representing the standard deviation of the mean. Faster and slower variants were selected based on a benchmarking dataset for three independently prepared and measured AsLOV2 wild type samples. A predicted variant was considered faster or slower than AsLOV2 wildtype, if its adduct state lifetime is at least two standard deviations faster (<34 sec) or slower (>52 sec) than the wildtype (WT). Like WT: Predicted variant is like WT (34-52 sec). No: Selection/ prediction is wrong. *: Tends to aggregate. n.a. Not applicable. %: in case a two phase exponential appeared better suited to fit the data (judged from residuals; see Materials and Methods section) τ_{FMN} is preseted as average value according to $\tau_{FMN} = \sum(A_i\tau_i) / 100$.

Fast							
**AsLOV2 variant**	**Recovery prediction**	**Concentration [mg/ml]**	**FMN loading [%]**	**One phase or two phase exponential fit**	**τ_{FMN} [s]**	**Molecular weight [Da]**	**Extinction coefficient [1/M*cm]**
WT	n.a.	3.00	39.6	one	43 ± 4.5	18792.20	9970
E475T/G528E	fast	3.41	32.8	one	22 ± 2	18836.26	9970
N414G/G528E	fast	3.89	30.2	**two**	242 ± 3	18807.22	9970
R410P/G528A	fast	7.2	19.8	**two**	61 ± 3	18747.16	9970
R410P/G528E	fast	4.6	29.4	**two**	59 ± 1	18805.2	9970
R410P/G528R	fast	2.6	31.7	one	43.8 ± 0.5	18832.27	9970
K413A/N414G	fast	5.01	32.5	**two**	456 ± 19	18710.12	10095
N414G/L446E	fast	4.81	27.8	one	45 ± 1	18751.11	9970
N414G/L446S	fast	5.68	32.3	**two**	527 ± 8	18709.07	9970
R442L/D515L	fast	3.38	29.1	one	61 ± 3	18747.25	9970
K413A/D501G	fast	2.67	28.6	one	40 ± 4	18677.07	9970
D501G/G528E	fast	3.96	30.6	one	46 ± 1	18806.23	9970
**Slow**							
**AsLOV2 variant**	**Recovery prediction**	**Concentration [mg/ml]**	**FMN loading [%]**	**One phase or two phase exponential fit**	**τ_{FMN} [s]%**	**Molecular weight [Da]**	**Extinction coefficient [1/M*cm]**
WT	—	3.00	39.6	one	43 ± 4.5	18792.20	9970
N414A/V416A*	slow	4.80	21.0	one	48 ± 3	18721.12	9970
N414A/V416L*	slow	4.64	17.6	one	59744 ± 8771	18763.21	9970
N414L/V416L*	slow	2.61	2.2	one	> 103020	18805.29	9970
N414L/L514A	slow	1.76	65.5	one	968 ± 42	18749.18	9970
N414L/H495L	slow	2.92	23.6	one	446 ± 1	18767.28	9970
N414L/Q513L*	slow	6.1	11.5	one	4507 ± 168	18776.29	9970

Table S8: Experimental validation of all third-round ML predictions. Shown are the prediction, flavin loading (%), recovery kinetics ($\tau_{FMN}(sec)$) and if the experimental result matches its prediction (color coded in column τ_{FMN}: green: matching experimental outcome and prediction, red: experiment and prediction did not match). Adduct state lifetimes τ_{FMN} are reported as means of three technical replicates with the error representing the standard deviation of the mean. Faster and slower variants were selected based on a benchmarking dataset for three independently prepared and measured AsLOV2 wild type samples. A predicted variant was considered faster or slower than AsLOV2 wildtype, if its adduct state lifetime is at least two standard deviations faster (<34 sec) or slower (>52 sec) than the wildtype (WT). Like WT: Predicted variant is like WT (34-52 sec). No: Selection/ prediction is wrong. *: Tends to aggregate. n.a. Not applicable. $^{\%}$: in case a two phase exponential appeared better suited to fit the data (judged from residuals; see Materials and Methods section) τ_{FMN} is preseted as average value according to $\tau_{FMN} = \sum(A_i\tau_i) / 100$.

**Fast**							
**AsLOV2 variant**	**Recovery prediction**	**Concentration [mg/ml]**	**FMN loading [%]**	**One phase or two phase exponential fit**	**τ_{FMN} [sec]**	**Molecular weight [Da]**	**Extinction coefficient [1/M*cm]**
WT	n.a.	3.00	39.6	one	43 ± 4.5	18792.20	9970
T438V/E475T	fast	3.27	40.5	one	17.7 ± 0.7	16598.90	9970
E475T/L480A	fast	n.a	n.a.	n.a	could not be generated	16558.79	9970
L446F/E475T	fast	-8909.68	—	n.a	—	16634.89	9970
V463W/E475T	fast	2.02	50.7	one	46.3 ± 1.6	16687.95	15470
I427T/L446M/E475T	fast	1.36	12.0	one	0.40 ± 0.01	16606.85	9970
**Slow**							
**AsLOV2 variant**	**Recovery prediction**	**Concentration [mg/ml]**	**FMN loading [%]**	**One phase or two phase exponential fit**	**τ_{FMN} [sec]**	**Molecular weight [Da]**	**Extinction coefficient [1/M*cm]**
WT	n.a.	3.00	39.6	one	43 ± 4.5	18792.20	9970
N414D/V416A	slow	2.06	—	n.a.	—	16601.81	9970
N414D/V416T	slow	2.10	1.4	**two**	9.6 ± 1.9	16631.84	9970
N414M/F415E/V416A	slow	1.09	—	n.a.	—	16599.85	9970
N414D/F415E/V416T	slow	2.03	0.4	one	17.2 ± 0.1	16613.78	9970

Table S9: Bacterial strains and plasmids used in this study.

**Bacterial strain**	**Relevant genotype**	**Reference**
Escherichia coli DH5α	F– Φ80*lac*ZΔM15 Δ(*lac*ZYA-*arg*F) U169 *rec*A1 *end*A1 *hsd*R17 (r_K^-, m_K^+) *gal*⁻ *pho*A *sup*E44 λ⁻ *thi*⁻1 *gyr*A96 *rel*A1	Woodcock *et al.*, 1989[18]
Escherichia coli BL21(DE3)	F– *ompT gal dcm lon hsd*S_B(r_B^-, m_B^-) λ(DE3[*lac*I *lac*UV5-T7 gene 1 *ind*1 *sam*7 *nin*5])	Studier & Moffat 1986[19]
Escherichia coli CMPX 131	Derivate of CMPX13, (ribCΔ and ribM)	Mathes *et al.*, 2009[20]
**Plasmid name**	**Relevant features**[§]	**Reference**
pET28a	*ColE1 lacI,* Kan^R P_{T7} P_{lac} N-terminal His-tag	Novagen, Merck, Darmstadt, Germany
pET28a-AsLOV2	pET28a derivative, P_{T7}>AsLOV2 (wildtype) subloned from pMA-RQ-AsLOV2	Hemmer[21]
pET28a-AsLOV2-T406C	pET28a derivative, P_{T7}>AsLOV2-T406C (T406C: ACT>TGC)	This work
pET28a-AsLOV2-T406V	pET28a derivative, P_{T7}>AsLOV2-T406V (T406V: ACT>GTG)	This work
pET28a-AsLOV2-R410P	pET28a derivative, P_{T7}>AsLOV2-R410P (R410P: CGT>CCG)	This work
pET28a-AsLOV2-K413A	pET28a derivative, P_{T7}>AsLOV2-K413A (K413A: AAG>GCG)	This work
pET28a-AsLOV2-K413C	pET28a derivative, P_{T7}>AsLOV2-K413C (K413C: TGC>GCG)	This work
pET28a-AsLOV2-N414C	pET28a derivative, P_{T7}>AsLOV2-N414C (N414C: AAC>TGC)	This work
pET28a-AsLOV2-N414E	pET28a derivative, P_{T7}>AsLOV2-N414E (N414E: AAC>GAA)	This work
pET28a-AsLOV2-N414G	pET28a derivative, P_{T7}>AsLOV2-N414G (N414G: AAC>GGC)	This work
pET28a-AsLOV2-N414L	pET28a derivative, P_{T7}>AsLOV2-N414L (N414L: AAC>CTG)	This work
pET28a-AsLOV2-N414M	pET28a derivative, P_{T7}>AsLOV2-N414M (N414M: AAC>ATG)	This work
pET28a-AsLOV2-V416E	pET28a derivative, P_{T7}>AsLOV2-V416E (V416E: GTC>GAA)	This work
pET28a-AsLOV2-V416M	pET28a derivative, P_{T7}>AsLOV2-V416M (V416M: GTC>ATG)	This work
pET28a-AsLOV2-V416T	pET28a derivative, P_{T7}>AsLOV2-V416T (V416T: GTC>ACC)	This work
pET28a-AsLOV2-I427N	pET28a derivative, P_{T7}>AsLOV2-I427N (I427N: ATT>AAC)	This work
pET28a-AsLOV2-I427T	pET28a derivative, P_{T7}>AsLOV2-I427T (I427T: ATT>ACC)	This work
pET28a-AsLOV2-L446M	pET28a derivative, P_{T7}>AsLOV2-L446M (L446M: TTG>ATG)	This work
pET28a-AsLOV2-L446Q	pET28a derivative, P_{T7}>AsLOV2-L446Q (L446Q: TTG>CAG)	This work
pET28a-AsLOV2-L446S	pET28a derivative, P_{T7}>AsLOV2-L446S (L446S: TTG>AGC)	This work
pET28a-AsLOV2-N449A	pET28a derivative, P_{T7}>AsLOV2-N449A (N449A: AAC>GCG)	This work

pET28a-AsLOV2-N449E	pET28a derivative, P_{T7}>AsLOV2-N449E (N449E: AAC>GAA)	This work
pET28a-AsLOV2-N449I	pET28a derivative, P_{T7}>AsLOV2-N449I (N449I: AAC>ATT)	This work
pET28a-AsLOV2-N449K	pET28a derivative, P_{T7}>AsLOV2-N449K (N449K: AAC>AAA)	This work
pET28a-AsLOV2-N449T	pET28a derivative, P_{T7}>AsLOV2-N449T (N449T: AAC>ACC)	This work
pET28a-AsLOV2-E475T	pET28a derivative, P_{T7}>AsLOV2-E475T (E475T: GAG>ACC)	This work
pET28a-AsLOV2-L496K	pET28a derivative, P_{T7}>AsLOV2-L496K (L496K: TTG>AAA)	This work
pET28a-AsLOV2-D501G	pET28a derivative, P_{T7}>AsLOV2-D501G (D501G: GAT>GGC)	This work
pET28a-AsLOV2-D501W	pET28a derivative, P_{T7}>AsLOV2-D501W (D501W: GAT>TGG)	This work
pET28a-AsLOV2-D501Y	pET28a derivative, P_{T7}>AsLOV2-D501Y (D501Y: GAT>TAT)	This work
pET28a-AsLOV2-D515L	pET28a derivative, P_{T7}>AsLOV2-D515L (D515L: GAT>CTG)	This work
pET28a-AsLOV2-Q513H	pET28a derivative, P_{T7}>AsLOV2-Q513H (Q513H: CAG>CAT)	This work
pET28a-AsLOV2-Q513P	pET28a derivative, P_{T7}>AsLOV2-Q513P (Q513P: CAG>CCG)	This work
pET28a-AsLOV2-Q513R	pET28a derivative, P_{T7}>AsLOV2-Q513R (Q513R: CAG>CGC)	This work
pET28a-AsLOV2-G528E	pET28a derivative, P_{T7}>AsLOV2-G528E (G528E: GGA>GAA)	This work
pET28a-AsLOV2-G528K	pET28a derivative, P_{T7}>AsLOV2-G528K (G528K: GGA>AAA)	This work
pET28a-AsLOV2-G528R	pET28a derivative, P_{T7}>AsLOV2-G528R (G528R: GGA>CGC)	This work
pET28a-AsLOV2-R410P/G528A	pET28a derivative, P_{T7}>AsLOV2-R410P/G528A (R410P: CGT>CCG; G528A: GAA>GCG)	This work
pET28a-AsLOV2-R410P/G528E	pET28a derivative, P_{T7}>AsLOV2-R410P/G528E (R410P: CGT>CCG; G528E: GGA>GAA)	This work
pET28a-AsLOV2-R410P/G528R	pET28a derivative, P_{T7}>AsLOV2-R410P/G528R (R410P: CGT>CCG; G528R: GGA>CGC)	This work
pET28a-AsLOV2-K413A/N414G	pET28a derivative, P_{T7}>AsLOV2-K413A/N414G (K413A: AAG>GCG; N414G: AAC>GGC)	This work
pET28a-AsLOV2-K413A/D501G	pET28a derivative, P_{T7}>AsLOV2-K413A/D501G (K413A: AAG>GCG; D501G: GAT>GGC)	This work
pET28a-AsLOV2-K413C/N414C	pET28a derivative, P_{T7}>AsLOV2-K413C/N414C (K413C: TGC>GCG; N414C: AAC>TGC)	This work
pET28a-AsLOV2-N414A/V416A	pET28a derivative, P_{T7}>AsLOV2-N414A/V416A (N414A: AAC>GCG; V416A: GTC>GCG)	This work
pET28a-AsLOV2-N414A/V416L	pET28a derivative, P_{T7}>AsLOV2-N414A/V416L (N414A: AAC>GCG; V416L: GTC>CTG)	This work
pET28a-AsLOV2-N414C/L496K	pET28a derivative, P_{T7}>AsLOV2-N414C/L496K (N414C: AAC>TGC; L496K: TTG>AAA)	This work
pET28a-AsLOV2-N414D/V416A	pET28a derivative, P_{T7}>AsLOV2-N414D/V416A (N414D: AAC>GAT; V416A: GTC>GCG)	This work

pET28a-AsLOV2-N414D/V416T	pET28a derivative, P_{T7}> AsLOV2-N414D/V416T (N414D: AAC>GAT; V416T: GTC>ACC)	This work
pET28a-AsLOV2-N414E/V416E	pET28a derivative, P_{T7}>AsLOV2-N414E/V416E (N414E: AAC>GAA; V416E: GTC>GAA)	This work
pET28a-AsLOV2-N414E/V416M	pET28a derivative, P_{T7}>AsLOV2-N414E/V416M (N414E: AAC>GAA; V416M: GTC>ATG)	This work
pET28a-AsLOV2-N414G/L446E	pET28a derivative, P_{T7}>AsLOV2-N414G/L446E (N414G: AAC>GGC; L446E: TTG>GAG)	This work
pET28a-AsLOV2-N414G/L446S	pET28a derivative, P_{T7}>AsLOV2-N414G/L446S (N414G: AAC>GGC; L446S: TTG>AGC)	This work
pET28a-AsLOV2-N414G/G528E	pET28a derivative, P_{T7}>AsLOV2-N414G/G528E (N414G: AAC>GGC; G528E: GGA>GAA)	This work
pET28a-AsLOV2-N414L/V416L	pET28a derivative, P_{T7}>AsLOV2-N414L/V416L (N414L: AAC>CTG; V416L: GTC>CTG)	This work
pET28a-AsLOV2-N414L/H495L	pET28a derivative, P_{T7}>AsLOV2-N414L/H495L (N414L: AAC>CTG; H495L: CAC>CTG)	This work
pET28a-AsLOV2-N414L/Q513L	pET28a derivative, P_{T7}>AsLOV2-N414L/Q513L (N414L: AAC>CTG; Q513L: CAG>CTG)	This work
pET28a-AsLOV2-N414L/L514A	pET28a derivative, P_{T7}>AsLOV2-N414L/L514A (N414L: AAC>CTG; L514A: TTG>GCG)	This work
pET28a-AsLOV2-N414M/V416E	pET28a derivative, P_{T7}>AsLOV2-N414M/V416E (N414M: AAC>ATG; V416E: GTC>GAA)	This work
pET28a-AsLOV2-N414M/V416M	pET28a derivative, P_{T7}>AsLOV2-N414M/V416M (N414M: AAC>ATG; V416M: GTC>ATG)	This work
pET28a-AsLOV2-V416T/N449T	pET28a derivative, P_{T7}>AsLOV2-V416T/N449T (V416T: GTC>ACC; N449T: AAC>ACC)	This work
pET28a-AsLOV2-I427N/N449A	pET28a derivative, P_{T7}>AsLOV2-I427N/N449A (I427N: ATT>AAC; N449A: AAC>GCG)	This work
pET28a-AsLOV2-I427N/N449E	pET28a derivative, P_{T7}>AsLOV2-I427N/N449E (I427N: ATT>AAC; N449E: AAC>GAA)	This work
pET28a-AsLOV2-I427N/N449I	pET28a derivative, P_{T7}>AsLOV2-I427N/N449I (I427N: ATT>AAC; N449I: AAC>ATT)	This work
pET28a-AsLOV2-I427N/N449K	pET28a derivative, P_{T7}>AsLOV2-I427N/N449K (I427N: ATT>AAC; N449K: AAC>AAA)	This work
pET28a-AsLOV2-I427T/N449T	pET28a derivative, P_{T7}>AsLOV2-I427T/N449T (I427T: ATT>ACC; N449T: AAC>ACC)	This work
pET28a-AsLOV2-I427T/E475T	pET28a derivative, P_{T7}>AsLOV2-I427T/E475T (I427T: ATT>ACC; E475T: GAG>ACC)	This work
pET28a-AsLOV2-T438V/E475T	pET28a derivative, P_{T7}>AsLOV2-T438V/E475T (T438V: ACA>GTG; E475T: GAG>ACC)	This work
pET28a-AsLOV2-R442L/D515L	pET28a derivative, P_{T7}>AsLOV2-R442L/D515L (R442L: CGT>CTG; D515L: GAT>CTG)	This work
pET28a-AsLOV2-L446F/E475T	pET28a derivative, P_{T7}>AsLOV2-L446F/E475T (L446F: TTG>TTT; E475T: GAG>ACC)	This work
pET28a-AsLOV2-L446M/D501W	pET28a derivative, P_{T7}>AsLOV2-L446M/D501W (L446M: TTG>ATG; D501W: GAT>TGG)	This work
pET28a-AsLOV2-L446Q/D501Y	pET28a derivative, P_{T7}>AsLOV2-L446Q/D501Y (L446Q: TTG>CAG; D501Y: GAT>TAT)	This work
pET28a-AsLOV2-L446S/D501Y	pET28a derivative, P_{T7}>AsLOV2-L446S/D501Y (L446S: TTG>AGC; D501Y: GAT>TAT)	This work

pET28a-AsLOV2-N449T/Q513P	pET28a derivative, P_{T7}>AsLOV2-N449T/Q513P (N449T: AAC>ACC; Q513P: CAG>CCG)	This work
pET28a-AsLOV2-V463W/E475T	pET28a derivative, P_{T7}>V463W/E475T (V463W: GTG>TGG; E475T: GAG>ACC)	This work
pET28a-AsLOV2-E475T/G528E	pET28a derivative, P_{T7}>AsLOV2-E475T/G528E (E475T: GAG>ACC; G528E: GGA>GAA)	This work
pET28a-AsLOV2-D501G/G528E	pET28a derivative, P_{T7}>AsLOV2-D501G/G528E (D501G: GAT>GGC; G528E: GGA>GAA)	This work
pET28a-AsLOV2-Q513R/G528R	pET28a derivative, P_{T7}>AsLOV2-Q513R/G528R (Q513R: CAG>CGC; G528R: GGA>CGC)	This work
pET28a-AsLOV2-Q513R/G528K	pET28a derivative, P_{T7}>AsLOV2-Q513R/G528R (Q513R: CAG>CGC; G528K: GGA>AAA)	This work
pET28a-AsLOV2-N414D/F415E/V416T	pET28a derivative, P_{T7}>AsLOV2-N414D/F415E/V416T (N414D: AAC>GAT; F415E: TTT>GAA; V416T: GTC>ACC)	This work
pET28a-AsLOV2-N414M/F415E/V416A	pET28a derivative, P_{T7}>AsLOV2-N414M/F415E/V416A (N414M: AAC>ATG; F415E: TTT>GAA; V416A: GTC>GCG)	This work
pET28a-AsLOV2-I427T/L446M/E475T	pET28a derivative, P_{T7}>AsLOV2-I427T/L446M/E475T (I427T: ATT>ACC; L446M: TTG>ATG; E475T: GAG>ACC)	This work

Table S10: Oligonucleotide primers used in this study. The exchanged codon is shown in bold red.

**Oligo name**	**Sequence (5’→3’)**
AsLOV2-T406C_*fw*	TTG GCT **TGC** ACA CTT GAA CGT ATT GAG AAG AAC TT
AsLOV2-T406C_*rev*	TTC TTC TCA ATA CGT TCA AGT GT**G CA**A GCC AAC AT
AsLOV2-T406V_*fw*	TTG GCT **GTG** ACA CTT GAA CGT ATT GAG AAG AAC TT
AsLOV2-T406V_*rev*	TTC TTC TCA ATA CGT TCA AGT GT**C AC**A GCC AAC AT
AsLOV2-R410P_*fw*	TTG AA**C CG**A TTG AGA AGA ACT TTG TCA T
AsLOV2-R410P_*rev*	CAA T**CG G**TT CAA GTG TAG TAG CCA ACA T
AsLOV2-K413A_*fw*	TTG AG**G CG**A ACT TTG TCA TTA CTG ACC
AsLOV2-K413A_*rev*	AAG TT**C GC**C TCA ATA CGT TCA AGT GTA G
AsLOV2-K413C_*fw*	ATT GAG **TGC** AAC TTT GTC ATT ACT GAC CCA AGG TT
AsLOV2-K413C_*rv*	AAA GTT **GCA** CTC AAT ACG TTC AAG TGT AGT AGC CA
AsLOV2-N414C_*fw*	GAG AAG **TGC** TTT GTC ATT ACT GAC CCA AGG TTG CC
AsLOV2-N414C_*rev*	GAC AAA **GCA** CTT CTC AAT ACG TTC AAG TGT AGT AG
AsLOV2-N414E_*fw*	GAG AAG **GAA** TTT GTC ATT ACT GAC CCA AGG TTG CC
AsLOV2-N414E_*rev*	GAC AAA **TTC** CTT CTC AAT ACG TTC AAG TGT AGT AG
AsLOV2-N414G_*fw*	AGA AG**G GC**T TTG TCA TTA CTG ACC CAA G
AsLOV2-N414G_*rev*	ACA AA**G CC**C TTC TCA ATA CGT TCA AGT G
AsLOV2-N414L_*fw*	AGA AG**C TG**T TTG TCA TTA CTG ACC CAA G
AsLOV2-N414L_*rev*	ACA AA**C AG**C TTC TCA ATA CGT TCA AGT G
AsLOV2-N414M_*fw*	GAG AAG **ATG** TTT GTC ATT ACT GAC CCA AGG TTG CC
AsLOV2-N414M_*rev*	GAC AAA **CAT** CTT CTC AAT ACG TTC AAG TGT AGT AG
AsLOV2-V416E_*fw*	AAC TTT **GAA** ATT ACT GAC CCA AGG TTG CCA GAT AA
AsLOV2-V416E_*rev*	AGT AAT **TTC** AAA GTT CTT CTC AAT ACG TTC AAG TG
AsLOV2-V416M_*fw*	AAC TTT **ATG** ATT ACT GAC CCA AGG TTG CCA GAT AA
AsLOV2-V416M_*rev*	AGT AAT **CAT** AAA GTT CTT CTC AAT ACG TTC AAG TG
AsLOV2-V416T_*fw*	AAC TTT **ACC** ATT ACT GAC CCA AGG TTG CCA GAT AA
AsLOV2-V416T_*rev*	AGT AAT **GGT** AAA GTT CTT CTC AAT ACG TTC AAG TG
AsLOV2-I427N_*fw*	AAT CCC **AAC** ATA TTC GCG TCC GAT AGT TTC TTG CA
AsLOV2-I427N_*rev*	GAA TAT **GTT** GGG ATT ATC TGG CAA CCT TGG GTC AG
AsLOV2-I427T_*fw*	AAT CCC **ACC** ATA TTC GCG TCC GAT AGT TTC TTG CA
AsLOV2-I427T_*rev*	GAA TAT **GGT** GGG ATT ATC TGG CAA CCT TGG GTC AG
AsLOV2-T438V_*fw*	AGT TG**G TG**G AAT ATA GCC GTG AAG AAA TTT TGG

AsLOV2-T438V_*rv*	TAT TC**C AC**C AAC TGC AAG AAA CTA TCG GAC
AsLOV2-R442L_*fw*	ATA GC**C TG**G AAG AAA TTT TGG GAA GAA CT
AsLOV2-R442L_*rev*	TCT TC**C AG**G CTA TAT TCT GTC AAC TGC A
AsLOV2-L446C_*fw*	GAA ATT **TGC** GGA AGA AAC TGC AGG TTT CTA CAA GG
AsLOV2-L446C_*rev*	TCT TCC **CCA** AAT TTC TTC ACG GCT ATA TTC TGT CA
AsLOV2-L446E_*fw*	AAA TT**G AG**G GAA GAA ACT GCA GGT TTC T
AsLOV2-L446E_*rev*	CTT CC**C TC**A ATT TCT TCA CGG CTA TAT T
AsLOV2-L446F_*fw*	AAA TT**T TT**G GAA GAA ACT GCA GGT TTC TAC AAG
AsLOV2-L446F_*rev*	CTT CC**A AA**A ATT TCT TCA CGG CTA TAT TCT GTC
AsLOV2-L446M_*fw*	GAA ATT **ATG** GGA AGA AAC TGC AGG TTT CTA CAA GG
AsLOV2-L446M_*rev*	TCT TCC **CAT** AAT TTC TTC ACG GCT ATA TTC TGT CA
AsLOV2-L446Q_*fw*	GAA ATT **CAG** GGA AGA AAC TGC AGG TTT CTA CAA GG
AsLOV2-L446Q_*rev*	TCT TCC **CTG** AAT TTC TTC ACG GCT ATA TTC TGT CA
AsLOV2-L446S_*fw*	GAA ATT **AGC** GGA AGA AAC TGC AGG TTT CTA CAA GG
AsLOV2-L446S_*rev*	TCT TCC **GCT** AAT TTC TTC ACG GCT ATA TTC TGT CA
AsLOV2-N449A_*fw*	GGA AGA **GCG** TGC AGG TTT CTA CAA GGT CC
AsLOV2-N449A_*rev*	CCT GCA **CGC** TCT TCC CAA AAT TTC TTC ACG G
AsLOV2-N449E_*fw*	GGA AGA **GAA** TGC AGG TTT CTA CAA GGT CCT GAA A
AsLOV2-N449E_*rev*	CCT GCA **TTC** TCT TCC CAA AAT TTC TTC ACG GCT AT
AsLOV2-N449I_*fw*	GGA AGA **ATT** TGC AGG TTT CTA CAA GGT CCT GAA A
AsLOV2-N449I_*rev*	CCT GCA **AAT** TCT TCC CAA AAT TTC TTC ACG GCT AT
AsLOV2-N449K_*fw*	GGA AGA **AAA** TGC AGG TTT CTA CAA GGT CCT GAA A
AsLOV2-N449K_*rev*	CCT GCA **TTT** TCT TCC CAA AAT TTC TTC ACG GCT AT
AsLOV2-N449T_*fw*	GGA AGA **ACC** TGC AGG TTT CTA CAA GGT CCT G
AsLOV2-N449T_*rev*	CCT GCA **GGT** TCT TCC CAA AAT TTC TTC ACG
AsLOV2-V463W_*fw*	CGA CA**T GG**A GAA AAA TTA GAG ATG CCA TAG ATA
AsLOV2-V463W_*rev*	TTT CT**C CA**T GTC GCG CGA TCA GTT TCA
AsLOV2-E475T_*fw*	CAA ACA **ACC** GTC ACT GTT CAG CTG ATT AAT TAT AC
AsLOV2-E475T_*rev*	AGT GAC **GGT** TGT TTG GTT ATC TAT GGC ATC TCT AA
AsLOV2-L480A_*fw*	TTC AG**G CG**A TTA ATT ATA CAA AGA GTG GTA AAA A
AsLOV2-L480A_*rev*	TTA AT**C GC**C TGA ACA GTG ACG GTT GT
AsLOV2-H495L_*fw*	TCT TT**C TG**T TGC AGC CTA TGC GAG ATC A
AsLOV2-H495L_*rev*	TGC AA**C AG**A AAG AGG TTC CAG AAC TTT T

AsLOV2-L496K_*fw*	TTT CAC **AAA** CAG CCT ATG CGA GAT CAG AAG
AsLOV2-L496K_*rev*	AGG CTG **TTT** GTG AAA GAG GTT CCA GAA CTT TTT AC
AsLOV2-D501G_*fw*	TGC GA**G GC**C AGA AGG GAG ATG TCC AGT A
AsLOV2-D501G_*rev*	TTC TG**G CC**T CGC ATA GGC TGC AAG TGA A
AsLOV2-D501W_*fw*	ATG CGA **TGG** CAG AAG GGA GAT GTC CAG TA
AsLOV2-D501W_*rev*	CTT CTG **CCA** TCG CAT AGG CTG CAA GT
AsLOV2-D501Y_*fw*	ATG CGA **TAT** CAG AAG GGA GAT GTC CAG TAC TTT AT
AsLOV2-D501Y_*rev*	CTT CTG **ATA** TCG CAT AGG CTG CAA GTG AAA GAG GT
AsLOV2-D515L_*fw*	AGT TG**C TG**G GAA CTG AGC ATG TCC GA
AsLOV2-D515L_*rev*	GTT CC**C AG**C AAC TGA ACC CCA ATA AAG T
AsLOV2-Q513H_*fw*	GGG GTT **CAT** TTG GAT GGA ACT GAG CAT GTC C
AsLOV2-Q513H_*rev*	ATC CAA **ATG** AAC CCC AAT AAA GTA CTG GAC ATC TC
AsLOV2-Q513L_*fw*	GGG TT**C TG**T TGG ATG GAA CTG AGC ATG T
AsLOV2-Q513L_*rev*	TCC AA**C AG**A ACC CCA ATA AAG TAC TGG A
AsLOV2-Q513P_*fw*	GGG GTT **CCG** TTG GAT GGA ACT GAG CAT GTC
AsLOV2-Q513P_*rev*	ATC CAA **CGG** AAC CCC AAT AAA GTA CTG GAC ATC
AsLOV2-Q513R_*fw*	GGG GTT **CGC** TTG GAT GGA ACT GAG CAT GTC
AsLOV2-Q513R_*rev*	ATC CAA **GCG** AAC CCC AAT AAA GTA CTG GAC ATC
AsLOV2-L514A_*fw*	TTC AG**G CG**G ATG GAA CTG AGC ATG T
AsLOV2-L514A_*rev*	CCA TC**C GC**C TGA ACC CCA ATA AAG TAC T
AsLOV2- G528A_*fw*	GAG AG**G CG**G TCA TGC TGA TTA AGA AAA C
AsLOV2-G528A_*rev*	ATG AC**C GC**C TCT CTC TCG GC
AsLOV2-G528E_*fw*	AGA GAG **GAA** GTC ATG CTG ATT AAG AAA ACT GCA GA
AsLOV2-G528E_*rev*	CAT GAC **TTC** CTC TCT CTC GGC AGC ATC TCG GAC
AsLOV2-G528H_*fw*	AGA GAG **CAG** GTC ATG CTG ATT AAG AAA ACT GC
AsLOV2-G528H_*rev*	CAT GAC **CTG** CTC TCT CTC GGC AGC ATC TC
AsLOV2-G528K_*fw*	AGA GAG **AAA** GTC ATG CTG ATT AAG AAA ACT GCA GA
AsLOV2-G528K_*rev*	CAT GAC **TTT** CTC TCT CTC GGC AGC ATC TCG GAC
AsLOV2-G528R_*fw*	AGA GAG **CGC** GTC ATG CTG ATT AAG AAA ACT GCA
AsLOV2-G528R_*rev*	CAT GAC **GCG** CTC TCT CTC GGC AGC ATC TC
AsLOV2-K413A/N414G_*fw*	TTG AG**G CGG GC**T TTG TCA TTA CTG ACC
AsLOV2-K413A/N414G_*rev*	ACA AA**G CCC GC**C TCA ATA CGT TCA AGT GTA G
AsLOV2-K413C/N414C_*fw*	ATT GAG **TGC TGC** TTT GTC ATT ACT GAC CCA AGG TT

AsLOV2-K413C/N414C_*rev*	AAA **GCA GCA** CTC AAT ACG TTC AAG TGT AGT AGC CA
AsLOV2-N414A/V416A_*fw*	AGA AG**G CG**T TT**G CG**A TTA CTG ACC CAA GGT T
AsLOV2-N414A/V416A_*rev*	GTA AT**C GC**A AA**C GC**C TTC TCA ATA CGT TCA AGT G
AsLOV2-N414A/V416L_*fw*	AGA AG**G CG**T TT**C TG**A TTA CTG ACC CAA GGT T
AsLOV2-N414A/V416L_*rev*	GTA AT**C AG**A AA**C GC**C TTC TCA ATA CGT TCA AGT G
AsLOV2-N414D/V416A_*fw*	AGA AG**G AT**T TT**G CG**A TTA CTG ACC CAA GGT TG
AsLOV2-N414D/V416A_*rev*	GTA AT**C GC**A AA**A TC**C TTC TCA ATA CGT TCA AGT GTA GTA
AsLOV2-N414D/V416T_*fw*	AGA AG**G AT**T TT**A CC**A TTA CTG ACC CAA GGT TGC CAG ATA
AsLOV2-N414D/V416T_*rev*	GTA AT**G GT**A AA**A TC**C TTC TCA ATA CGT TCA AGT GTA GTA
AsLOV2-N414E/V416E_*fw*	GAG AAG **GAA** TTT **GAA** ATT ACT GAC CCA AGG TTG CC
AsLOV2-N414E/V416E_*rev*	**TTC** AAA **TTC** CTT CTC AAT ACG TTC AAG TGT AGT AG
AsLOV2-N414E/V416M_*fw*	GAG AAG **GAA** TTT **ATG** ATT ACT GAC CCA AGG TTG CC
AsLOV2-N414E/V416M_*rev*	**CAT** AAA **TTC** CTT CTC AAT ACG TTC AAG TGT AGT AG
AsLOV2-N414L/V416L_*fw*	AGA AG**C TG**T TT**C TG**A TTA CTG ACC CAA GGT T
AsLOV2-N414L/V416L_*rev*	GTA AT**C AG**A AA**C AG**C TTC TCA ATA CGT TCA AGT G
AsLOV2-N414M/V416E_*fw*	GAG AAG **ATG** TTT **GAA** ATT ACT GAC CCA AGG TTG CC
AsLOV2-N414M/V416E_*rev*	**TTC** AAA **CAT** CTT CTC AAT ACG TTC AAG TGT AGT AG
AsLOV2-N414M/V416M_*fw*	GAG AAG **ATG** TTT **ATG** ATT ACT GAC CCA AGG TTG CC
AsLOV2-N414M/V416M_*rev*	**CAT** AAA **CAT** CTT CTC AAT ACG TTC AAG TGT AGT AG
AsLOV2-N414D/F415E/V416T_*fw*	AGA AG**G ATG AAA CC**A TTA CTG ACC CAA GGT TG
AsLOV2-N414D/F415E/V416T_*rev*	AT**G GTT TCA TC**C TTC TCA ATA CGT TCA AGT GTA GTA
AsLOV2-N414M/F415E/V416A_*fw*	AG**A TGG AAG CG**A TTA CTG ACC CAA GGT TG
AsLOV2-N414M/F415E/V416A_*rev*	GT**A ATC GCT TC**C ATC TTC TCA ATA CGT TCA AGT

Table S11: Summary of measurement duration (min), averaging time (sec) and cycle time (min) of dark recovery measurements of all measurable AsLOV2 variants.

**Protein**	**Measurement duration (min)**	**Ave Time (sec)**	**Cycle Time (min)**
AsLOV2-T406C	5	0.1	0.03
AsLOV2-T406V	5	0.1	0.03
AsLOV2-R410P	10	0.1	0.02
AsLOV2-K413A	5	0.1	0.01
AsLOV2-K413C	10	0.05	0.02
AsLOV2-N414C	90	0.1	0.5
AsLOV2-N414E	30	0.1	0.03
AsLOV2-N414G	20	0.1	0.02
AsLOV2-N414L	120	0.1	0.02
AsLOV2-N414M	90	0.1	0.5
AsLOV2-V416T	3	0.05	0.01
AsLOV2-I427N	5	0.05	0.02
AsLOV2-I427T	3	0.025	0.01
AsLOV2-L446M	5	0.1	0.02
AsLOV2-L446Q	5	0.1	0.02
AsLOV2-L446S	5	0.1	0.02
AsLOV2-N449E	5	0.1	0.02

AsLOV2-E475T	4	0.1	0.05
AsLOV2-D501G	5	0.1	0.01
AsLOV2-D501W	5	0.1	0.02
AsLOV2-D501Y	10	0.1	0.02
AsLOV2-D515L	8	0.1	0.01
AsLOV2-Q513H	3	0.025	0.01
AsLOV2-Q513P	60	0.1	0.5
AsLOV2-Q513R	25	0.1	0.05
AsLOV2-G528E	5	0.1	0.02
AsLOV2-G528K	3	0.1	0.03
AsLOV2-G528R	3	0.05	0.02
AsLOV2-R410P/G528A	10	0.1	0.02
AsLOV2-R410P/G528E	10	0.1	0.02
AsLOV2-R410P/G528R	10	0.1	0.02
AsLOV2-K413A/N414G	90	0.1	0.03
AsLOV2-K413A/D501G	5	0.1	0.01
AsLOV2-K413C/N414C	30	0.1	0.02
AsLOV2-N414D/V416T	3	0.1	0.01
AsLOV2-N414G/L446E	10	0.1	0.02
AsLOV2-N414G/L446S	40	0.1	0.05

AsLOV2-N414G/G528E	40	0.1	0.03
AsLOV2-N414L/L514A	120	0.1	0.05
AsLOV2- N414L/H495L	45	0.1	0.05
AsLOV2-I427T/E475T	3	0.025	0.01
AsLOV2-T438V/E475T	6	0.1	0.02
AsLOV2-R442L/D515L	10	0.1	0.02
AsLOV2-L446M/D501W	5	0.1	0.02
AsLOV2-L446Q/D501Y	3	0.1	0.01
AsLOV2-L446S/D501Y	5	0.1	0.01
AsLOV2-V463W/E475T	6	0.1	0.02
AsLOV2-E475T/G528E	5	0.1	0.01
AsLOV2-D501G/G528E	5	0.1	0.01
AsLOV2-Q513R/G528K	10	0.1	0.02
AsLOV2-Q513R/G528R	10	0.1	0.02
AsLOV2-N414D/F415E/V416T	3	0.1	0.01
AsLOV2-I427T/L446M/E475T	2	0.1	0.01

Table S12: Summary of measurement duration (min), time intervals (min) and number of measured dark recovery spectra of AsLOV2-N414A/V416A, -N414A/V416L, -N414L/V416L and -N414L/Q513A.

Protein sample	Duration of measurement (min)	Time interval (min)	Number of spectra
AsLOV2-N414A/V416A	20	0.2	100
AsLOV2-N414A/V416L	7200	30	240
AsLOV2-N414L/V416L	7999	30	266.6
AsLOV2-N414L/Q513L	300	0.5	600

Table S13: Recovery kinetic time constants for variants showing a two-phase decay. Average τ_{FMN} values used for ML-predictions, mapping and plotting purposes are shown in Table S4-S8.

AsLOV2 variant	$\tau_{FMN,slow}$ [sec]	$A_{FMN,slow}$	$\tau_{FMN,fast}$	$A_{FMN,fast}$ [sec]
N414E	217.7 ± 2.1	86.8 ± 4.2	57.7 ± 0.5	13.2 ± 4.2
N414M	757.6 ± 19.3	83.9 ± 0.5	83.9 ± 32.0	16.1 ± 0.5
K413C/N414C	193.8 ± 5.8	79.3 ± 1.5	28.0 ± 12.2	20.7 ±1.5
N414G	262.0 ± 26.6	78.2 ± 13.4	42.9 ± 35.3	21.8 ± 13.4
N414G/G528E	256.8 ± 14.3	92.5 ± 5.4	70.8 ± 36.8	7.5 ± 5.4
R410P/G528A	62.1 ± 1.6	94.0 ± 0.7	14.8 ± 2.8	6.0 ± 0.7
R410P/G528E	68.4 ± 11.1	64.3 ± 26.4	42.1 ± 3.7	36.7 ± 26.4
K413A/N414G	1302.3 ± 377.2	27.5 ± 0.8	206.8 ± 4.5	72.5 ± 0.8
N414G/L446S	631.2 ± 11.0	81.1 ± 0.1	83.2 ± 7.0	18.9 ± 0.1
N414D/V416T	33.4 ± 12.9	24.9 ± 5.1	2.1 ± 0.4	75.1 ± 5.1

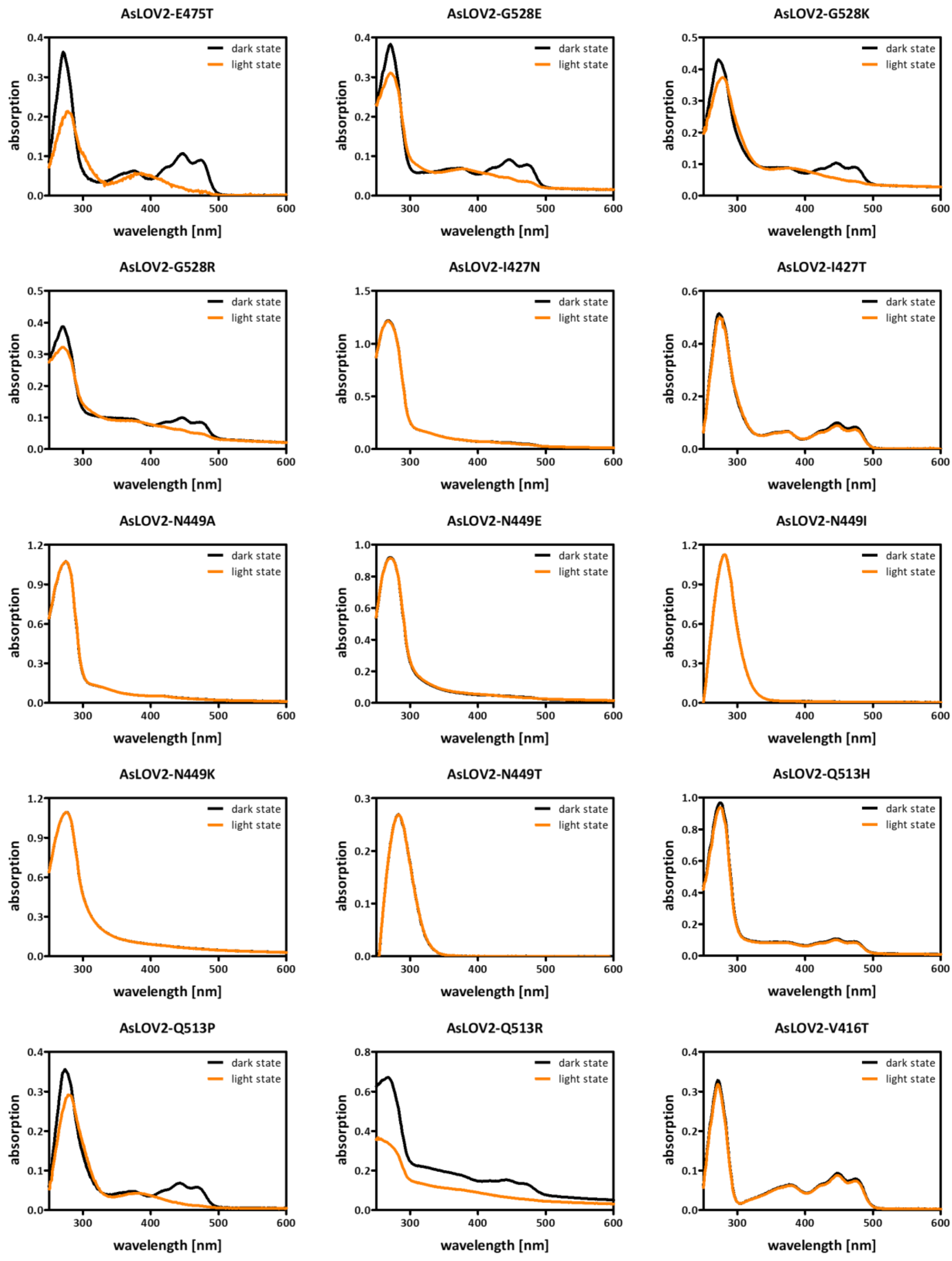

Figure S1: Absorption spectra of the dark and light states of first round selected fast single AsLOV2 variants. All samples were diluted with storage and dark recovery buffer (50 mM tris, 1 mM EDTA, 5 mM DTT; pH-value 8.0) to an OD_{450nm} of approximately 0.1 and the dark state spectrum was recorded (black line). Subsequently, the samples were illuminated for 1 min with a blue light LED (λ=440 nm, 2.6 mW/cm^2[22], Luxeon Lumileds, Philips; Aachen, Germany) and the spectrum of the light state was recorded (orange line). All measurements were performed at 25 °C.

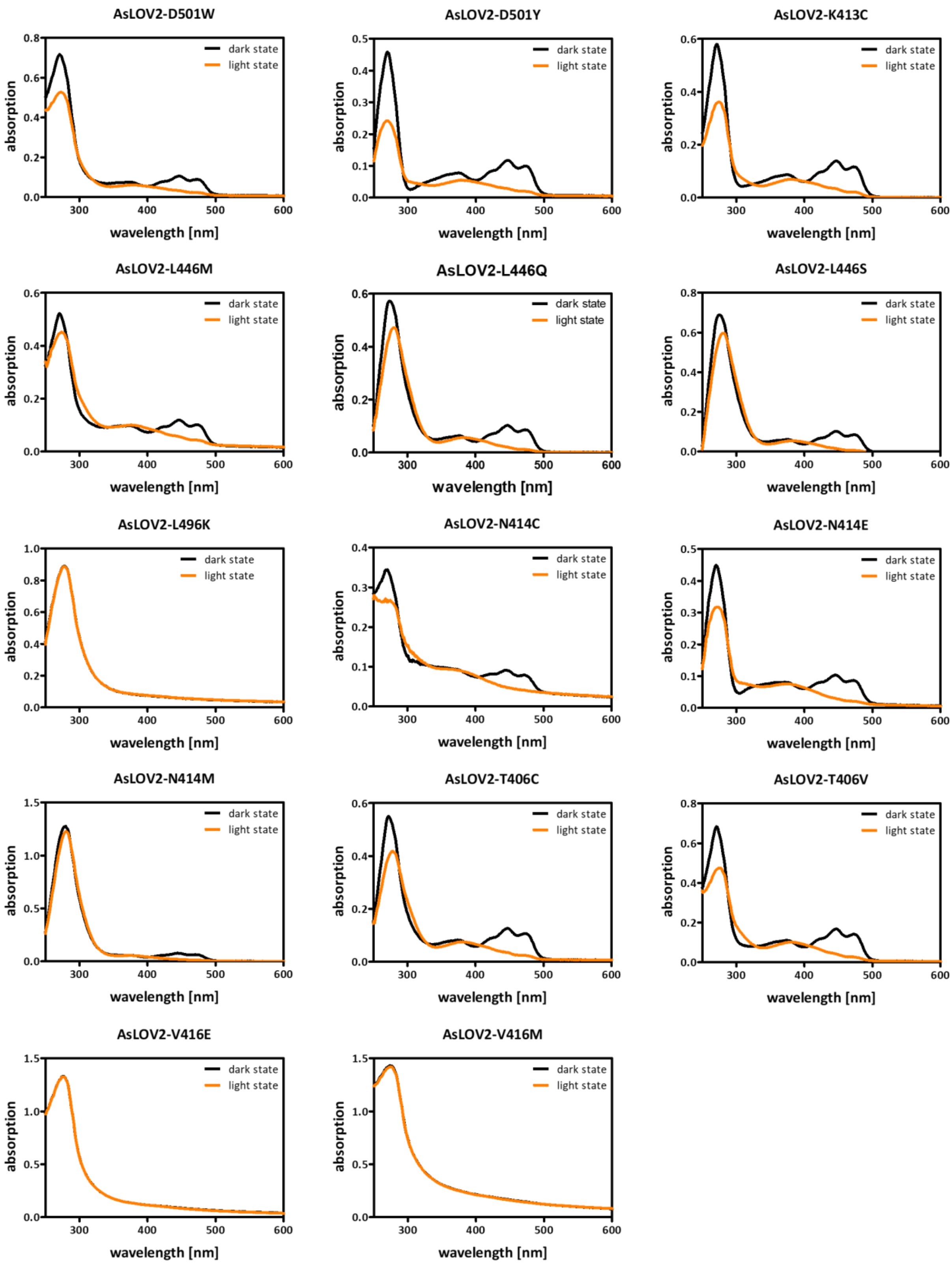

Figure S2: Absorption spectra of the dark and light states of first round selected slow single AsLOV2 variants. All samples were diluted with storage and dark recovery buffer (50 mM tris, 1 mM EDTA, 5 mM DTT; pH-value 8.0) to an OD_{450nm} of approximately 0.1 and the dark state spectrum was recorded (black line). Subsequently, the samples were illuminated for 1 min with a blue light LED (λ=440 nm, 2.6 mW/cm^2[22], Luxeon Lumileds, Philips; Aachen, Germany) and the spectrum of the light state was recorded (orange line). All measurements were performed at 25 °C.

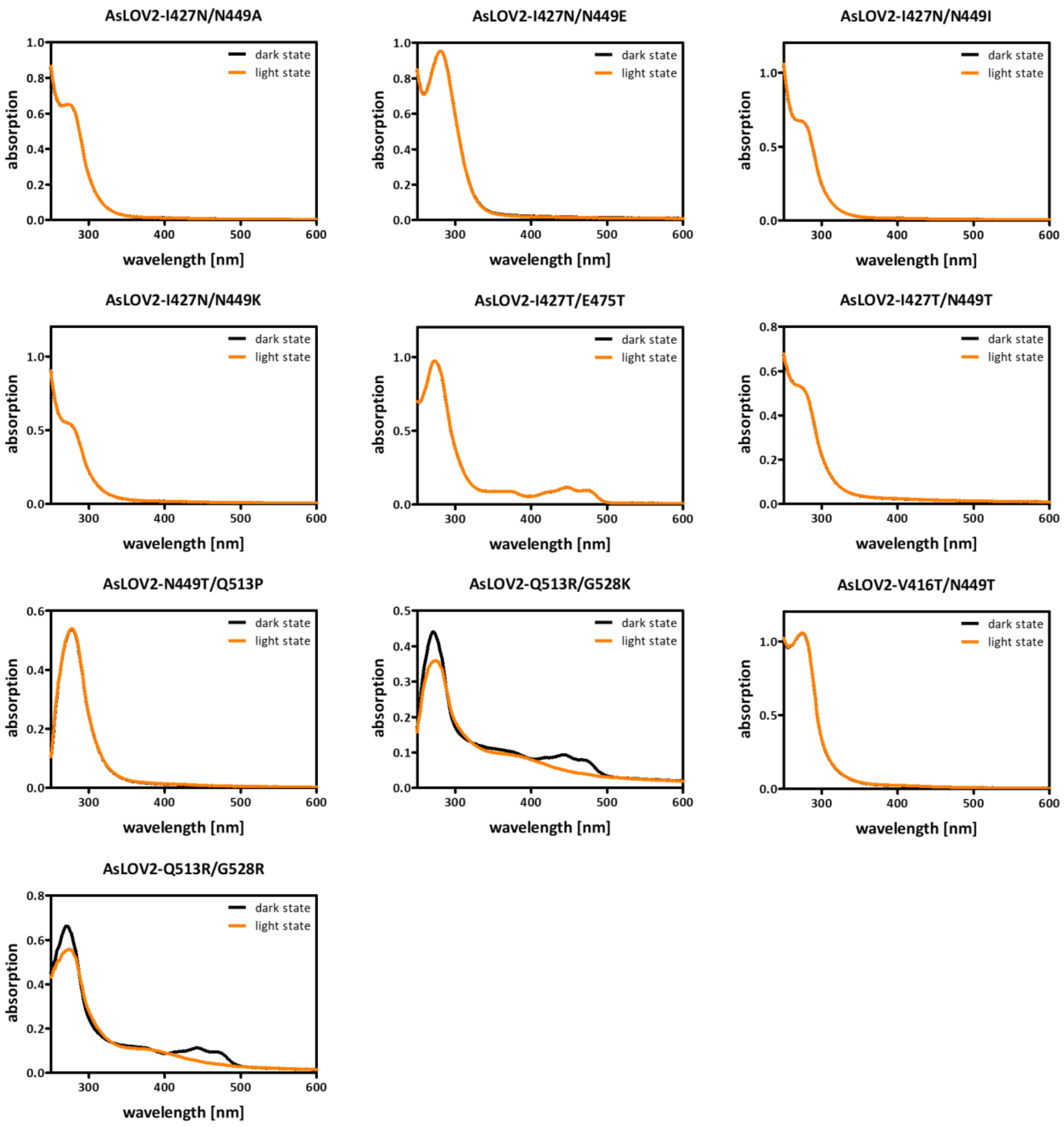

Figure S3: Absorption spectra of the dark and light states of first round ML-based predicted fast double AsLOV2 variants. All samples were diluted with storage and dark recovery buffer (50 mM tris, 1 mM EDTA, 5 mM DTT; pH-value 8.0) to an OD_{450nm} of approximately 0.1 and the dark state spectrum was recorded (black line). Subsequently, the samples were illuminated for 1 min with a blue light LED (λ=440 nm, 2.6 mW/cm^2[22], Luxeon Lumileds, Philips; Aachen, Germany) and the spectrum of the light state was recorded (orange line). All measurements were performed at 25 °C.

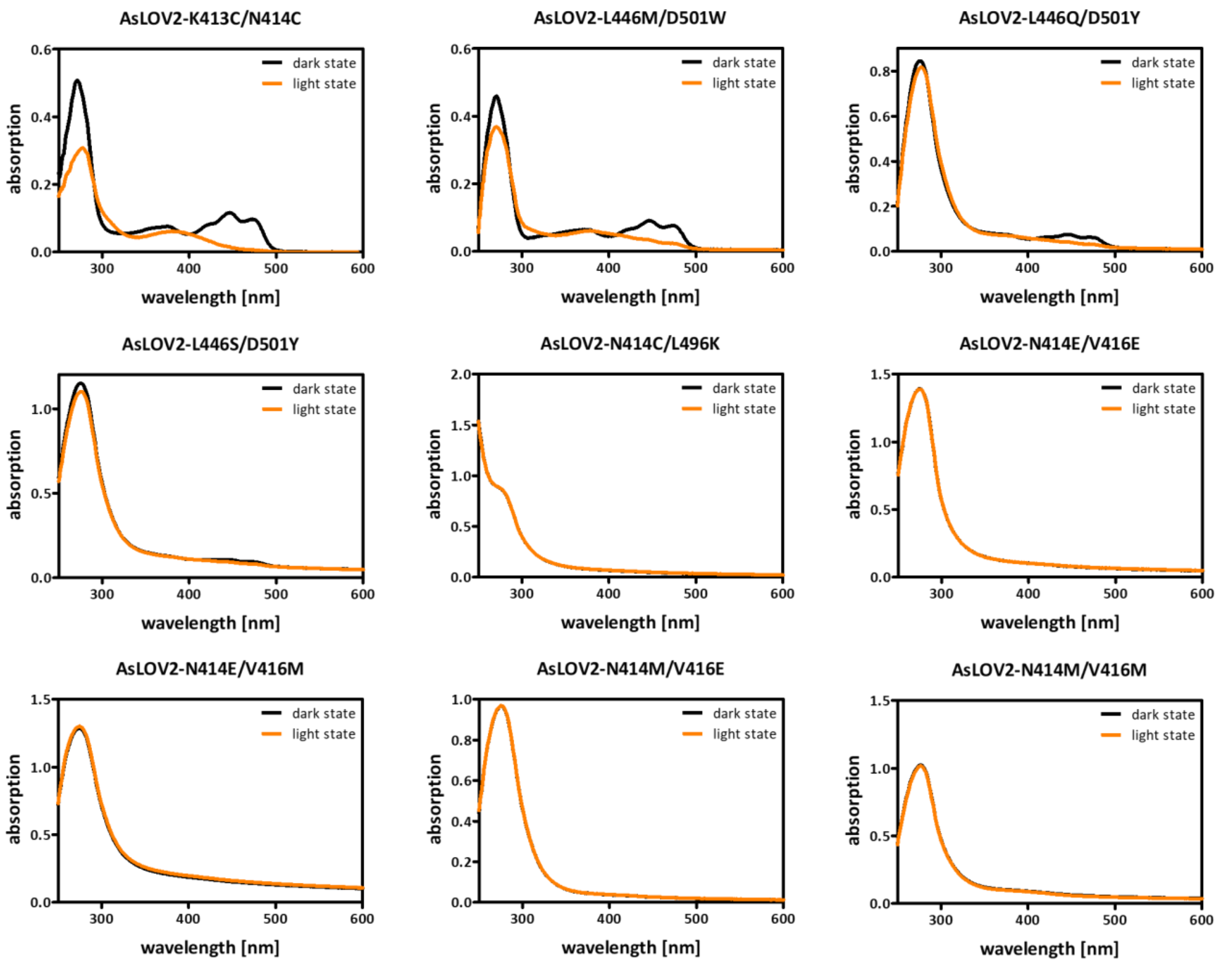

Figure S4: Absorption spectra of the dark and light states of first round ML-based predicted slow double AsLOV2 variants. All samples were diluted with storage and dark recovery buffer (50 mM tris, 1 mM EDTA, 5 mM DTT; pH-value 8.0) to an OD_{450nm} of approximately 0.1 and the dark state spectrum was recorded (black line). Subsequently, the samples were illuminated for 1 min with a blue light LED (λ=440 nm, 2.6 mW/cm^{2}[22], Luxeon Lumileds, Philips; Aachen, Germany) and the spectrum of the light state was recorded (orange line). All measurements were performed at 25 °C.

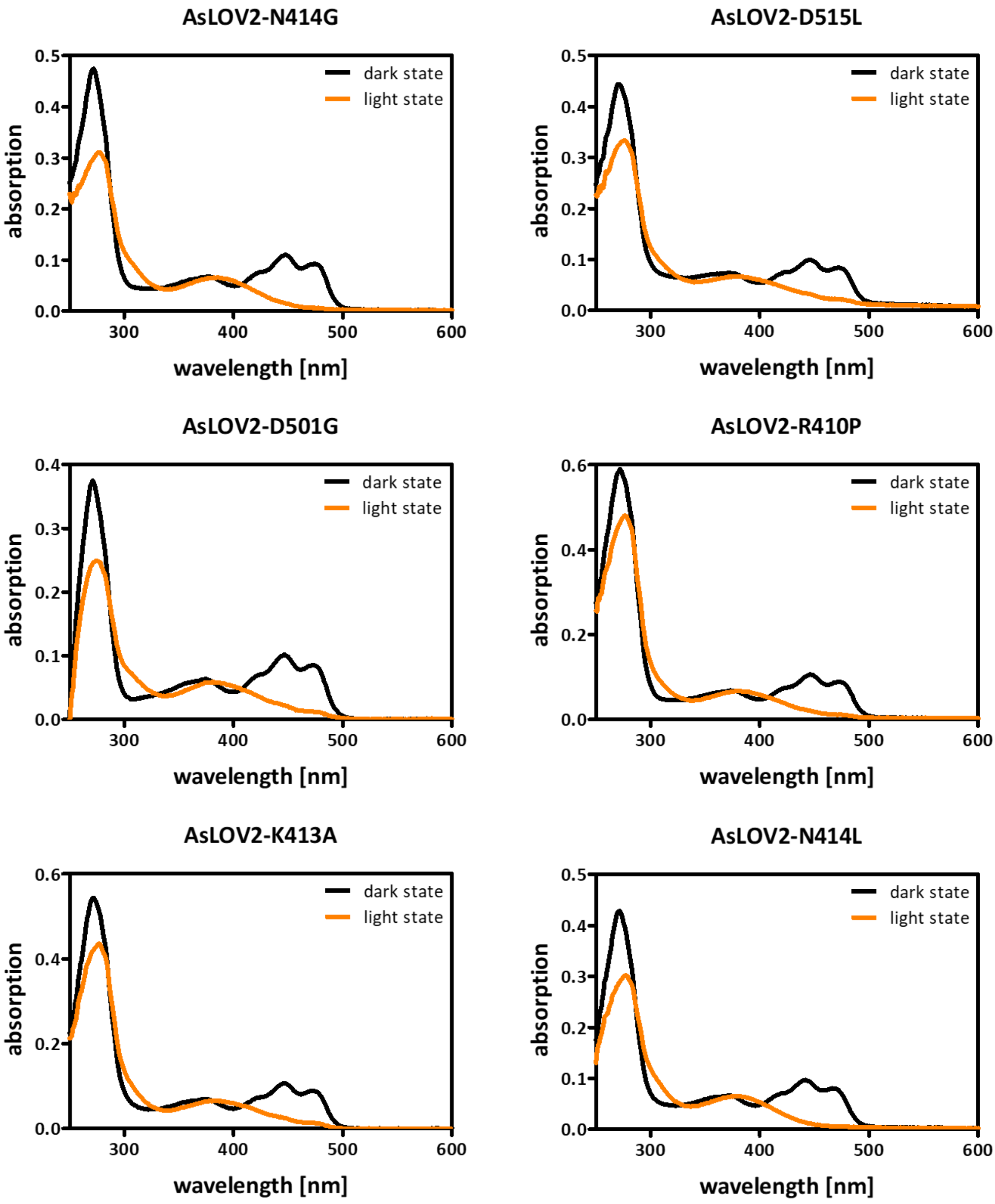

Figure S5: Absorption spectra of the dark and light states of second round selected single AsLOV2 variants. With the exception of AsLOV2-N414L, all other AsLOV2 variants were selected as fast ones. Samples were diluted with storage and dark recovery buffer (50 mM tris, 1 mM EDTA, 5 mM DTT; pH-value 8.0) to an OD_{450nm} of approximately 0.1 and the dark state spectrum was recorded (black line). Subsequently, samples were illuminated for 1 min with a blue light LED (λ=440 nm, 2.6 mW/cm^{2}[22], Luxeon Lumileds, Philips; Aachen, Germany) and the spectrum of the light state was recorded (orange line). All measurements were performed at 25 °C.

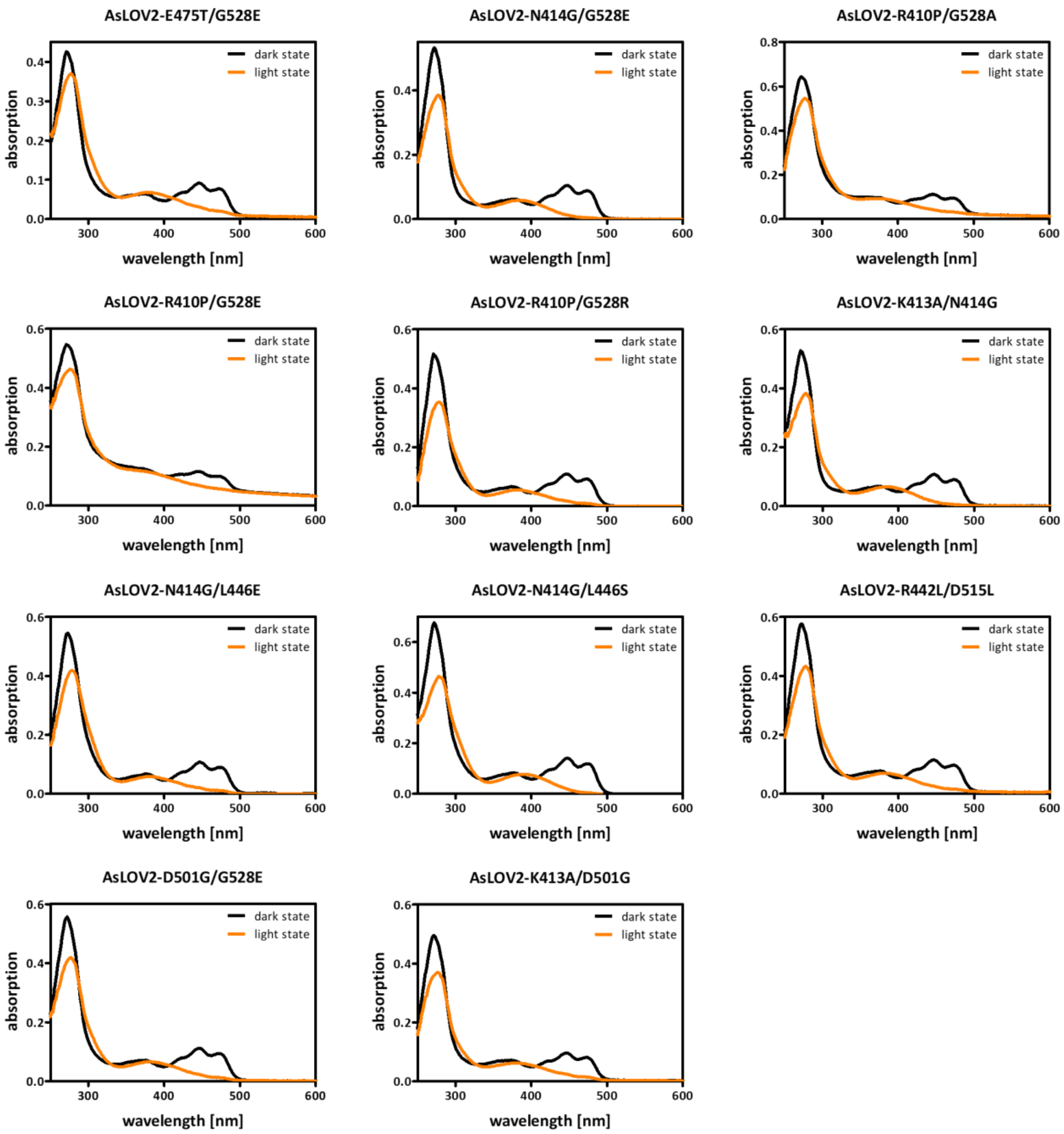

Figure S6: Absorption spectra of the dark and light states of second round ML-based predicted fast double AsLOV2 variants. All samples were diluted with storage and dark recovery buffer (50 mM tris, 1 mM EDTA, 5 mM DTT; pH-value 8.0) to an OD_{450nm} of approximately 0.1 and the dark state spectrum was recorded (black line). Subsequently, the samples were illuminated for 1 min with a blue light LED (λ=440 nm, 2.6 mW/cm^2[22], Luxeon Lumileds, Philips; Aachen, Germany) and the spectrum of the light state was recorded (orange line). All measurements were performed at 25 °C.

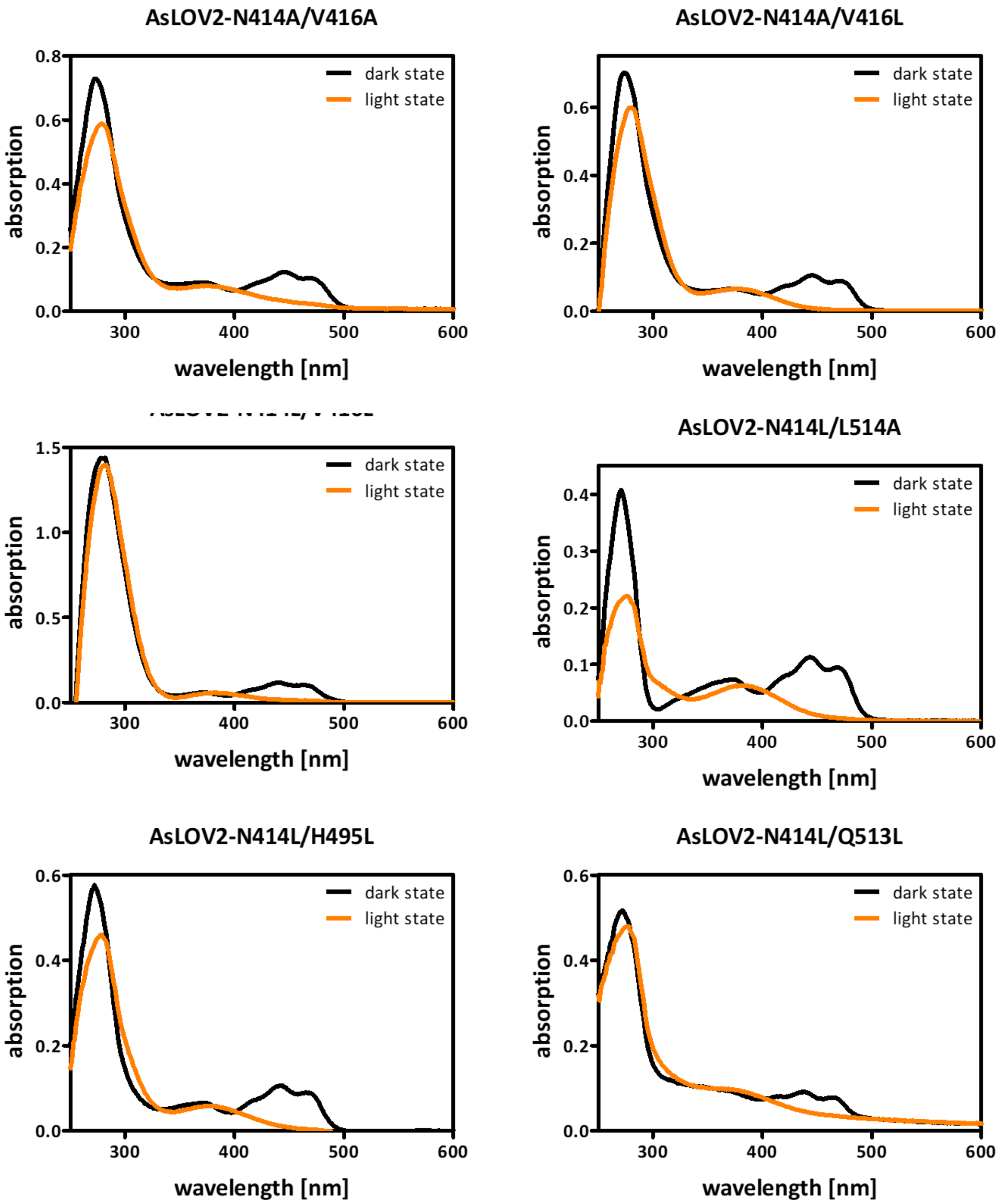

Figure S7: Absorption spectra of the dark and light states of second round ML-based predicted slow double AsLOV2 variants. All samples were diluted with storage and dark recovery buffer (50 mM tris, 1 mM EDTA, 5 mM DTT; pH-value 8.0) to an OD_{450nm} of approximately 0.1 and the dark state spectrum was recorded (black line). Subsequently, the samples were illuminated for 1 min with a blue light LED (λ=440 nm, 2.6 mW/cm^2[22], Luxeon Lumileds, Philips; Aachen, Germany) and the spectrum of the light state was recorded (orange line). All measurements were performed at 25 °C.

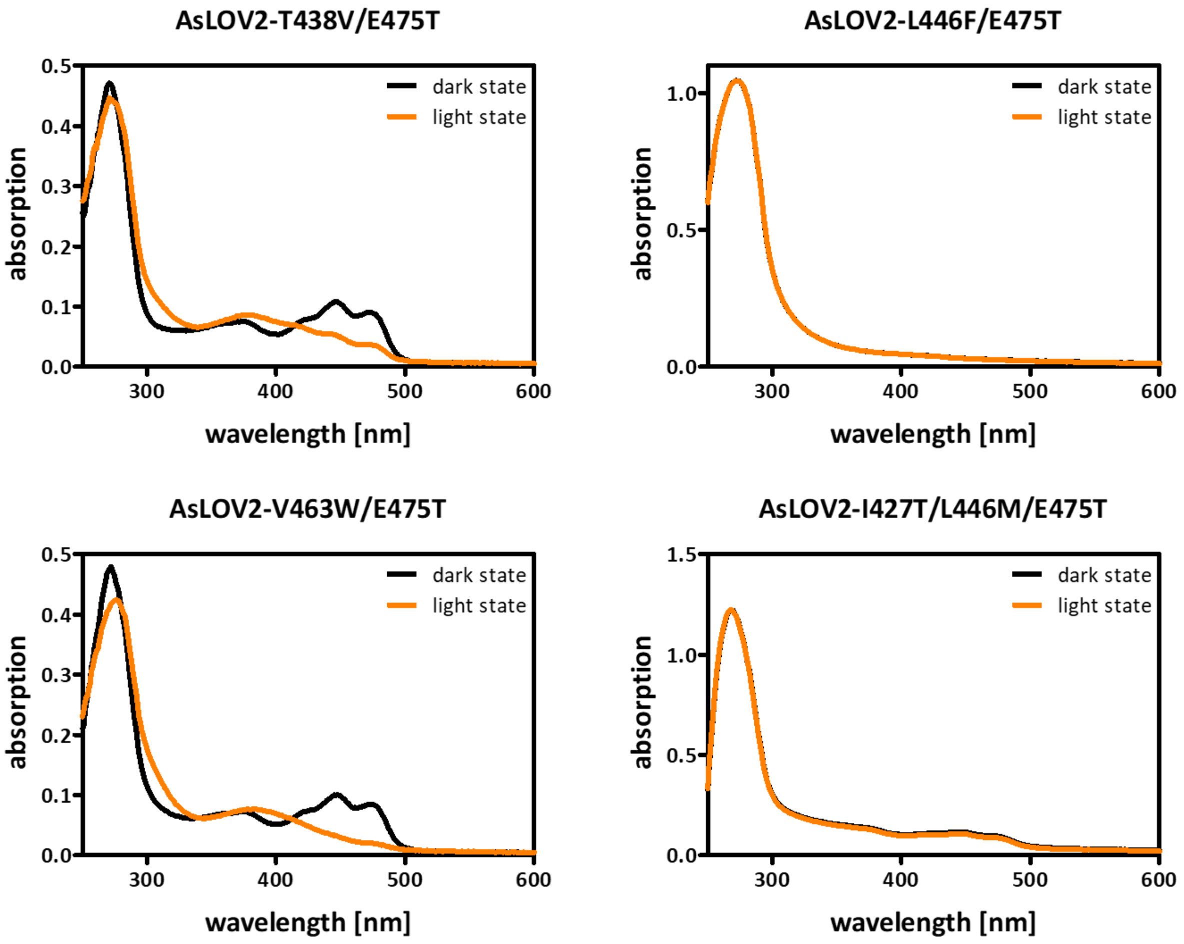

Figure S8: Absorption spectra of the dark and light states of third round ML-based predicted fast double and triple AsLOV2 variants. All samples were diluted with storage and dark recovery buffer (50 mM tris, 1 mM EDTA, 5 mM DTT; pH-value 8.0) to an OD_{450nm} of approximately 0.1 and the dark state spectrum was recorded (black line). Subsequently, the samples were illuminated for 1 min with a blue light LED (λ=440 nm, 2.6 mW/cm^2[22], Luxeon Lumileds, Philips; Aachen, Germany) and the spectrum of the light state was recorded (orange line). All measurements were performed at 25 °C.

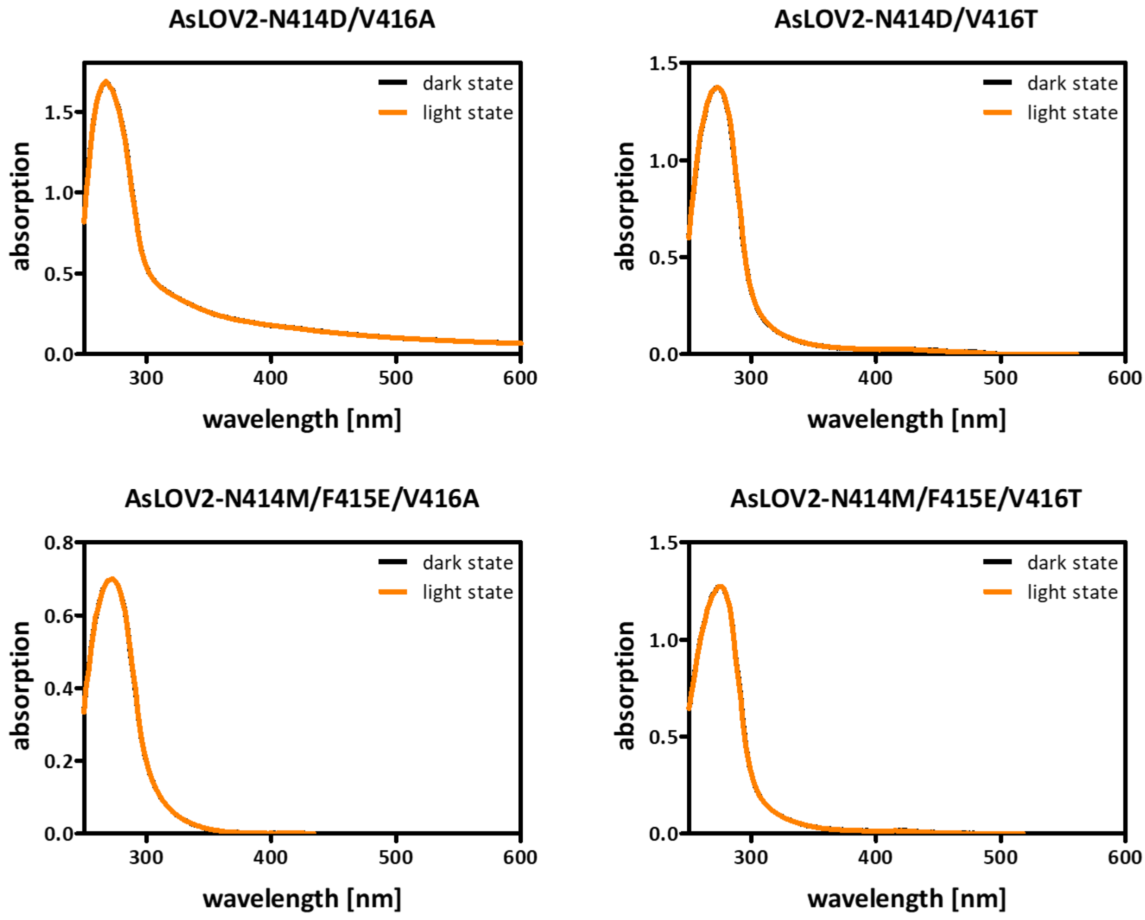

Figure S9: Absorption spectra of the dark and light states of third round ML-based predicted slow double and triple AsLOV2 variants. All samples were diluted with storage and dark recovery buffer (50 mM tris, 1 mM EDTA, 5 mM DTT; pH-value 8.0) to an OD_{450nm} of approximately 0.1 and the dark state spectrum was recorded (black line). Subsequently, the samples were illuminated for 1 min with a blue light LED (λ=440 nm, 2.6 mW/cm^2[22], Luxeon Lumileds, Philips; Aachen, Germany) and the spectrum of the light state was recorded (orange line). All measurements were performed at 25 °C.

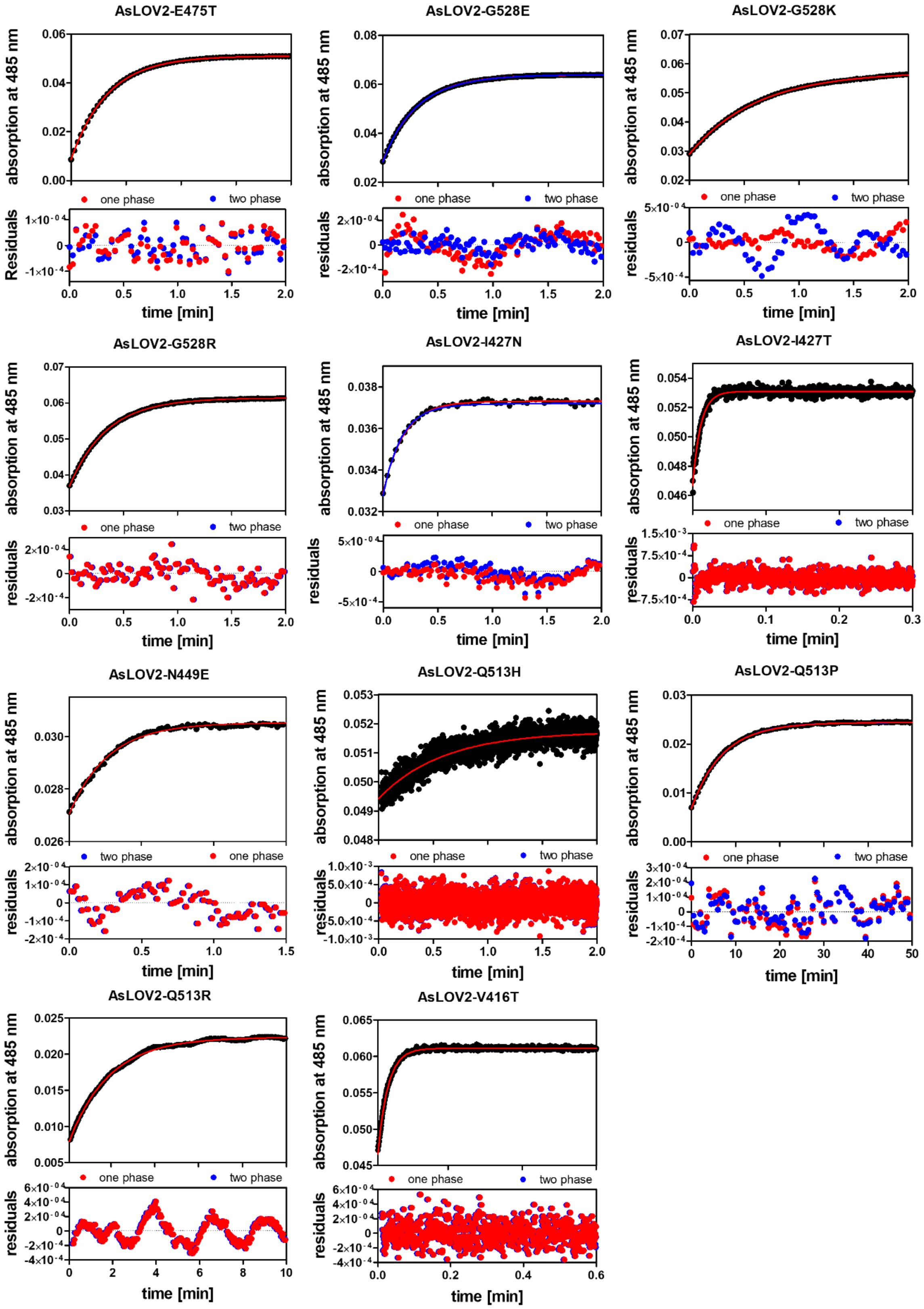

Figure S10: Dark recovery time traces (top graph of each panel) and residual plots (lower graph) of first round selected fast single AsLOV2 variants. Absorbance at λ=485 nm was plotted against time after illumination of the sample. Only one exemplary recovery time trace out of a set of triplicate measurements is shown. The adduct state life τ_{FMN} was obtained time by fitting the experimental data (black) to one phase (red line) or two phase (blue line) exponential decay function. Data shown in residual plots is coloured accordingly. All measurements were performed at 25 °C.

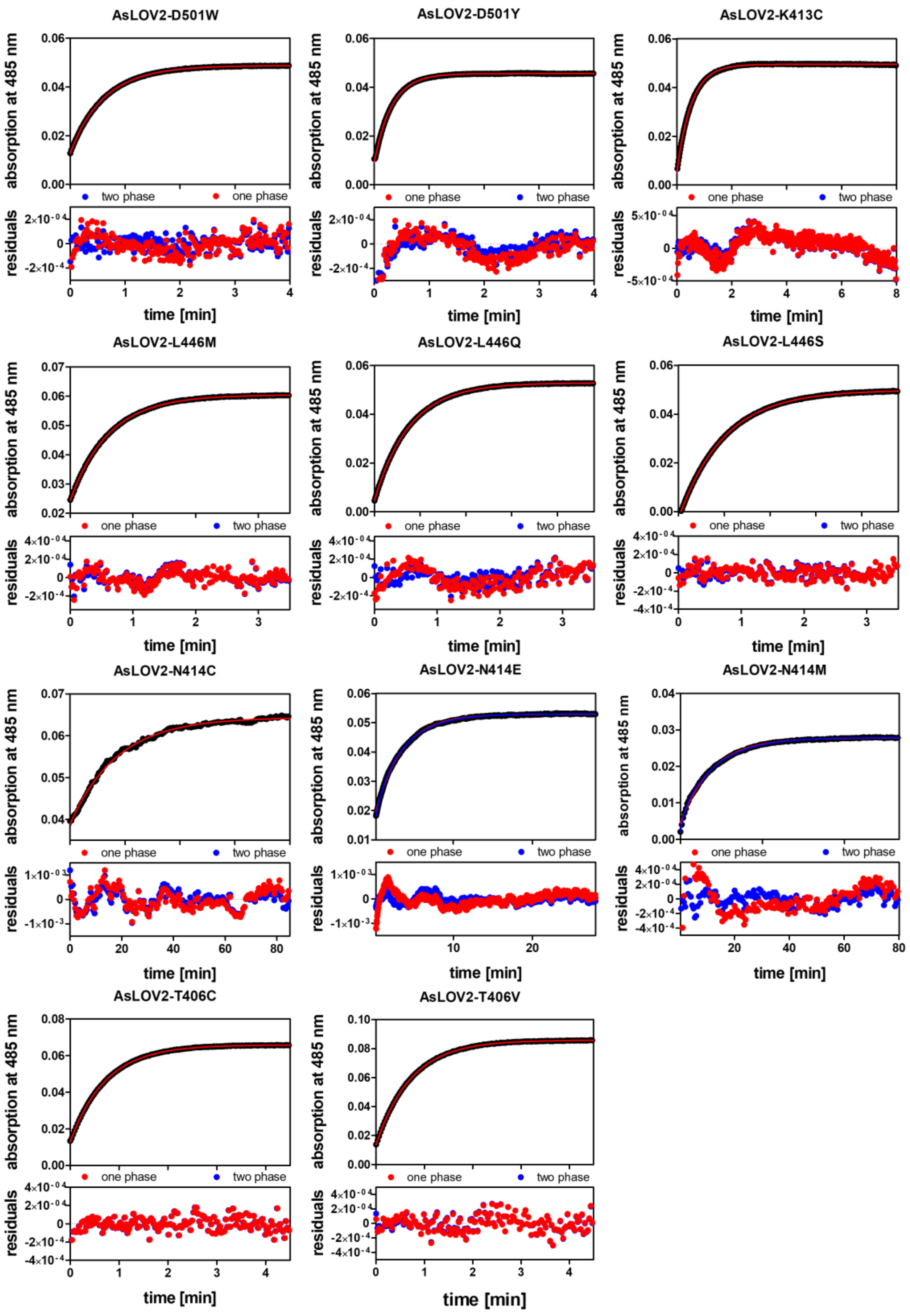

Figure S11: Dark recovery time traces (top graph of each panel) and residual plots (lower graph) of first round selected slow single AsLOV2 variants. Absorbance at λ=485 nm was plotted against time after illumination of the sample. Only one exemplary recovery time trace out of a set of triplicate measurements is shown. The adduct state life τ_{FMN} was obtained time by fitting the experimental data (black) to one phase (red line) or two phase (blue line) exponential decay function. Data shown in residual plots is coloured accordingly. All measurements were performed at 25 °C.

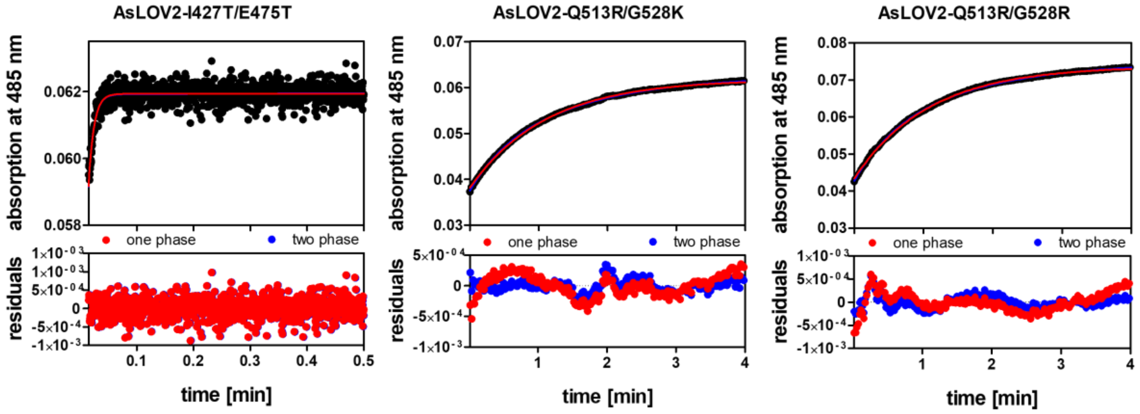

Figure S12: Dark recovery time traces (top graph of each panel) and residual plots (lower graph) of first round ML- predicted fast double AsLOV2 variants. Absorbance at λ=485 nm was plotted against time after illumination of the sample. Only one exemplary recovery time trace out of a set of triplicate measurements is shown. The adduct state life τ_{FMN} was obtained time by fitting the experimental data (black) to one phase (red line) or two phase (blue line) exponential decay function. Data shown in residual plots is coloured accordingly. All measurements were performed at 25 °C.

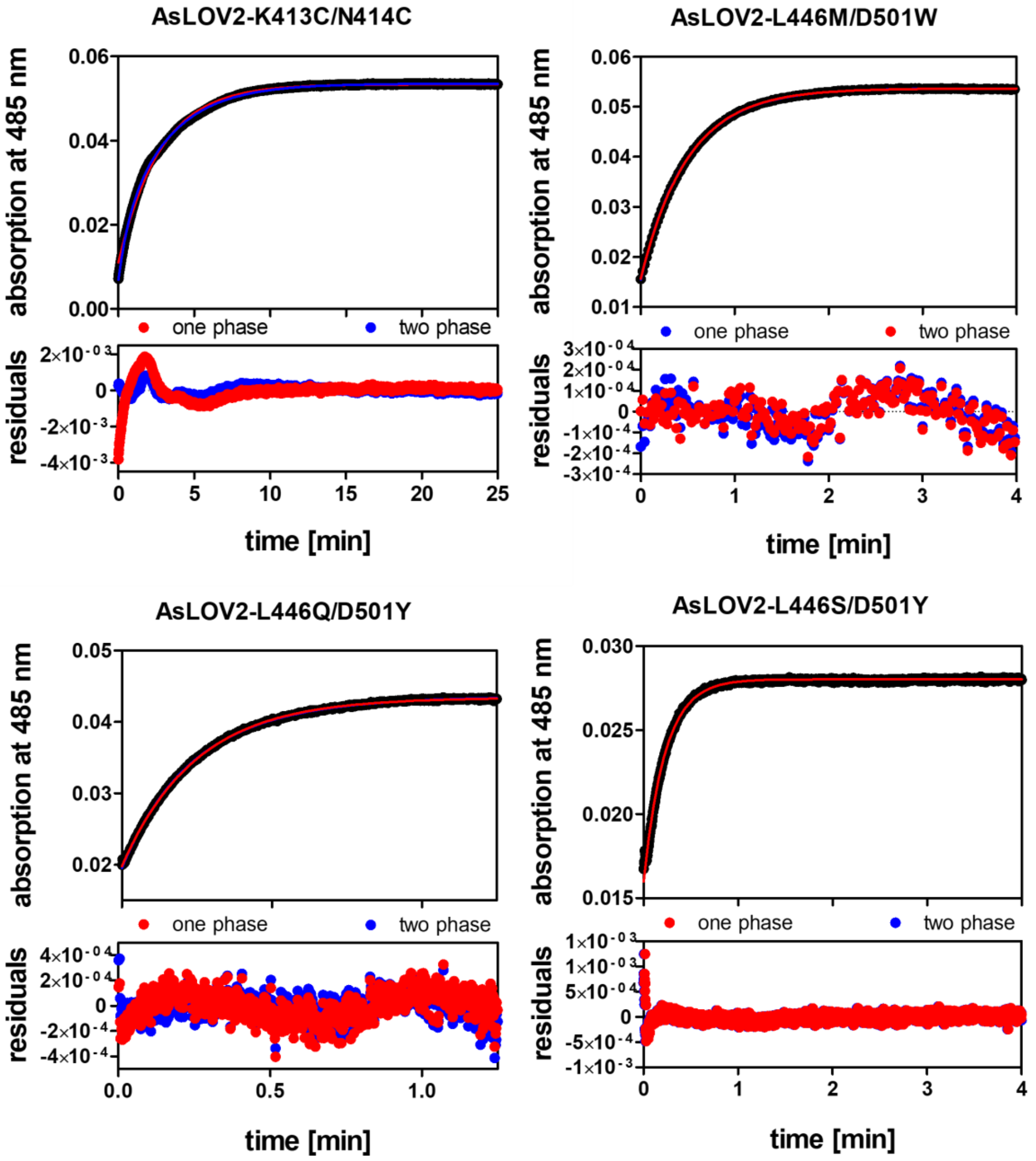

Figure S13: Dark recovery time traces (top graph of each panel) and residual plots (lower graph) of first round ML- predicted slow double AsLOV2 variants. Absorbance at λ=485 nm was plotted against time after illumination of the sample. Only one exemplary recovery time trace out of a set of triplicate measurements is shown. The adduct state life τ_{FMN} was obtained time by fitting the experimental data (black) to one phase (red line) or two phase (blue line) exponential decay function. Data shown in residual plots is coloured accordingly. All measurements were performed at 25 °C.

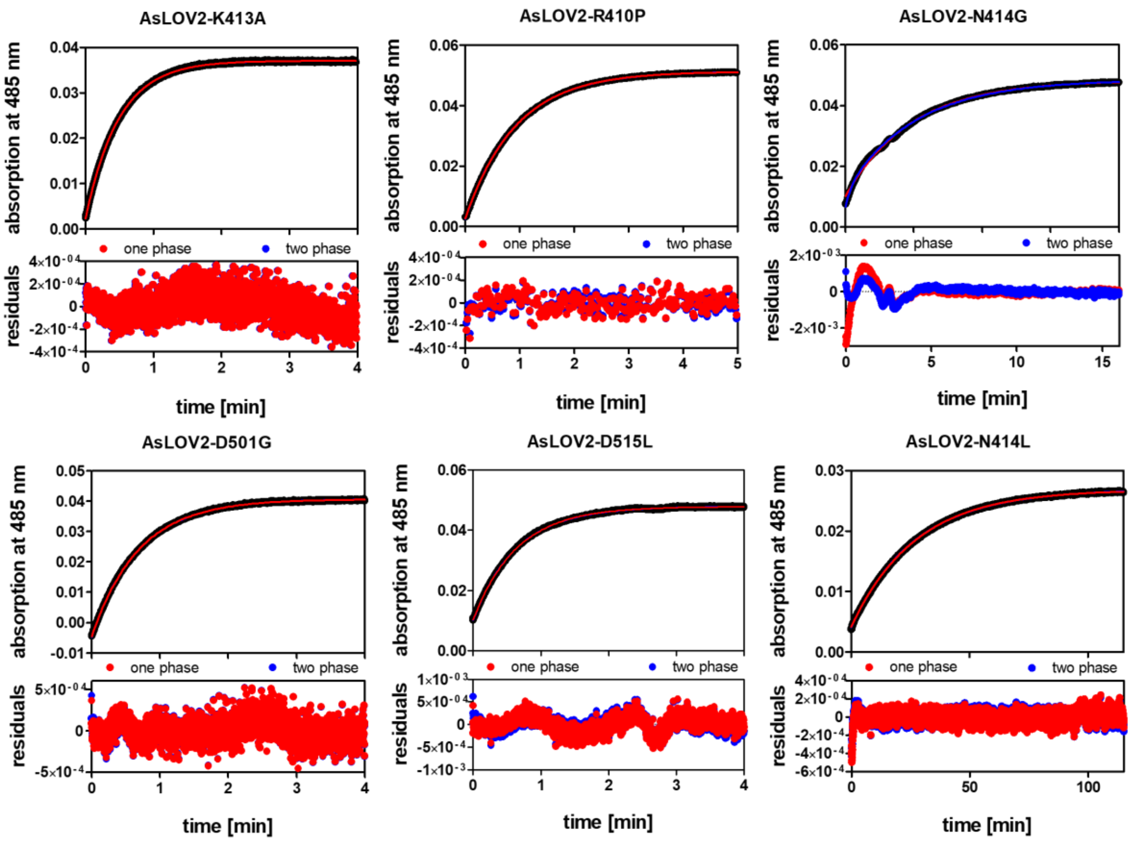

Figure S14: Dark recovery time traces (top graph of each panel) and residual plots (lower graph) of second round selected single AsLOV2 variants. With the exception of AsLOV2-N414L, all other AsLOV2 variants were selected as fast ones. Absorbance at λ=485 nm was plotted against time after illumination of the sample. Only one exemplary recovery time trace out of a set of triplicate measurements is shown. The adduct state life τ_{FMN} was obtained time by fitting the experimental data (black) to one phase (red line) or two phase (blue line) exponential decay function. Data shown in residual plots is coloured accordingly. All measurements were performed at 25 °C.

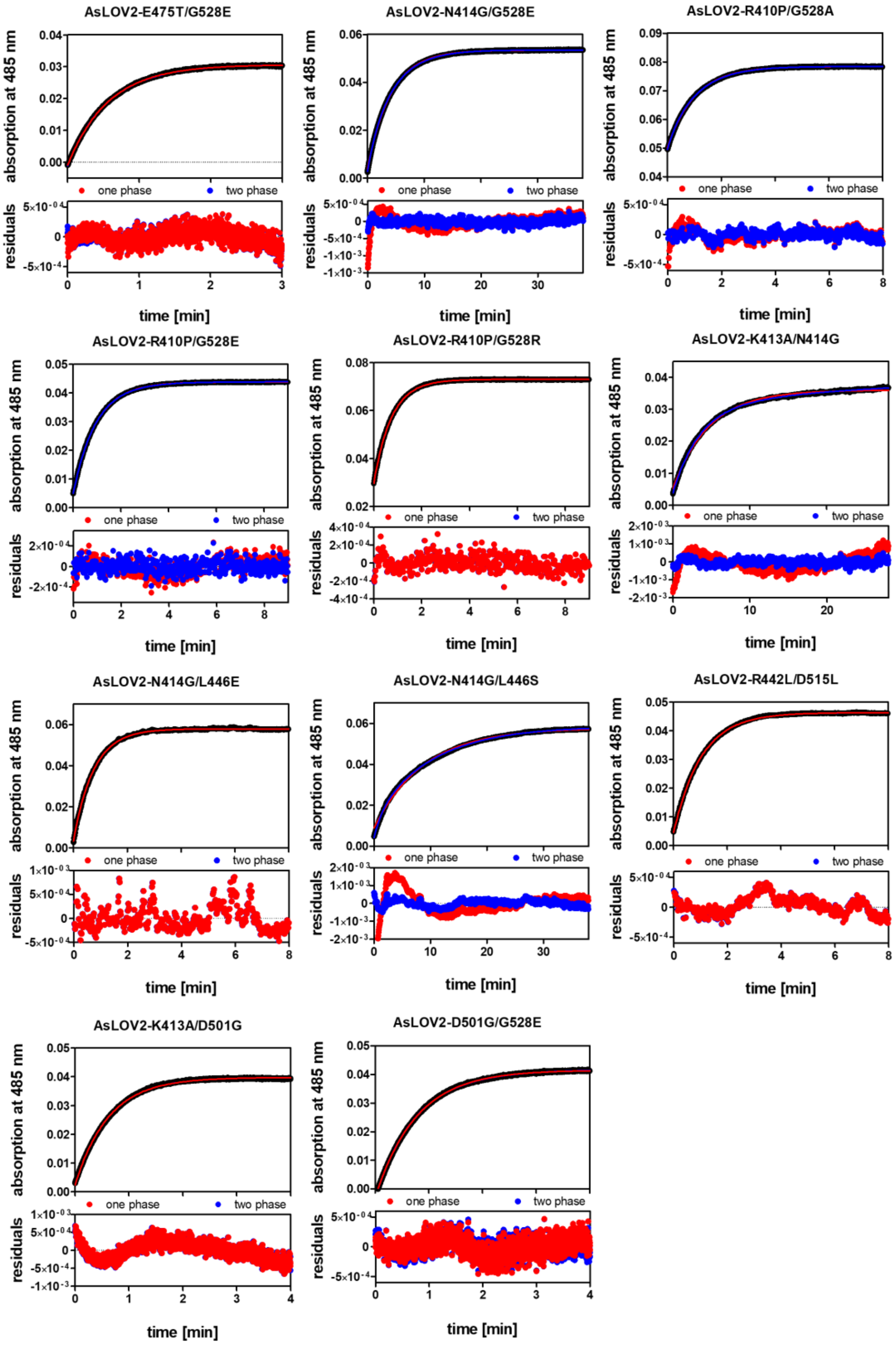

Figure S15: Dark recovery time traces (top graph of each panel) and residual plots (lower graph) of second round ML-predicted fast double AsLOV2 variants. Absorbance at λ=485 nm was plotted against time after illumination of the sample. Only one exemplary recovery time trace out of a set of triplicate measurements is shown. The adduct state life τ_{FMN} was obtained time by fitting the experimental data (black) to one phase (red line) or two phase (blue line) exponential decay function. Data shown in residual plots is coloured accordingly. All measurements were performed at 25 °C.

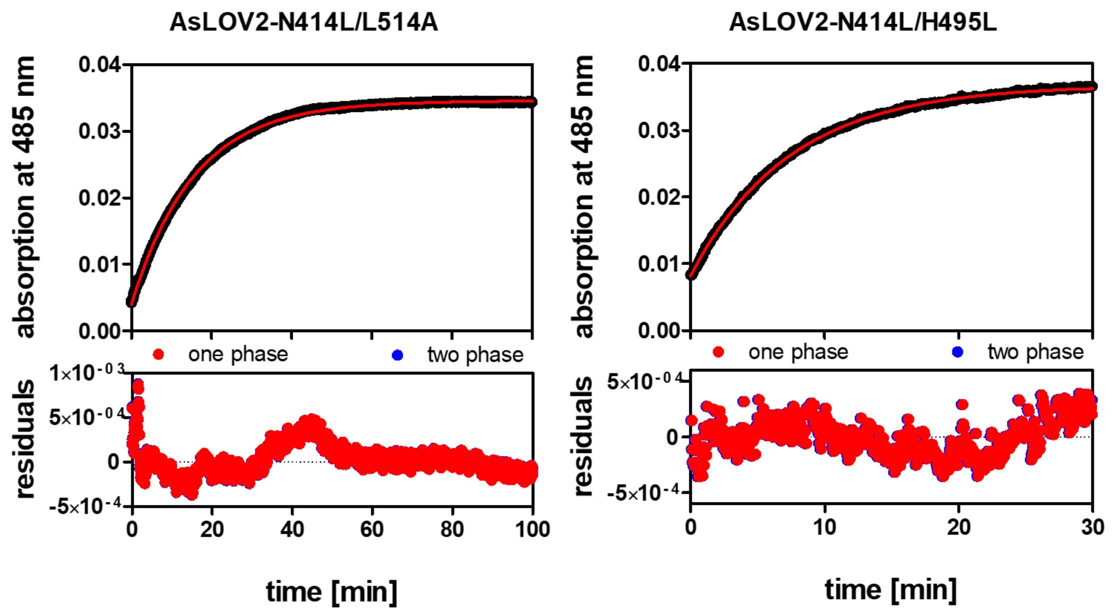

Figure S16: Dark recovery time traces (top graph of each panel) and residual plots (lower graph) of second round ML-predicted slow double AsLOV2 variants. Absorbance at λ=485 nm was plotted against time after illumination of the sample. Only one exemplary recovery time trace out of a set of triplicate measurements is shown. The adduct state life τ_{FMN} was obtained time by fitting the experimental data (black) to one phase (red line) or two phase (blue line) exponential decay function. Data shown in residual plots is coloured accordingly. All measurements were performed at 25 °C.

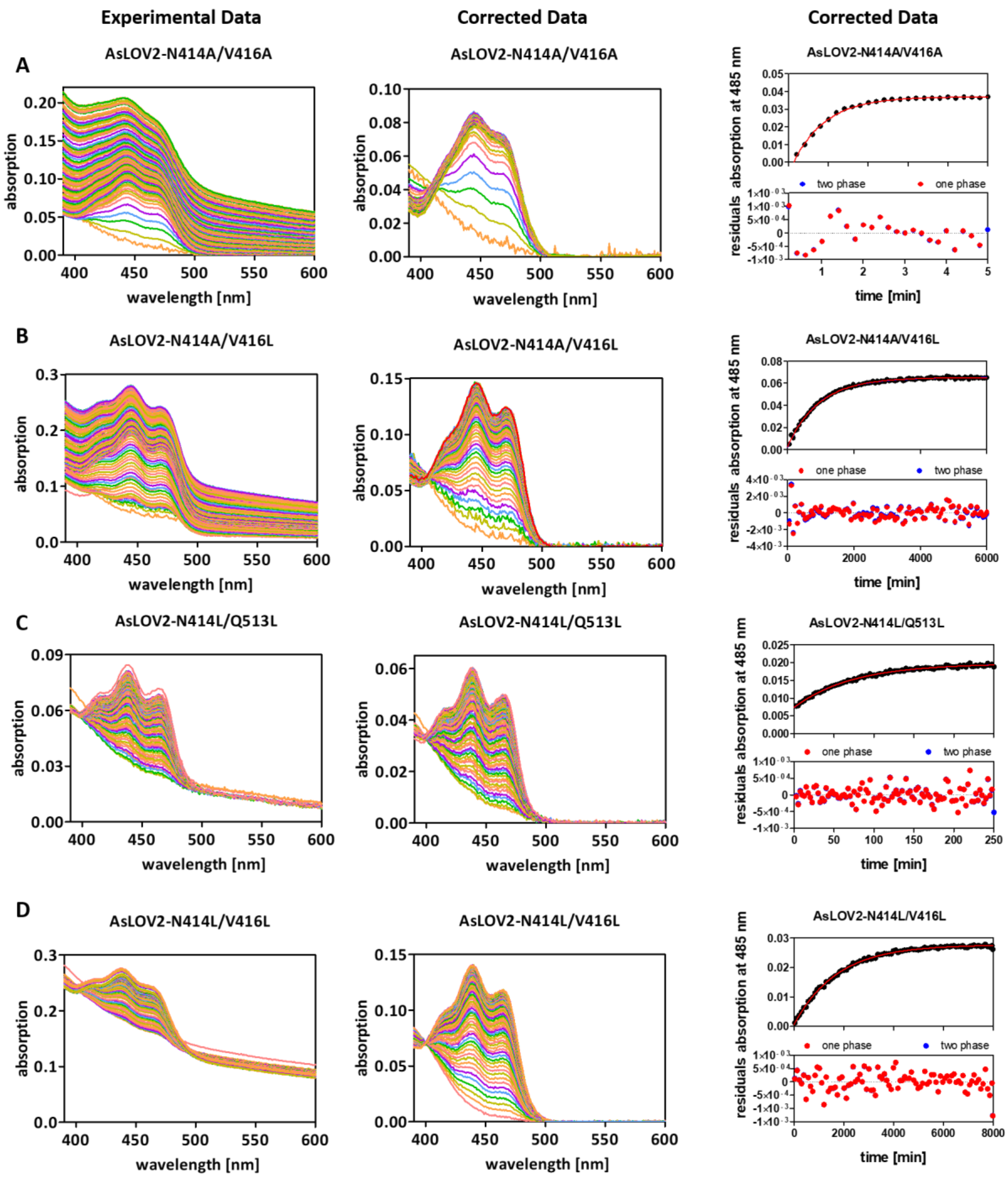

Figure S17: UV/Vis spectra recorded during the dark recovery of a subset of aggregation-prone AsLOV2 variants or of variants showing a very slow recovery. Shown are absorbance spectra of the respective protein sample taken during the dark recovery of the protein after illumination. For each protein, the uncorrected (left) and the scatter-corrected (middle) UV/Vis spectra are shown. The right-most panels depict dark recovery time traces (top graph of each panel) and residual plots (lower graph). Absorbance at λ=485 nm was plotted against time after illumination of the sample. Only one exemplary recovery time trace out of a set of triplicate measurements is shown. The adduct state life τ_{FMN} was obtained time by fitting the experimental data (black) to one phase (red line) or two phase (blue line) exponential decay function. Data shown in residual plots is coloured accordingly. All measurements were performed at 25 °C.

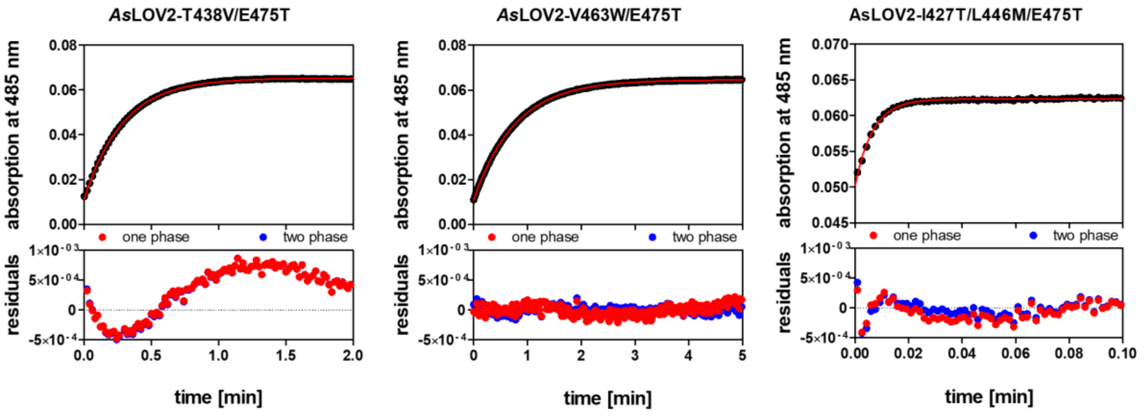

Figure S18: Dark recovery time traces (top graph of each panel) and residual plots (lower graph) of third round ML-based predicted fast double and triple AsLOV2 variants. Absorbance at λ=485 nm was plotted against time after illumination of the sample. Only one exemplary recovery time trace out of a set of triplicate measurements is shown. The adduct state life τ_{FMN} was obtained time by fitting the experimental data (black) to one phase (red line) or two phase (blue line) exponential decay function. Data shown in residual plots is coloured accordingly. All measurements were performed at 25 °C.

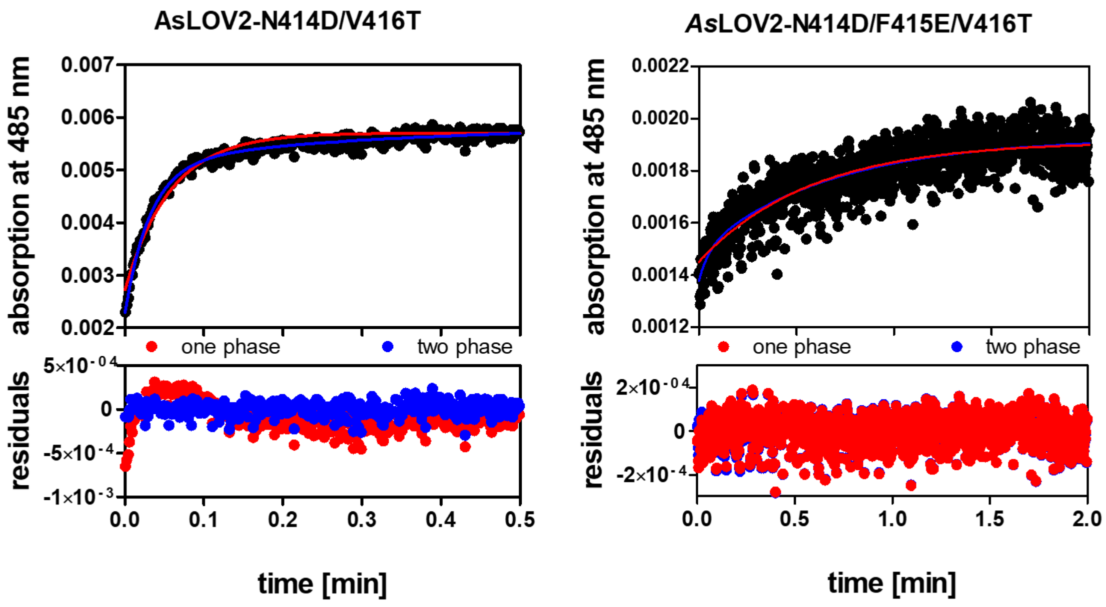

Figure S19: Dark recovery time traces (top graph of each panel) and residual plots (lower graph) of third round ML-predicted slow double and triple AsLOV2 variants. Absorbance at λ=485 nm was plotted against time after illumination of the sample. Only one exemplary recovery time trace out of a set of triplicate measurements is shown. The adduct state life τ_{FMN} was obtained time by fitting the experimental data (black) to one phase (red line) or two phase (blue line) exponential decay function. Data shown in residual plots is coloured accordingly. All measurements were performed at 25 °C.

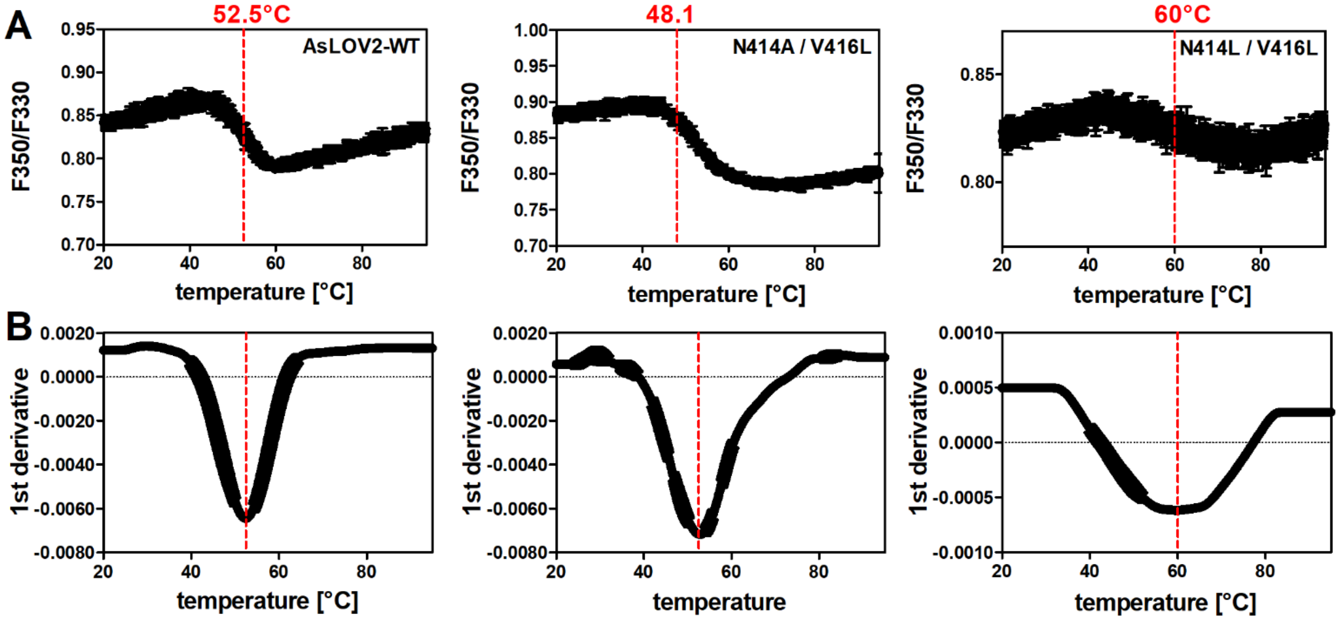

Figure S20: Thermal stability of AsLOV2 wild type and selected slow cycling AsLOV2 variants. A) Plot of the F350/F330 fluorescence ratio versus temperature (temperature ramp 1°C per minute) B) First derivative of the F350/F330 ratio, which yields the melting temperature T_m as the transition midpoint (depicted as red dashed line in both panels and given above panel A). Protein concentration 0.4-0.5 mg mL^{-1}

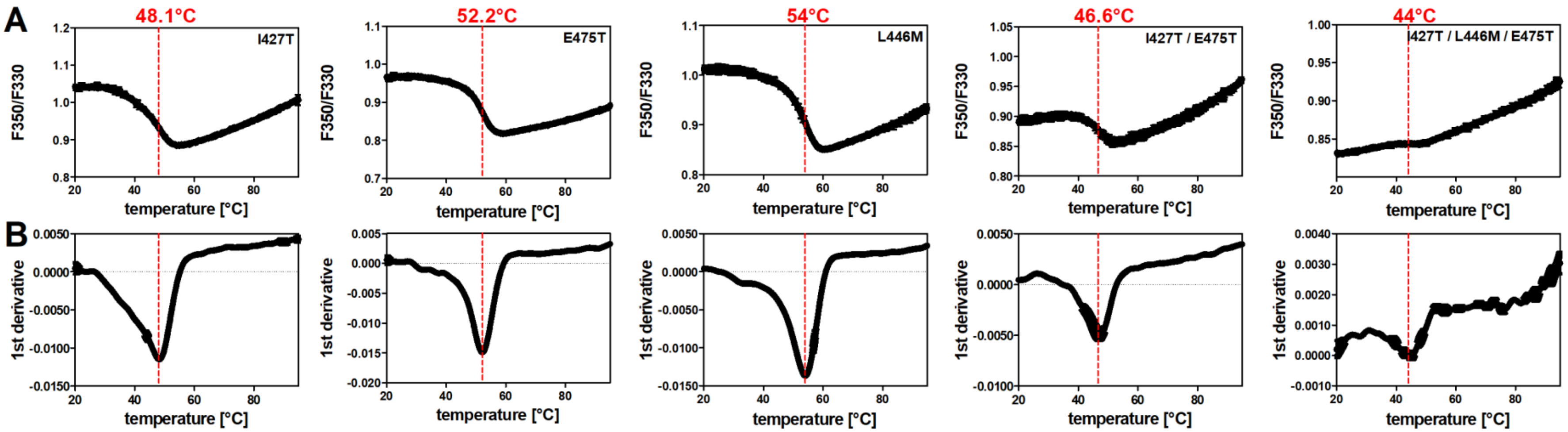

Figure S21: Thermal stability of selected fast cycling AsLOV2 variants. A) Plot of the F350/F330 fluorescence ratio versus temperature (temperature ramp 1°C per minute) B) First derivative of the F350/F330 ratio, which yields the melting temperature T_m as the transition midpoint (depicted as red dashed line in both panels and given above panel A). Protein concentration 0.4-0.5 mg mL^{-1}

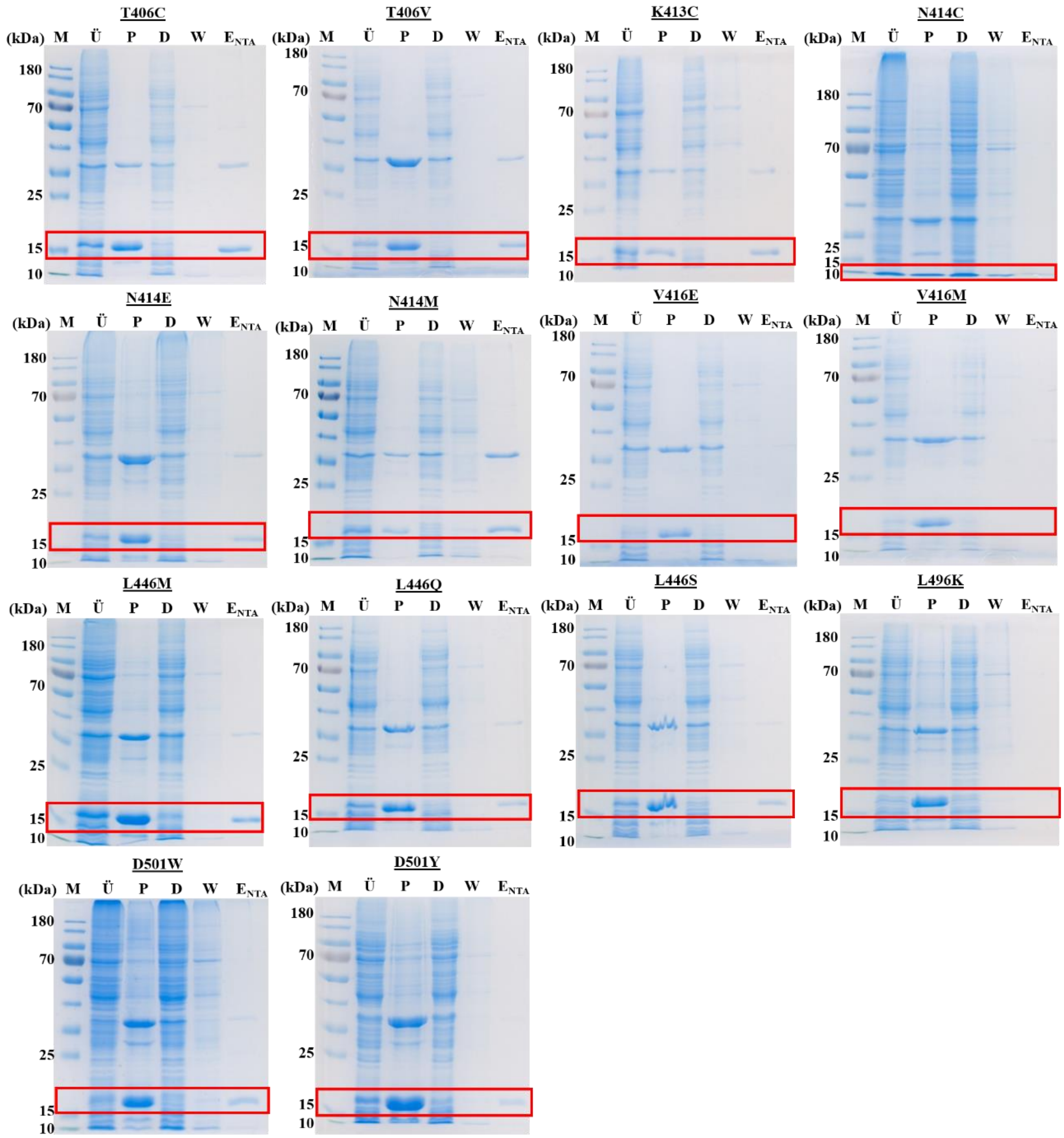

Figure S22: SDS-PAGE analysis of AsLOV2 variants generated, purified and characterized in this study. Each gel depicts the purification progress during Ni-NTA based immobilized metal ion affinity chromatography (IMAC), with lanes label with M: the marker protein mix (PageRuler, pre-stained protein ladder, Thermo Fisher Scientific, Waltham, MA USA), Ü: representing the soluble fraction of the crude cell lysate, P: the insoluble pellet fraction of the crude cell lysate, D: the flow-through of the IMAC column after loading, W: the wash fraction and E_{NTA} the purified protein after IMAC. Relevant marker bands are label to allow estimation of protein molecular weights. AsLOV2 has a theoretical molecular weight of about 18 kDa (marked by red boxes in all gel pictures)

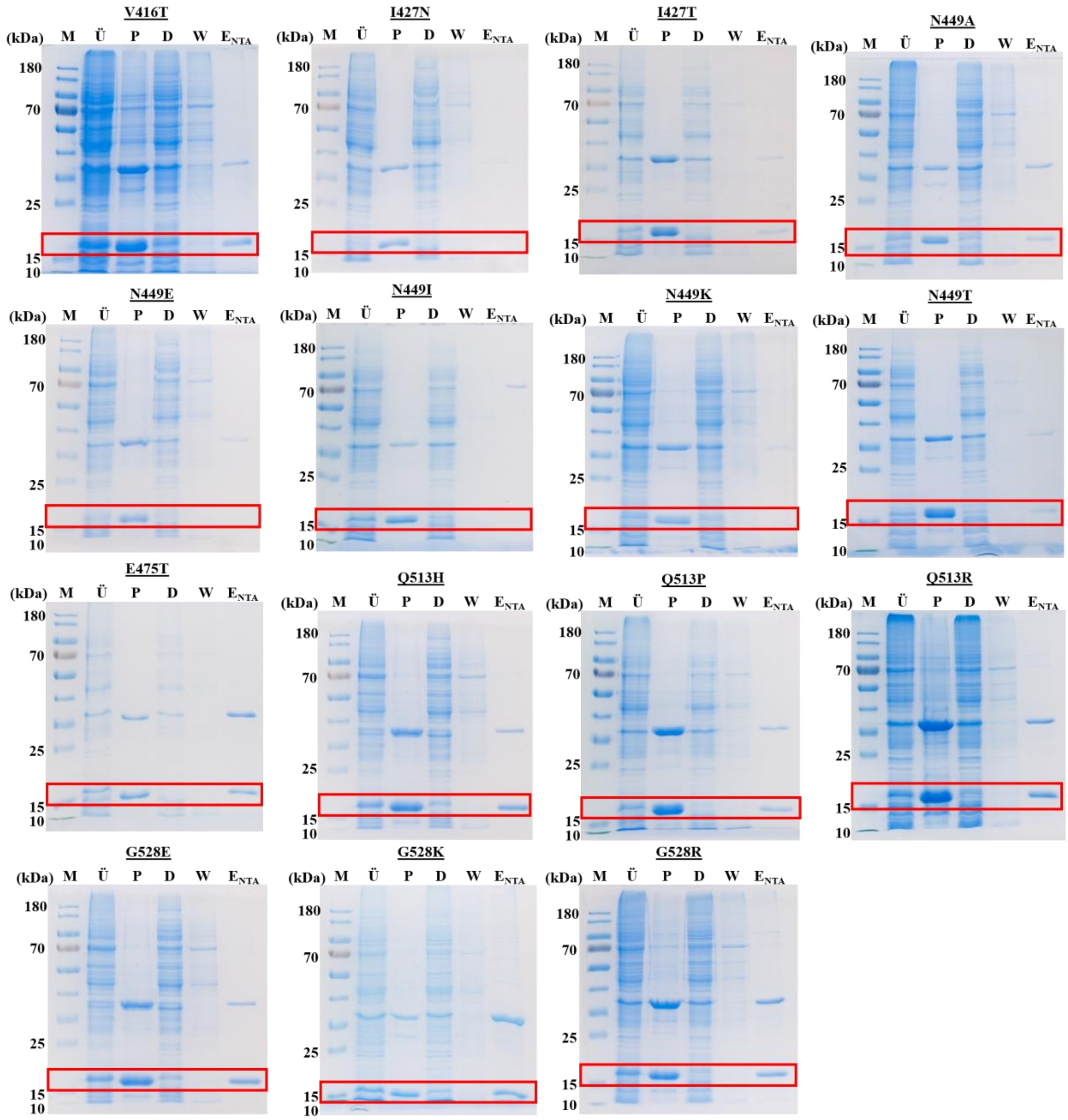

Figure S23: SDS-PAGE analysis of AsLOV2 variants generated, purified and characterized in this study. Each gel depicts the purification progress during Ni-NTA based immobilized metal ion affinity chromatography (IMAC), with lanes label with M: the marker protein mix (PageRuler, pre-stained protein ladder, Thermo Fisher Scientific, Waltham, MA USA), Ü: representing the soluble fraction of the crude cell lysate, P: the insoluble pellet fraction of the crude cell lysate, D: the flow-through of the IMAC column after loading, W: the wash fraction and E_{NTA} the purified protein after IMAC. Relevant marker bands are label to allow estimation of protein molecular weights. AsLOV2 has a theoretical molecular weight of about 18 kDa (marked by red boxes in all gel pictures)

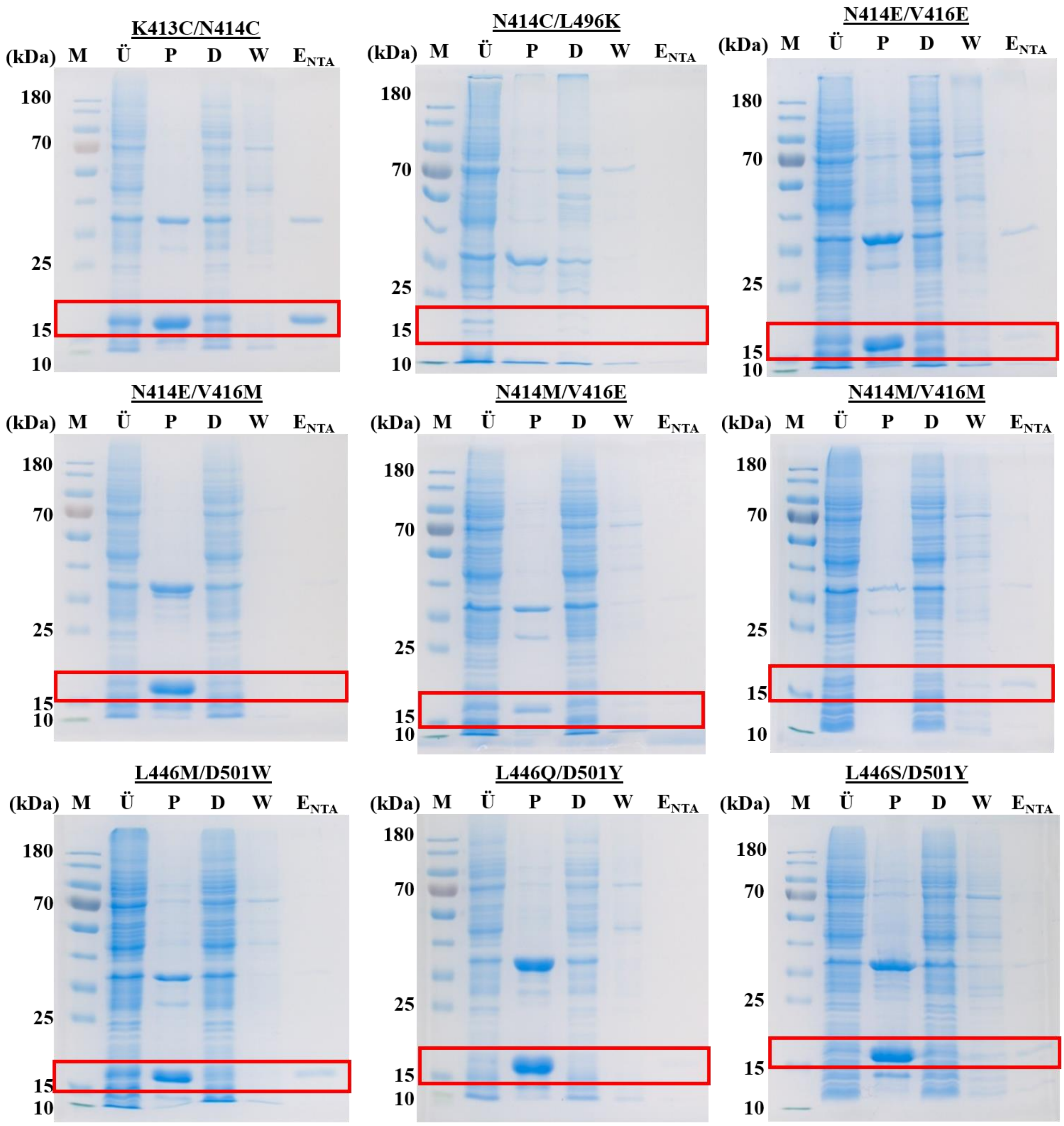

Figure S24: SDS-PAGE analysis of AsLOV2 variants generated, purified and characterized in this study. Each gel depicts the purification progress during Ni-NTA based immobilized metal ion affinity chromatography (IMAC), with lanes label with M: the marker protein mix (PageRuler, pre-stained protein ladder, Thermo Fisher Scientific, Waltham, MA USA), Ü: representing the soluble fraction of the crude cell lysate, P: the insoluble pellet fraction of the crude cell lysate, D: the flow-through of the IMAC column after loading, W: the wash fraction and E_{NTA} the purified protein after IMAC. Relevant marker bands are label to allow estimation of protein molecular weights. AsLOV2 has a theoretical molecular weight of about 18 kDa (marked by red boxes in all gel pictures)

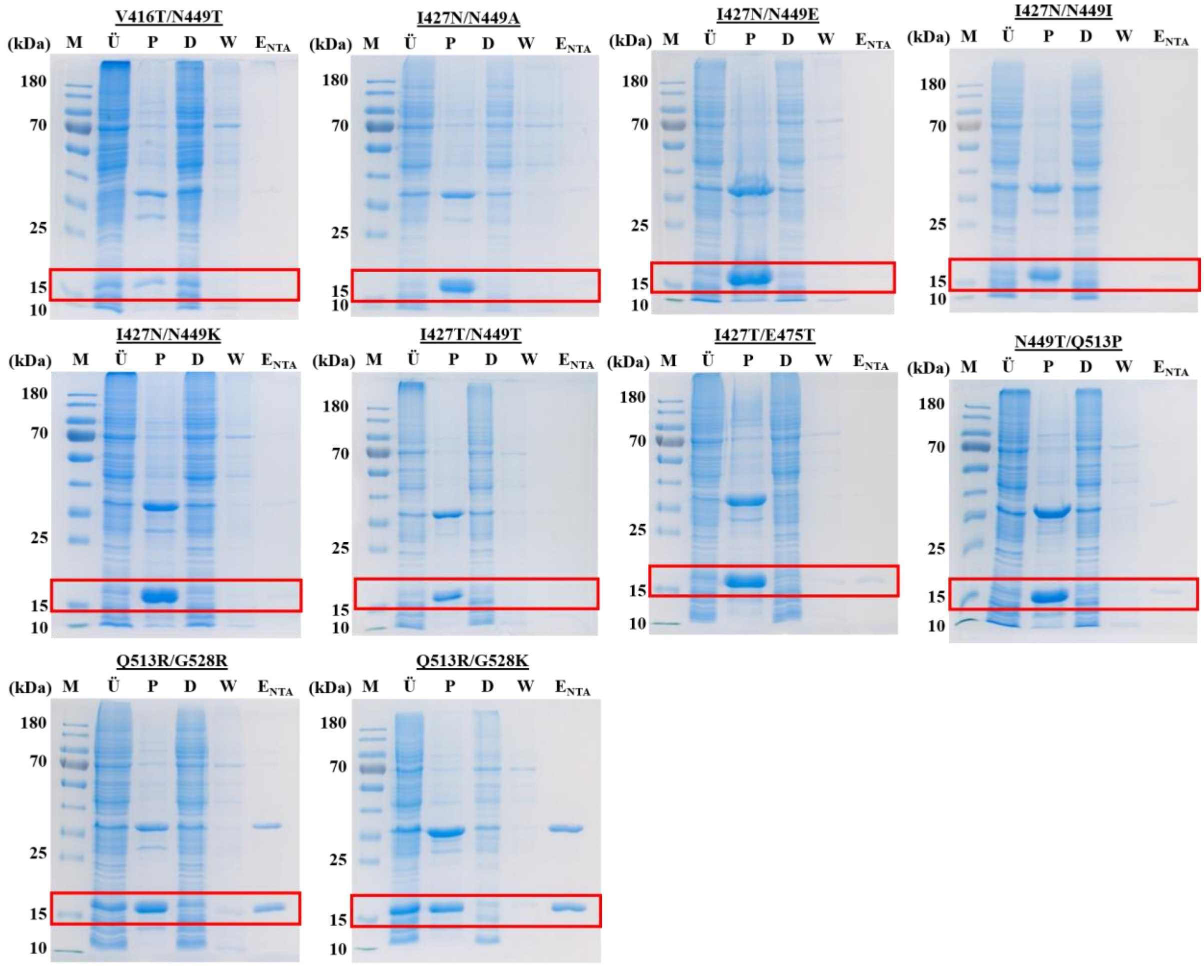

Figure S25: SDS-PAGE analysis of AsLOV2 variants generated, purified and characterized in this study. Each gel depicts the purification progress during Ni-NTA based immobilized metal ion affinity chromatography (IMAC), with lanes label with M: the marker protein mix (PageRuler, pre-stained protein ladder, Thermo Fisher Scientific, Waltham, MA USA), Ü: representing the soluble fraction of the crude cell lysate, P: the insoluble pellet fraction of the crude cell lysate, D: the flow-through of the IMAC column after loading, W: the wash fraction and E_{NTA} the purified protein after IMAC. Relevant marker bands are label to allow estimation of protein molecular weights. AsLOV2 has a theoretical molecular weight of about 18 kDa (marked by red boxes in all gel pictures)

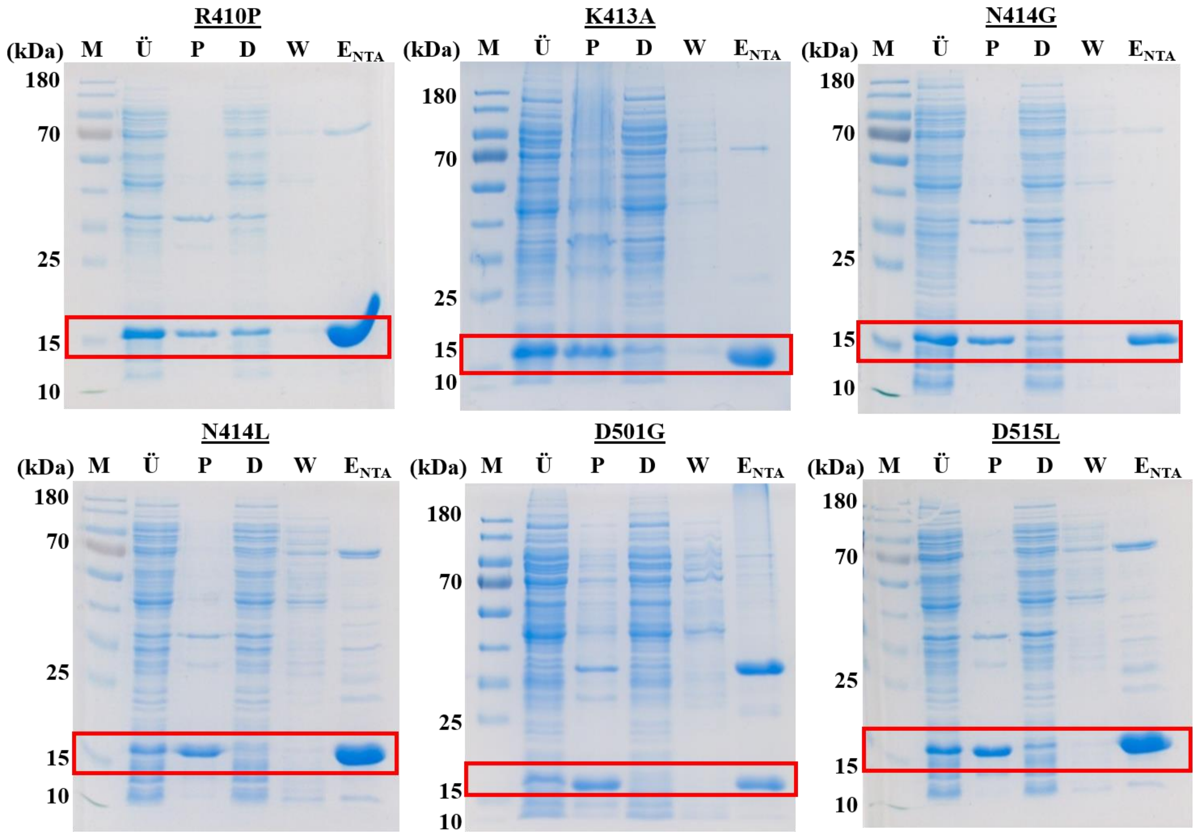

Figure S26: SDS-PAGE analysis of AsLOV2 variants generated, purified and characterized in this study. Each gel depicts the purification progress during Ni-NTA based immobilized metal ion affinity chromatography (IMAC), with lanes label with M: the marker protein mix (PageRuler, pre-stained protein ladder, Thermo Fisher Scientific, Waltham, MA USA), Ü: representing the soluble fraction of the crude cell lysate, P: the insoluble pellet fraction of the crude cell lysate, D: the flow-through of the IMAC column after loading, W: the wash fraction and E_{NTA} the purified protein after IMAC. Relevant marker bands are label to allow estimation of protein molecular weights. AsLOV2 has a theoretical molecular weight of about 18 kDa (marked by red boxes in all gel pictures)

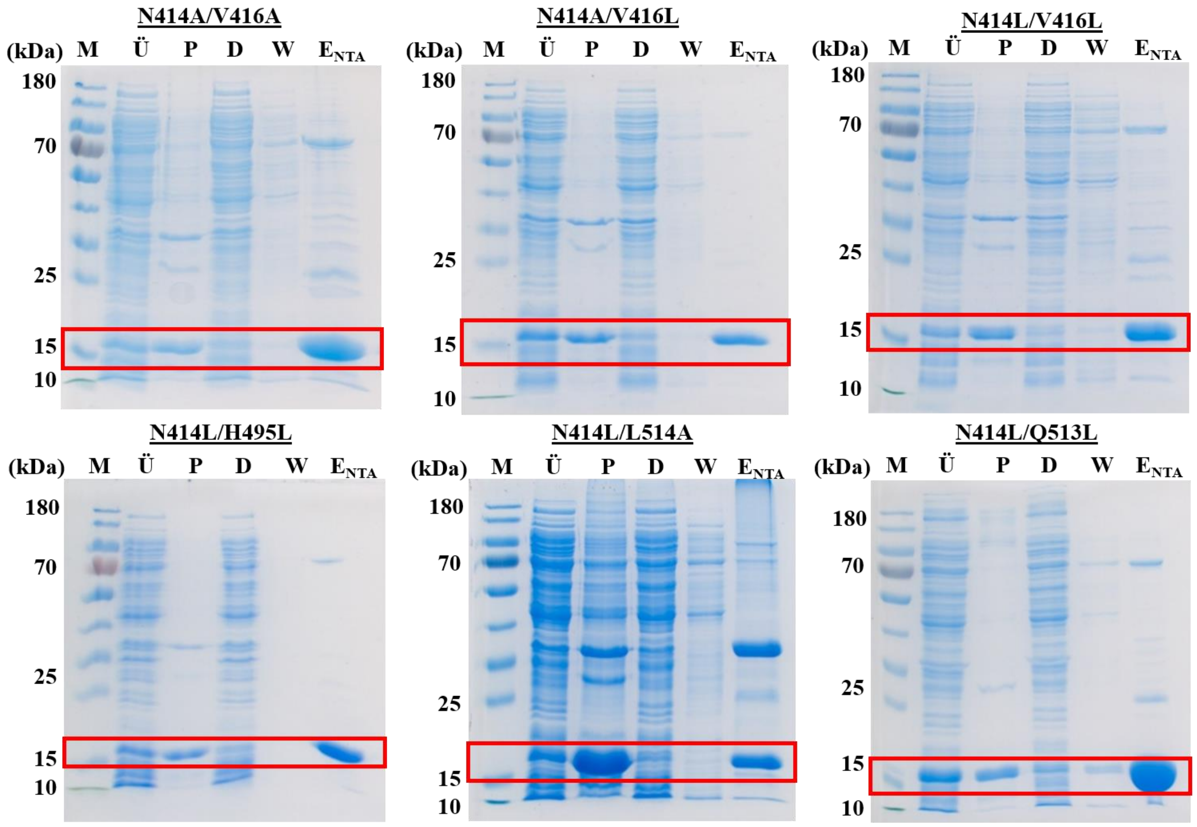

Figure S27: SDS-PAGE analysis of AsLOV2 variants generated, purified and characterized in this study. Each gel depicts the purification progress during Ni-NTA based immobilized metal ion affinity chromatography (IMAC), with lanes label with M: the marker protein mix (PageRuler, pre-stained protein ladder, Thermo Fisher Scientific, Waltham, MA USA), Ü: representing the soluble fraction of the crude cell lysate, P: the insoluble pellet fraction of the crude cell lysate, D: the flow-through of the IMAC column after loading, W: the wash fraction and E_{NTA} the purified protein after IMAC. Relevant marker bands are label to allow estimation of protein molecular weights. AsLOV2 has a theoretical molecular weight of about 18 kDa (marked by red boxes in all gel pictures)

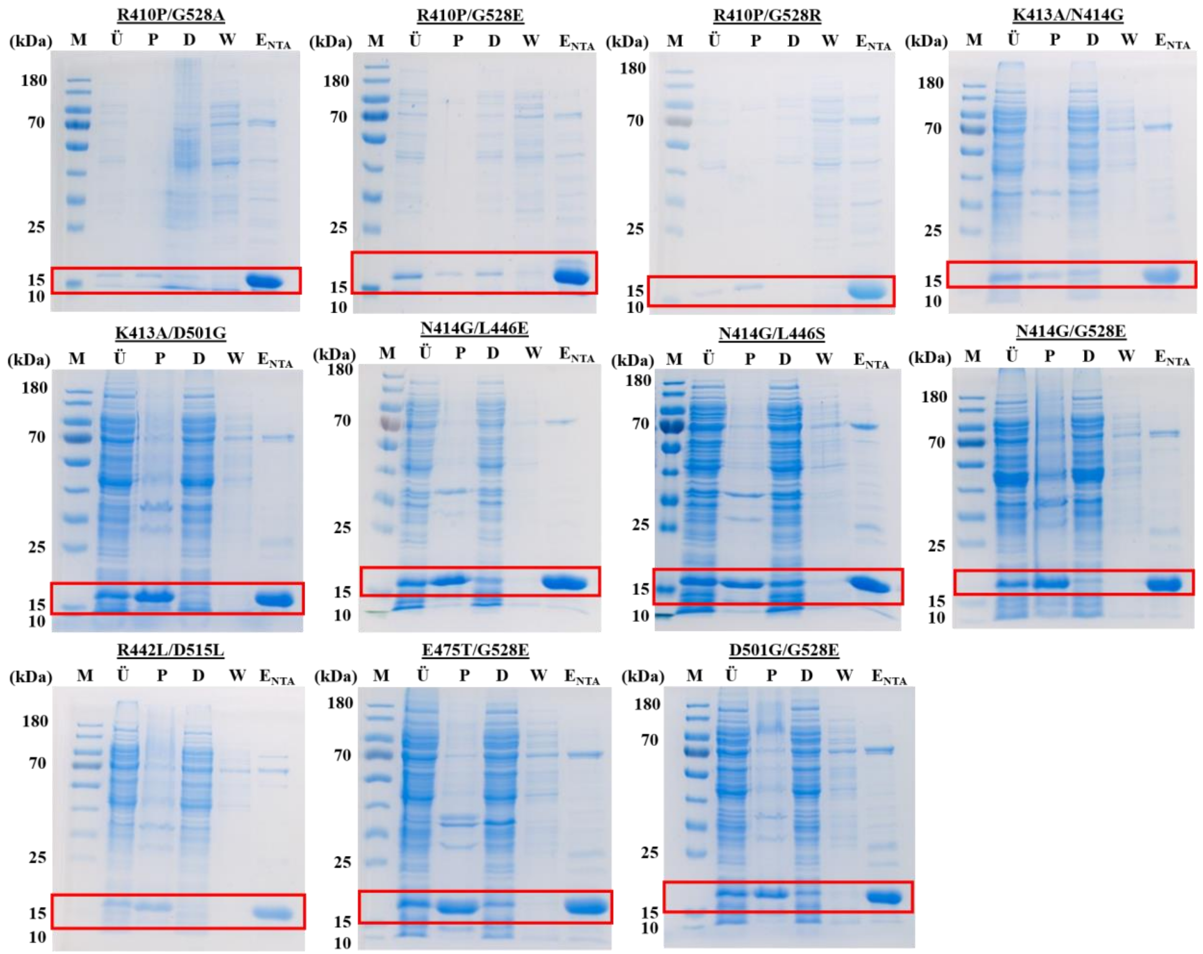

Figure S28: SDS-PAGE analysis of AsLOV2 variants generated, purified and characterized in this study. Each gel depicts the purification progress during Ni-NTA based immobilized metal ion affinity chromatography (IMAC), with lanes label with M: the marker protein mix (PageRuler, pre-stained protein ladder, Thermo Fisher Scientific, Waltham, MA USA), Ü: representing the soluble fraction of the crude cell lysate, P: the insoluble pellet fraction of the crude cell lysate, D: the flow-through of the IMAC column after loading, W: the wash fraction and E_{NTA} the purified protein after IMAC. Relevant marker bands are label to allow estimation of protein molecular weights. AsLOV2 has a theoretical molecular weight of about 18 kDa (marked by red boxes in all gel pictures)

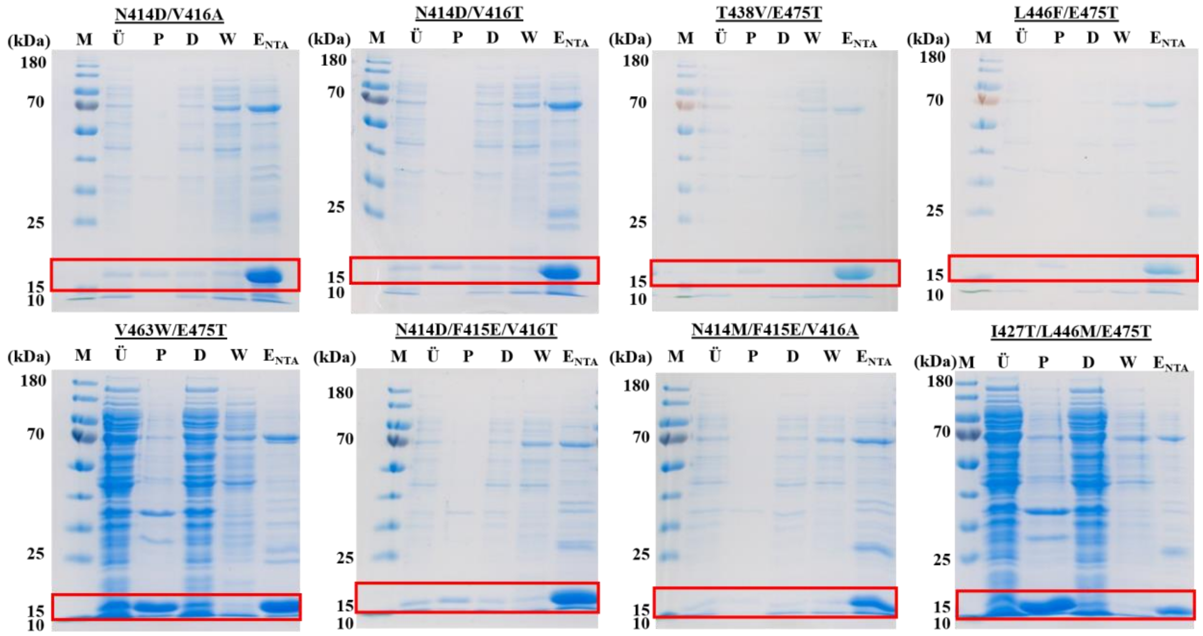

Figure S29: SDS-PAGE analysis of AsLOV2 variants generated, purified and characterized in this study. Each gel depicts the purification progress during Ni-NTA based immobilized metal ion affinity chromatography (IMAC), with lanes label with M: the marker protein mix (PageRuler, pre-stained protein ladder, Thermo Fisher Scientific, Waltham, MA USA), Ü: representing the soluble fraction of the crude cell lysate, P: the insoluble pellet fraction of the crude cell lysate, D: the flow-through of the IMAC column after loading, W: the wash fraction and E_{NTA} the purified protein after IMAC. Relevant marker bands are label to allow estimation of protein molecular weights. AsLOV2 has a theoretical molecular weight of about 18 kDa (marked by red boxes in all gel pictures)

Supporting References

1. Kawashima, S. and M. Kanehisa, *AAindex: amino acid index database.* Nucleic Acids Res, 2000. **28**(1): p. 374.
2. Siedhoff, N.E., et al., *PyPEF-An Integrated Framework for Data-Driven Protein Engineering.* J Chem Inf Model, 2021. **61**(7): p. 3463-3476.
3. Pedregosa, F., et al., *Scikit-learn: Machine Learning in Python.* The Journal of Machine Learning Research, 2011. **12**: p. 2825–2830.
4. Illig, M.-A., et al., *A hybrid model combining evolutionary probability and machine learning leverages data-driven protein engineering.* bioRxiv, 2022.
5. Hopf, T.A., et al., *Mutation effects predicted from sequence co-variation.* Nat Biotechnol, 2017. **35**(2): p. 128-135.
6. Morcos, F., et al., *Direct-coupling analysis of residue coevolution captures native contacts across many protein families.* Proc Natl Acad Sci U S A, 2011. **108**(49): p. E1293-301.
7. Luo, Y., et al., *ECNet is an evolutionary context-integrated deep learning framework for protein engineering.* Nat Commun, 2021. **12**(1): p. 5743.
8. Jing, X.Y., et al., *Amino Acid Encoding Methods for Protein Sequences: A Comprehensive Review and Assessment.* Ieee-Acm Transactions on Computational Biology and Bioinformatics, 2020. **17**(6): p. 1918-1931.
9. Zayner, J.P., C. Antoniou, and T.R. Sosnick, *The amino-terminal helix modulates light-activated conformational changes in AsLOV2.* J Mol Biol, 2012. **419**(1-2): p. 61-74.
10. Zayner, J.P., et al., *Investigating models of protein function and allostery with a widespread mutational analysis of a light-activated protein.* Biophys J, 2013. **105**(4): p. 1027-36.
11. Zayner, J.P. and T.R. Sosnick, *Factors that control the chemistry of the LOV domain photocycle.* PLoS One, 2014. **9**(1): p. e87074.
12. Zoltowski, B.D., B. Vaccaro, and B.R. Crane, *Mechanism-based tuning of a LOV domain photoreceptor.* Nat Chem Biol, 2009. **5**(11): p. 827-34.
13. Kawano, F., et al., *Fluorescence imaging-based high-throughput screening of fast- and slow-cycling LOV proteins.* PLoS One, 2013. **8**(12): p. e82693.
14. Christie, J.M., et al., *Steric interactions stabilize the signaling state of the LOV2 domain of phototropin 1.* Biochemistry, 2007. **46**(32): p. 9310-9.
15. Nash, A.I., et al., *A conserved glutamine plays a central role in LOV domain signal transmission and its duration.* Biochemistry, 2008. **47**(52): p. 13842-9.
16. Hemmer, S., et al., *Residues alterations within a conserved hydrophobic pocket influence light, oxygen, voltage (LOV) photoreceptor dark recovery.* in preparation, 2022.
17. Halavaty, A.S. and K. Moffat, *N- and C-terminal flanking regions modulate light-induced signal transduction in the LOV2 domain of the blue light sensor phototropin 1 from Avena sativa.* Biochemistry, 2007. **46**(49): p. 14001-9.
18. Woodcock, D.M., et al., *Quantitative evaluation of Escherichia coli host strains for tolerance to cytosine methylation in plasmid and phage recombinants.* Nucleic Acids Res, 1989. **17**(9): p. 3469-78.
19. Studier, F.W. and B.A. Moffatt, *Use of bacteriophage T7 RNA polymerase to direct selective high-level expression of cloned genes.* J Mol Biol, 1986. **189**(1): p. 113-30.
20. Mathes, T., et al., *In vivo generation of flavoproteins with modified cofactors.* J Mol Biol, 2009. **385**(5): p. 1511-8.
21. Hemmer, S., *Photozyklus und Signaltransduktion in light, oxygen, voltage (LOV) Photorezeptoren*, in *Faculty of Mathematics and Natural Sciences*. 2019, Heinrich-Heine-University. p. 153.
22. Chernomor, O., et al., *Complex Evolution of Light-Dependent Protochlorophyllide Oxidoreductases in Aerobic Anoxygenic Phototrophs: Origin, Phylogeny, and Function.* Mol Biol Evol, 2021. **38**(3): p. 819-837.
